# Introducing Disulfide Bonds into Polyester Biomaterials via Nucleophilic Thiol–yne Polymerization

**DOI:** 10.1021/acsmacrolett.5c00427

**Published:** 2025-09-08

**Authors:** Daniele Giannantonio, Meltem Haktaniyan, Arianna Brandolese, Maria C. Arno, Andrew P. Dove

**Affiliations:** † School of Chemistry, 1724 University of Birmingham, Edgbaston, Birmingham, B15 2TT, United Kingdom

## Abstract

Polyesters are a widely used class of biomaterials thanks to their (bio)­degradability and tunable thermomechanical properties. Introducing dynamic disulfide bonds into their backbone enables them to be degraded through different routes and also imparts self-healing properties. However, while numerous polymerization protocols exist with which to introduce disulfide bonds into linear polymers, these methods lack the versatility needed to produce materials with diverse thermomechanical properties. In this work, nucleophilic thiol–yne polymerization was employed to synthesize polymers with a controllable amount of disulfide bonds in the backbone. The crystallinity and, hence, the thermomechanical properties of these polymers were tuned through the variation of different parameters such as disulfide bond content, polymer backbone conformational features (*i.e*., *E*/*Z* isomerism), and monomer combinations. Moreover, polymer degradability was confirmed by leveraging the reversible disulfide bonds in the presence of chemical stimuli. Finally, the cytocompatibility of these polymers was demonstrated, suggesting their potential use as polymeric biomaterials.

## Graphical abstract

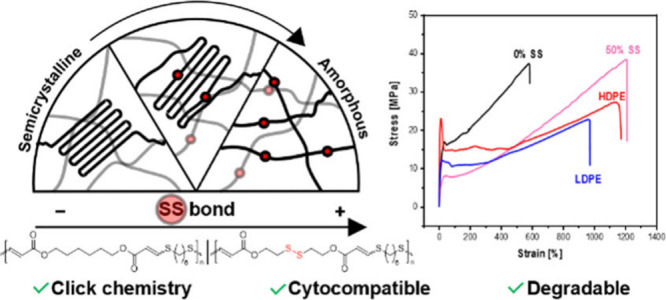

Polyesters have been widely studied as biomaterials owing to their simple synthesis, thermomechanical properties tuneability, and ready degradability *in vivo*.^1^ These materials have found applications in various fields, ranging from drug delivery systems to scaffolds for tissue engineering.^2,3^ Commonly used synthetic polymers belonging to this class are polyglycolide (PGA),^4,5^ polylactide (PLA),^6,7^ poly­(lactide-*co*-glycolide) (PLGA),^8–10^ and poly­(ε-caprolactone) (PCL).^11,12^ Notably, all these polymers are obtained through ring opening polymerization (ROP).

Although polyesters are inherently (bio)­degradable, their degradability and other properties, such as self-healing and shape memory, can be further improved by incorporating dynamic bonds into their backbone.^13,14^ The installation of dynamic disulfide bonds into synthetic polymers has attracted considerable interest as a consequence of their natural occurrence in biological systems.^15,16^ The dissociation energy of disulfide bonds is approximately 60 kcal mol^–1^ and provides a balanced combination of stability and dynamic behavior.^17,18^ Disulfide bonds have also received significant attention in the biomedical field. For example they have been used to develop polymeric drug carriers with controllable release,^19–21^ owing to their sensitivity to different redox environments, which makes them a useful tool to trigger anticancer agent delivery.^22,23^

Different strategies have been used to introduce disulfide bonds into polymers, spanning radical polymerizations, *e.g*., atom transfer radical polymerization (ATRP)^24,25^ and reversible addition–fragmentation chain transfer (RAFT),^26^ to nonradical processes, such as ROP,^27–31^ oxidation polymerization,^32^ polycondensation^33,34^ and polyaddition.^35–40^ Nonetheless, the trade-off between the inclusion of dynamic bonds and polymer thermomechanical properties has been scarcely investigated, possibly because these synthetic routes limit the possibility to control the polymer architecture in a simple manner, such as by tuning the monomers’ feed ratio. Although synthesizing polyesters *via* ROP is attractive owing to the ease of producing polymers with well-defined chain ends and predictable molar mass, structural modifications remain challenging because of monomer design constraints and the need for moisture- and oxygen-free conditions. Inversely, step-growth polymerization grants higher freedom of monomer choice, which translates into more diverse polymer structures being accessible. However, common step-growth polycondensation reactions usually require high temperatures and reduced pressure in order to shift the equilibrium toward product formation and reach high conversion, hence high molar mass.^41^ Polyaddition approaches can be conducted under milder conditions but are confined to few highly efficient reactions in order to ensure high molar mass polymers are obtained. Most commonly, these reactions are represented by polyurethanes, which require the use of toxic and moisture-sensitive isocyanates in their synthesis.^42–44^ Previous works from our group have shown that the organobase-catalyzed nucleophilic thiol–yne step-growth addition can be used to synthesize polyesters^45^ and polyamides^46^ of high molar mass, thus providing a facile route to access functional materials with useful thermomechanical properties. This “click” polyaddition approach is advantageous compared to traditional methods as it is high yielding, selective, and can be conducted in the presence of moisture and oxygen. Additionally, polyaddition reactions can occur without the need for heating and use nontoxic reagents, retaining high atom economy.^47^

Herein, we demonstrate that organobase-catalyzed nucleophilic thiol–yne step-growth polyaddition is compatible with the introduction of disulfide bonds into the polymer backbone. The crystalline phase of the resultant polymers can be precisely controlled by monomer choice as well as the *cis*/*trans* conformation of the resulting addition product.^48^ As such, diverse materials can be accessed from a limited pool of monomers, and the systematic evaluation of the influence of varying disulfide bond content is possible (Scheme 1). Finally, the cytocompatibility of a selection of these polymers was tested, revealing them to be non-toxic, thus suggesting this method to be a viable alternative to generate polyesters that are suitable for biomedical applications.

**Scheme 1:**
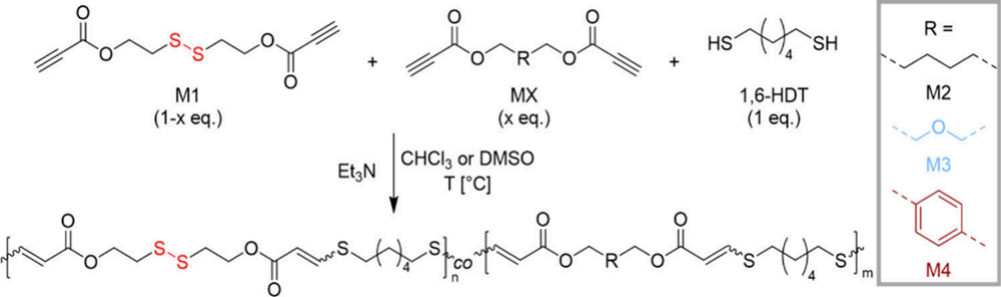
General Nucleophilic Thiol-yne Polymerization between Monomer M1, 1,6-Hexanedithiol (1,6-HDT), and MX^a^

a Structures of non-disulfide monomers MX used in this study are depicted in the box.

To access a range of polymers with controlled architecture and disulfide bond content, different dipropiolate monomers were synthesized and polymerized with 1,6-hexanedithiol (1,6-HDT) *via* nucleophilic thiol–yne step-growth polymerization. These monomers could all be easily obtained *via* Fisher esterification, adapting previously reported procedures^49–51^ using commercially available diols as starting materials. Structures were confirmed using proton (^1^H) and carbon (^13^C) nuclear magnetic resonance (NMR) spectroscopy and high-resolution mass spectrometry (HR-MS; see Supporting Information, Figures S1–S4).

As the nucleophilic thiol–yne reaction proceeds through the addition of a thiolate anion to an activated alkyne, it was crucial to find reaction conditions that would favor this process over the exchange of the thiolate with the disulfide bond within the monomer M1. To do so, a small molecule study reacting 2 equiv of 1-hexanethiol (1-HT) with 1 equiv of M1 was performed (Figure S5). Successful thiol–yne nucleophilic addition was confirmed by ^1^H NMR spectroscopy showing the disappearance of the resonances relative to the thiol proton (triplet, δ = 1.33 ppm) and the terminal alkyne proton (singlet, δ = 2.92 ppm), combined with the appearance of doublets at δ = 5.74 and 7.73 ppm that results from the formation of the vinyl moiety (85% *trans*) (Figure S6). Heteronuclear Single Quantum Coherence (HSQC) NMR spectroscopy was also used to confirm the structure of the addition product (Figure S7). The absence of unreacted thiols was further confirmed by Fourier transformed infrared spectroscopic (FTIR) analysis on the crude, which showed the absence of S–H stretching peak in the typical region between 2584–2598 cm^–1^ (Figure S8).^52^ Finally, mass spectrometry (MS) analysis evidenced the presence of the addition product, thus indicating that the addition of thiolates to the terminal alkynes is preferred over the thiol–disulfide exchange.

Our previous works identified solvent polarity and strength of the base as crucial in controlling the stereochemistry of the addition product of the nucleophilic thiol–yne reaction.^46,48,53^ Notably, these factors also greatly affect thiol–disulfide exchange.^54,55^ Given the solubility of the monomers, polymerization between M1 and 1,6-HDT was investigated in chloroform (CHCl_3_) and dimethylformamide (DMF), with triethylamine (Et_3_N) and 1,8-diazabicyclo[5.4.0]­undec-7-ene (DBU) as bases. A monomodal size exclusion chromatography (SEC) trace and a selective polymerization reaction, as observed by ^1^H NMR spectroscopy, were achieved using CHCl_3_ and Et_3_N (Figures S9 and S10). These results demonstrated the effectiveness of this solvent-base pair, leading to its selection for subsequent polymerization reactions.

Different polymer structures were obtained by varying the feeding ratio of monomer M1 and 1,6-hexanedipropiolate (M2), keeping a 1:1 equiv ratio between thiols and alkynes. Polymers with a M1 content ranging from 100% (PolyM1_100_) to 0% (PolyM2_100_) of the total dipropiolate monomer fed into the reaction were recovered in moderate to high yield (67–84%). ^1^H NMR spectroscopy analysis (Figures S11–S24) showed that all the polymers were *trans*-rich (>80%), as evidenced by the characteristic vinyl proton resonances at δ = 7.92–7.46 ppm (*J* = 15.2 Hz) and δ = 5.74–7.43 ppm (*J* = 15.2 Hz), which is characteristic of nucleophilic thiol–yne addition having occurred. The disulfide bond content varied with the M1 feed ratio, as indicated by the increased intensity of the disulfide methylene proton signal (δ = 2.9 ppm) in the ^1^H NMR spectrum (Figure S25). Furthermore, ^13^C NMR spectroscopy showed the characteristic disulfide bond ^β^C signal at δ > 35 ppm only in polymers containing M1, thus confirming the presence of the disulfide bond in the backbone of the polymers (Figure S26).^56^ SEC analysis was employed to characterize weight average molar mass (*M*_W_) and dispersity index (*Đ*_M_), revealing high molar masses (up to 103 kg·mol^–1^) and relatively narrow dispersities (1.6 ≤ *Đ*_M_ ≤ 3.6; Figure S27, Table 1).

**Table 1:** Summary of *M*_W_, *Đ*_M_, and Thermomechanical Properties of Polymers Having Different Amounts of Disulfide Bonds in the Backbone

name	*M*_W_ [kg·mol^–1^]^a^	*Đ*_M_ ^a^	*T*_g_ ^b^ [°C]	*T*_c_ ^c^ [°C]	*T*_m_ ^d^ [°C]	*T*_d,5%_ ^e^ [°C]	Young’s modulus, *E* [MPa]	stress at break, σ [MPa]	strain at break, ε [%]	strain energy density, *U*_T_ [J·m^–3^]
PolyM2_100_	102.9	3.59	–20.4	3.82	99.71	346.8	173.3 ± 6.3	35.6 ± 1.6	560.7 ± 19.1	133.5 ± 9.5
PolyM1_10_M2_90_	95.9	2.89	–21.0	10.7	95.05	335.1	128.7 ± 21.4	36.3 ± 5.5	733.1 ± 72.8	167.9 ± 37.8
PolyM1_25_M2_75_	43.8	2.79	–22.0	17.9	88.9	307.3	107.4 ± 10.7	25.2 ± 0.5	585.7 ± 3.4	100.5 ± 4.3
PolyM1_50_M2_50_	76	2.50	–18.9	n.a.	n.a.	308.9	45.5 ± 1.8	31.8 ± 8.8	1099.6 ± 157.9	199.2 ± 64.5
PolyM1_75_M2_25_	60.2	1.60	–17.9	n.a.	n.a.	303.8	n.a.	n.a.	n.a.	n.a.
PolyM1_90_M2_10_	56.9	2.69	–16.7	n.a.	n.a.	306.5	n.a.	n.a.	n.a.	n.a.
PolyM1_100_	64.1	2.79	–16.0	n.a.	n.a.	300.5	n.a.	n.a.	n.a.	n.a.

a Value obtained by SEC in chloroform (0.5% Et_3_N), comparison with polystyrene standard (*M*_p_ = 162–3187000 g·mol^–1^).

b Value determined by the inflection point in the second heating cycle of DSC (N_2_ atmosphere, −80 to 130 °C, 10 °C·min^–1^).

c Calculated taking the maximum of the exothermic peak in the second heating cycle of DSC.

d Calculated taking the minimum of the endothermic peak in the second heating cycle of DSC.

e Taken as the temperature associated with a 5% mass loss in the TGA (25 to 600 °C, 10 °C·min^–1^, N_2_ atmosphere).

To investigate the influence of disulfide bond content on the thermal properties of the polymers, thermogravimetric analysis (TGA) and differential scanning calorimetry (DSC) were conducted. The degradation temperature was taken as the temperature associated with a mass loss of 5% (*T*_d,5%_), and all polymers displayed thermal stability under N_2_ up to 300 °C or above (Table 1, Figure S28). Increasing the amount of disulfide bonds led to a decrease in the *T*_d,5%_ of the polymers, from 346 to 300 °C, consistent with the weaker energy associated with it when compared to C–C bonds.^17^ DSC analysis showed that only polymers having an M1 content of 0.25 equiv or less displayed an endothermic peak (*i.e*., melting transition) in the second heating cycle, indicating that the crystallinity of these polymers decreased with increasing M1 content. (Figures S29–S35). The melting temperature range detected for these polymers is between 88 and 99 °C (Figure 1A). Enthalpy of melting (Δ*H*_m_^0^) values calculated for polyM2_100_, polyM1_10_M2_90_, and polyM1_25_M2_75_ were, respectively, −37.9, −41.7, and −38.4 J·g^–1^. Exothermic peaks indicating cold crystallization upon heating^57^ were present at temperatures increasing from 4 to 18 °C with increasing content of M1, up to 0.25 equiv. These data suggested that M2 was responsible for the crystallization of the polymer, and its ordering was hindered by increasing the amount of M1. Given the structural similarity between M1 and M2, it is likely that the presence of bulkier sulfur atoms can disrupt the alignment of polymer chains.^58^ Polymers having an M1 of 50% or above did not display any melting peak and resulted in being amorphous. However, it is worth noting that both polyM1_50_M2_50_ and polyM1_75_M2_25_ displayed an endothermic peak during the heating of the first cycle of the DSC measurement (Figures S32 and S33), meaning that annealing of these polymers could lead to the formation of crystalline domains. The glass transition temperature (*T*_g_) increased slightly with increasing M1 content (from −20 to −16 °C) (Figure 1A). These changes in crystallinity are reflected in the mechanical properties, tested *via* uniaxial tensile testing after heating compression molding of the polymers into free-standing films. ^1^H NMR spectroscopy and SEC analysis were conducted on the heat-pressed polymers, showing little to no change after the molding process, proving the stability of the internal alkene bonds and disulfides (Figures S36–S39). Polymers having an M1 content of 75% or above with regard to the total alkyne content resulted in being too soft to be reliably tested by this method (Figure S44, Table 1). Nonetheless, the materials tested showed different mechanical properties, with Young’s modulus values ranging from 173 to 45 MPa when the amount of disulfide bond was increased. Interestingly, increasing disulfide content did not seem to notably affect the ultimate tensile strength of the materials, which is comparable to that of high-density polyethylene (HDPE) (Figure 1B).^59,60^ The increase in strain, hence strain energy density, that was observed with increasing disulfide bond content could be explained by the disulfide bonds acting as energy dissipators as a consequence of their lower bond strength. The same phenomenon was also observed in polyesters synthesized by polycondensation and epoxy thermosets.^34,61^ Finally, when compared to polyesters possessing disulfide bonds produced by polycondensation,^34^ our polymers displayed lower Young’s moduli (45–175 MPa *vs* 100–400 MPa), but higher strain (up to 1000% *vs* 300%) and stress at break (up to 36 MPa *vs* 34 MPa) on average. These results could stem from the presence of stronger double carbon–carbon bonds in the polymer backbone. The lower strain at break and toughness observed for polyM1_25_M2_75_ is justified by its lower molar mass when compared to the other polymers. A similar behavior was also observed when lower *M*_w_ polyM2_100_ and polyM1_10_M2_90_ were synthesized, calculating the stoichiometric imbalance using the extended Carothers’ equation (Figures S46 and S47).^41^ Shorter polymer chains resulted in an increased Young’s modulus, attributed to enhanced crystallinity; however, this is typically accompanied by a decrease in the maximum strain as chain entanglement is reduced.^62^ The lack of significant difference in terms of crystallinity observed in polymers that have different *M*_w_ but the same structure further suggests that the different sulfur content is responsible for the distinctive crystallization behavior and thermomechanical properties.

**Figure 1:**
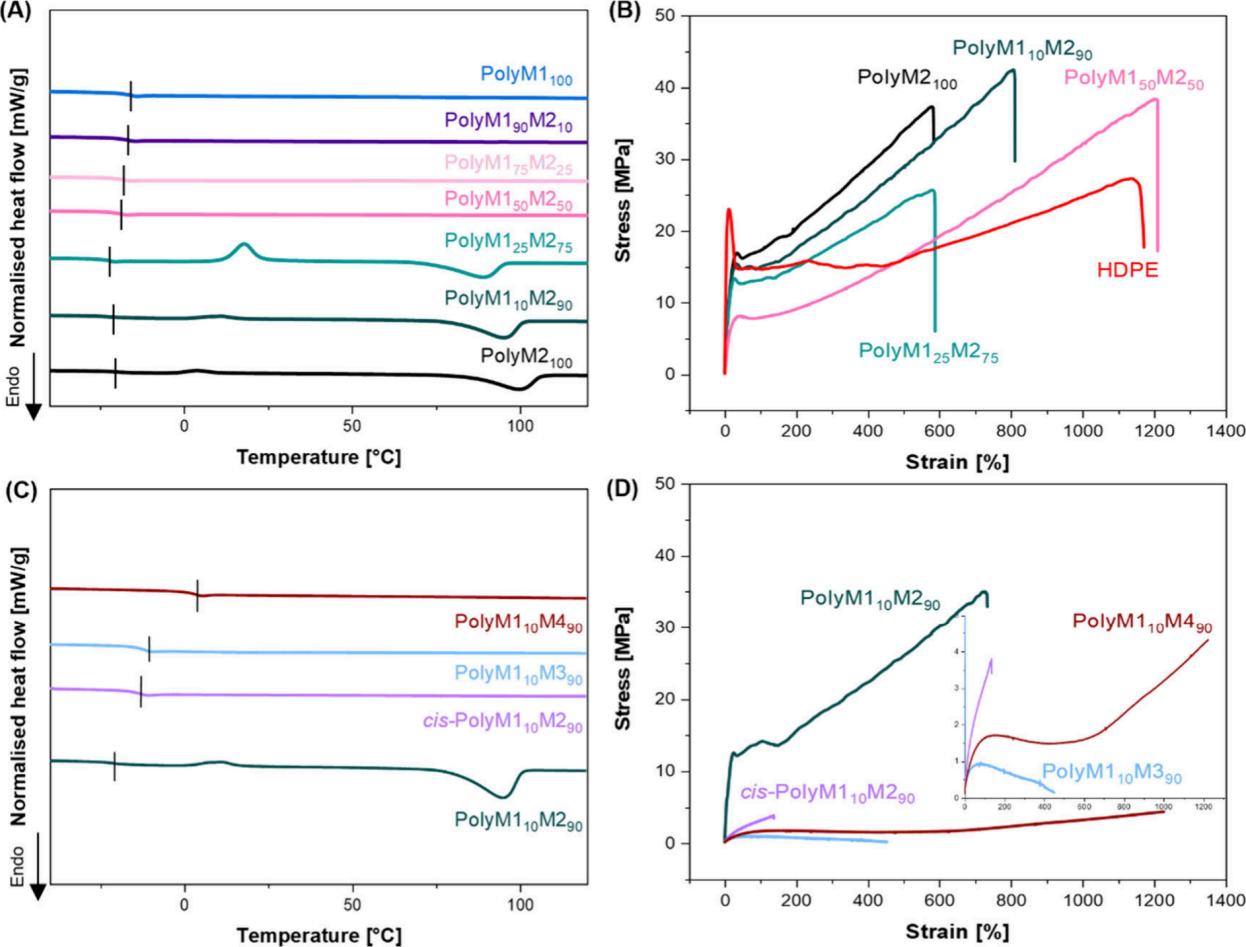
(A) DSC thermograms (second heating scan, −40 to 130 °C, 10 °C·min^–1^) of different polymers synthesized varying the M1/M2 ratio. (B) Exemplary stress vs strain curves obtained by uniaxial tensile test (10 mm·min^–1^, 22 °C) of different polymers having different M1/M2 ratio. (C) DSC thermograms (second heating scan, −30 to 130 °C, 10 °C·min^–1^) of different polymers synthesized keeping M1 content at 0.1 equiv to the equivalent of thiol used. (D) Exemplary stress *vs* strain curves obtained by uniaxial tensile test (10 mm min^–1^, 22 °C) of polymers obtained by keeping the M1 content fixed and varying co-monomer.

To evaluate the influence of monomer chemistry on polyesters that have a fixed amount of disulfide bond in their backbone, the synthesis and characterization of high-*cis* polymer and polymers having non-aliphatic co-monomers were performed. The influence of *cis*/*trans* ratio on the thermomechanical properties was investigated by synthesizing a high *cis* content polyM1_10_M2_90_ (*M*_n_ = 11 kg mol^–1^, *Đ*_M_ = 4.9) using a more polar solvent, *i.e*., dimethyl sulfoxide (DMSO).^46,53^ The slightly lower *M*_n_ and higher *Đ*_M_ obtained with these reaction conditions is consistent with those reported previously,^54,63^ and result from a higher rate of disulfide metathesis happening in a more polar environment, as observed during reaction condition screening (Figures S9 and S10). The *cis* content of this polymer, evaluated by the vinyl proton resonance in the ^1^H NMR spectrum (Figure S50), was found to be around 67%. TGA analysis revealed a moderately lower degradation temperature (335 °C for the high *trans* polymer and 312 °C for the high *cis*) that can be explained by the lower stability associated with *cis* linkages when compared to *trans*. Remarkably, differences in crystallinity were observed by DSC (Figure 1C). The high *cis*-content polymer was completely amorphous, as shown by the absence of a melting peak in the DSC trace. This difference in crystallinity was reflected in the high *cis* polymer displaying reduced stretchability and strength when tested *via* tensile testing (Figures 1D and S53). The *T*_g_ of this material was higher than that of the high *trans* polymer, – 13.9 °C vs. – 21.0 °C. Modulation of crystallinity was also achieved by switching M2 for two other monomers, a diethylene glycol derivative (M3) and a *p*-xylylene glycol dipropiolate (M4) (Scheme 1). These monomers were designed to evaluate the impact of introducing another heteroatom (presence of oxygen in M3) while maintaining similar monomer length and to assess the impact of reduced monomer flexibility while introducing noncovalent interactions (*i.e*., π-π stacking, M4). Polymer structures and *M*_W_ were elucidated using ^1^H NMR spectroscopy and SEC analysis (Figures S55–S58, Table S1). TGA revealed comparable degradation temperature (between 327 and 335 °C) (Figure S61, Table S1), while DSC confirmed the reduced crystalline phase, stressing the importance of monomer choice when designing polymeric materials. *T*_g_s of these polymers were found to increase by switching from M2 to M3 and M4 (Figure 1C). This increase can be explained by the reduced free volume between polymer chains caused by increased noncovalent interactions between chains and decreased flexibility; the same trend is observed for polyethylene, poly­(ethylene oxide), and poly­(*p*-xylylene).^64–66^ Both materials resulted considerably softer when compared to polymers having the same amount of M1 in the backbone but synthesized using M2, highlighting the importance of the crystalline phase in obtaining strong materials (Figure 1D). Notably, polymers synthesized using M4 exhibited improved elongation at break, potentially due to the presence of π–π stacking interactions.^67^

To demonstrate that degradation can proceed in a controlled manner under mild conditions, reduction using dithiothreitol (DTT), a widely used disulfide-reducing agent, was carried out (Figure 2A).^68^ Degradation of polyM1_25_M2_75_ and polyM1_75_M2_25_ was followed by SEC, and the reaction was stopped after 24 h. This analysis showed that the degradation of polyM1_25_M2_75_ yielded fragments with a *M*_w_ of approximately 9 kg·mol^–1^, about one-quarter of the original *M*_w_, indicating that the incorporation of the different monomers occurs statistically in line with the feed ratio (Figure 2B). Degradation of polyM1_75_M2_25_ yielded smaller fragments of *M*_w_ around 2 kg mol^–1^, consistent with the presence of shorter nondegradable moieties in the main chain as the amount of degradable monomer was increased (Figure 2C). Notably, since the amount of disulfide bonds in the backbone depends on the monomer feeding ratio, which also influences the mechanical properties, there is a trade-off between material strength and the extent of disulfide-mediated degradability. As polyesters represent an important class of biomaterials, potential disulfide reduction caused by antioxidants such as glutathione (GSH) must be considered in this context.^69^ Since these materials display high strain at break and ductility, we suggest that they would be more suitable for external applications, *e.g*. skin patches, rather than internal nanomedicine. Given the lower GSH concentration in extracellular environment when compared to cytoplasmic concentration (micromolar *vs* millimolar),^70^ reductive degradation should be limited. Moreover, this could still be exploited as delivery system.^71^ Potential structures resulting from the degradation of these polymers include 1,6-hexanediol, which is considered non-toxic, β-mercaptoethanol, which can act as an antioxidant, and α,β-unsaturated aliphatic dicarboxylic acids with a heteroatom (S) at the γ position. Molecules similar to the latter can be reasonably expected from materials previously tested *in vivo* that displayed satisfactory compatibility.^45^ To this extent, preliminary cytocompatibility studies were conducted on polyM2_100_, polyM1_10_M2_90_, polyM1_25_M2_75_, and polyM1_50_M2_50_ *via* PrestoBlue assay. Cell proliferation was evaluated over a 7-day period using HEKa cells (human epidermal keratinocytes). All polymers were found to be highly cytocompatible, with an ability to sustain cell proliferation equal to or higher than uncoated glass (Figure 2D). Finally, cell spreading on the polymer coatings was also evaluated through confocal fluorescence microscopy, by staining cell nuclei and cytoskeleton. No significant differences in cell spreading and morphology were observed between polymer-coated samples and controls, demonstrating that HEKa cells are able to adhere and grow as expected on the polymer substrate (Figure 2E).^72,73^ Importantly, these experiments were performed in subconfluent conditions, to allow cells enough space for spreading and ensure single-cell imaging.

**Figure 2:**
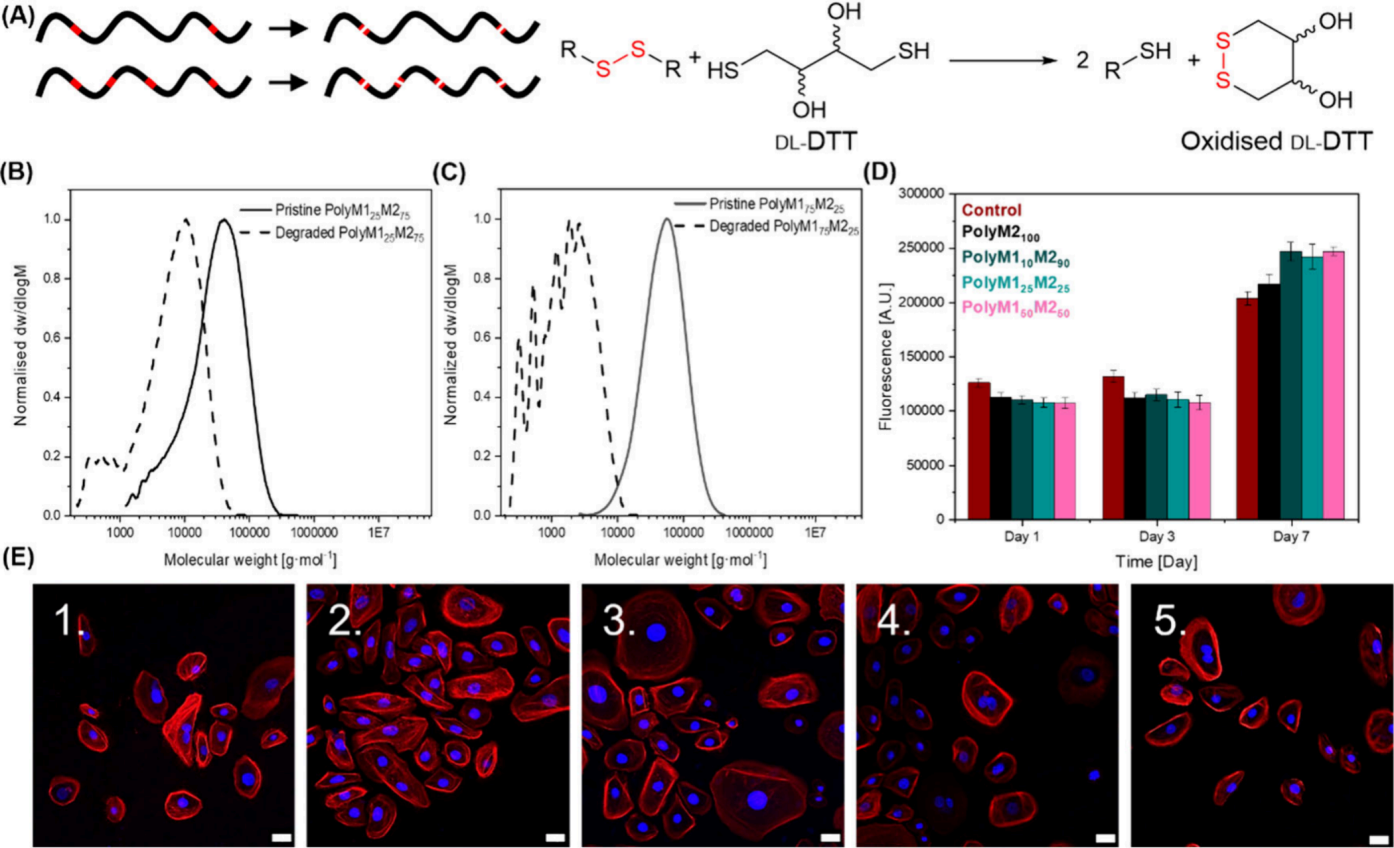
(A) Cartoon representing the influence of different disulfide bonds concentration on the length of the resulting chain (left) after DTT-mediated reduction (right). SEC (CHCl_3_, 0.5% Et_3_N, against polystyrene standard) traces of polyM1_25_M2_75_ (B) and polyM1_75_M2_25_ (C) before (full line) and after (dashed line) degradation *via* DTT mediated reduction (reaction time = 24 h). (D) Cell viability determined *via* PrestoBlue assay. (E) Representative confocal fluorescence images of HEKa cells cultured on spin coated films of polyM1_10_M2_90_ (1.), polyM1_25_M2_75_ (2.), polyM1_50_M2_50_ (3.), polyM2_100_ (4.), and uncoated glass slides (5.). Cells are stained with DAPI (nucleus, blue) and rhodamine phalloidin (cytoskeleton, red). Scale bar = 20 μm.

In conclusion, this work demonstrates the compatibility of nucleophilic thiol–yne step-growth polymerization with monomers having aliphatic disulfide bonds by synthesizing a library of degradable polyesters. Compared to previously reported methods, this polymerization strategy allows for greater control over the crystalline phase of the resulting polymers, meaning that a wide range of thermomechanical properties can be accessed from a small pool of monomers and thoughtful design. Notably, some of these polymers displayed thermomechanical properties comparable to HDPE albeit presenting handles for degradability in their backbone. Moreover, these materials were found to be cytocompatible, suggesting this method of polymerization can be used as a simple tool for the fabrication of tunable biomaterials.

## Supplementary Material



## Notes

Additional experimental details, ^1^H and ^13^C NMR spectra for all compounds, SEC data, and thermomechanical chatacterization for all polymers (PDF)

## ‡

Çukurova University, Faculty of Arts and Sciences, Chemistry Department, 01250, Sarıçam, Adana, Turkey
