# Supplementary material for: Introducing Disulfide Bonds into Polyester Biomaterials via Nucleophilic Thiol–yne Polymerization
Source: ACS Macro Lett. 2025 Sep 8;14(10):1359–66. doi: 10.1021/acsmacrolett.5c00427 (PMC12548359; doi:10.1021/acsmacrolett.5c00427)
Supplement: Supplementary file 1 [file mz5c00427_si_001.pdf]

Supporting Information

Introducing disulfide-bonds into polyester biomaterials *via* nucleophilic thiol-yne polymerization

Daniele Giannantonio[a], Meltem Haktaniyan[a], Arianna Brandolese[a], Maria C. Arno[a], Andrew P. Dove[a*]

[a]*School of Chemistry, University of Birmingham, Edgbaston, Birmingham, B15 2TT (UK)*

Contents

Experimental methods 3

NMR spectroscopy of synthesized monomers..14

Characterization data of small molecules study 17

^{1}H NMR spectra of solvent/catalyst screening 21

SEC traces of solvent/catalyst screening 22

^{1}H NMR & ^{13}C NMR spectra and SEC traces of *trans* polymers with varying amount of monomer M1 23

Thermal characterization of *trans* polymer of different monomer M1 content..38

Influence of compression molding on polymer structure and M_W 46

Mechanical characterization of *trans* polymers of different monomer M1 content 48

Evaluation of the influence of M_W on thermomechanical properties 51

Synthesis and characterization of *cis*-polyM1$_{10}$M2$_{90}$ 54

Thermomechanical characterization of *cis*-polyM1$_{10}$M2$_{90}$..................................56

Synthesis and characterization of polymers of different architecture with a fixed content of monomer M1 58

Thermomechanical properties of polymers of different architectures with fixed content of monomer M1 60

References 65

Experimental methods

All compounds, unless otherwise indicated were purchased from commercial sources and used as received. The following chemicals were vacuum distilled prior to use and stored in Young's tapped ampoules under N_2: 1,6-hexanedithiol (Sigma-Aldrich, ≥ 97%), propiolic acid (Sigma-Aldrich ≥ 97%). Heating was applied through a stirring plate equipped with an oil bath. Monomer M1 and monomer M2 have been synthesised following a procedure precedently developed withing the group.[1]

NMR Spectroscopic Analysis. All NMR spectroscopy experiments were performed at 300 K on a Bruker DPX-400 NMR instrument equipped with a BBFO smart probe operating at 400 MHz for H (100.57 MHz for ^{13}C). ^{1}H NMR spectra are referenced to solvent residual proton (δ = 7.26 for $CDCl_3$) and ^{13}C NMR spectra are referenced to the solvent signal (δ = 77.16 for $CDCl_3$). The resonance multiplicities are described as s (singlet), d (doublet), t (triplet), q (quartet), p (pentet), dd (doublet of doublets), dt (doublet of triplets), td (triplet of doublets), dq (doublet of quartets), ddd (doublet of doublet of doublets), dtd (doublet of triplet of doublets) or m (multiplet).

Mass Spectrometry. High Resolution Electrospray Ionization Mass Spectrometry was performed in the School of Chemistry at the University of Birmingham on a Waters Xevo G2-XS QTof Quadrupole Time-of-Flight mass spectrometer.

Fourier-transform Infrared Spectroscopy (FTIR): All FTIR spectroscopic analyses were performed on an Agilent Technologies Cary 630 FTIR spectrometer at a resolution of 4 cm^{-1}. 16 Scans from 600 to 4000 cm^{-1} were performed and the spectra were corrected for background absorbance.

Size Exclusion Chromatography (SEC). SEC measurements were performed in $CHCl_3$ on an Agilent 1260 Infinity II Multi-Detector SEC System fitted with RI, ultraviolet

(UV, λ = 309 nm), and viscometer detectors. The polymers were eluted through an Agilent guard column (PLGel 5 μM, 50 × 7.5 mm) and two Agilent mixed-C columns (PLGel 5 μM, 300 × 7.5 mm) using $CHCl_3$ (buffered with 0.5% Et_3N) as the mobile phase (flow rate = 1 mL·min^{-1}, 40 °C). Number average molecular weights (M_n), weight average molecular weights (M_w) and dispersities ($Đ_M = M_w/M_n$) were determined using Agilent GPC software (vA.02.01) against a 10-point calibration curve (M_p = 162–3,187,000 g·mol^{-1}) based on poly(styrene) standards (Easivial PSM/H, Agilent).

Differential Scanning Calorimetry (DSC). The thermal characteristics of the polymers were determined using differential scanning calorimetry (STARe system DSC3, Mettler Toledo) from −80 to 120 °C at a heating rate of 10 °C·min^{-1} for three heating/cooling cycles unless otherwise specified. The glass transition temperature (T_g) was determined from the inflection point in the second heating cycle of DSC. Total enthalpy of melting (ΔH_m) was calculated from the integration and normalization of all endothermic peaks present using the STARe software. The melting temperature was determined from the minimum value of the endothermic peak using the STARe software.

Thermogravimetric Analysis (TGA). TGA thermograms were obtained using a Q550 Thermogravimetric Analyzer (TA instrument). Thermograms were recorded under an N_2 atmosphere at a heating rate of 10 °C·min^{-1}, from 10 to 600 °C, with an average sample weight of *ca*. 5 mg. Aluminium pans were used for all samples. Decomposition temperatures were reported as the 5% weight loss temperature ($T_{d,5\%}$).

Tensile testing. Samples were prepared into thin polymer films using 3 Specac Atlas™ Manual Hydraulic Press 15T fitted with Specac heated plates. The polymer was added into a 40 × 50 × 0.50 mm stainless steel mold and placed into pre-heated

press at 120 °C. The sample was heated for 10 min (with no pressure) before degassing 5 × (500 kg pressure/release). Next, 2000 kg pressure was applied, and the sample was held at 120 °C for 5 min before cooling to ~25 °C under the same pressure. Film samples were visually inspected for deformation and bubbles. Finally, dumbbell shaped samples were cut using custom ASTM Die D-638 Type 5. Uniaxial tensile testing was performed using a Testometric M350-5CT universal mechanical testing instrument fitted with a load cell of 10 kN. Each specimen was clamped into the tensile holders and subjected to an elongation rate of 10 $mm \cdot min^{-1}$ until failure with an applied pretension of 0.1 N. All tensile tests were repeated 3 times (unless otherwise stated), and an average of the data was taken to find the ultimate tensile stress and strain. Data was analyzed using winTest™ Analysis software (v.5.0.34) and OriginPro® software. Young modulus was calculated by taking the slope of the tensile curve at 1% strain. Toughness was calculated as the area under the curve up to break.

Degradation experiment: Disks cut out from the heat pressed samples (8 mm diameter, 0.5 mm height) were placed in $CHCl_3$ with a final concentration of 10 mg/mL in a 7 mL vial. SEC analysis was performed to evaluate the presence of oligomers after the addition of the solvent and after a 24-hour period revealing the absence of degradation. Dithiothreitol (DTT) (100 equiv. to disulfide equivalent) was added to the vials that were stirred on an orbital shaker at 250 rpm until the films were not visible anymore (*ca.* 24 h). Aliquotes (50 µL) were taken throughout the experiment to monitor the degradation *via* SEC. The experiment was conducted at room temperature.

Synthetic procedures

Diethylene glycol dipropiolate (M3): Propiolic acid (8.26 g, 117.9 mmol, 1 eq.),

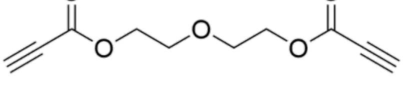

diethylene glycol (5 g, 47.1 mmol, 2.5 eq.), and *para*-

toluensulfonic acid monohydrate (*p*-TsOH) (0.45 g, 2,4 mmol, 0.05 eq.) were solubilized in toluene (200 mL) in a 500 mL round bottom flask equipped with a Dean-Stark apparatus under constant stirring. The temperature of the mixture was increased until reflux occurred, and the reaction was left overnight (*ca.* 16 h). The reaction mixture was allowed to cool down to room temperature and concentrate under reduced pressure to obtain an orange oil that was distilled using a Kugelrohr set up to obtain a slightly yellow liquid that solidifies upon standing. Solid was recrystallized from hexane to obtain product M**3** as white crystals (4.13 g, 42% yield) with spectroscopic data in agreement with previously published results.[2]

^{1}H NMR (400 MHz, Chloroform-*d*) *δ* 4.45 – 4.25 (m, 4H), 3.79 – 3.70 (m, 4H), 2.90 (s, 2H). ^{13}C NMR (101 MHz, Chloroform-*d*) *δ* 152.9, 74.7, 66.3, 28.3, 25.5. HRMS (TOF-MS-EI^{+}) (m/z): $[M+Na]^{+}$ calculated for $C_{10}H_{10}O_5Na$, 233.0426; found, 233.0429.

1,4-benzyl dipropiolate (M4): Propiolic acid (1.27 g, 18.1 mmol, 2.5 eq.), 1,4 – benzene dimethanol (1.0 g, 7.24 mmol, 1 eq.), and *p*-TsOH (0.138 g, 0.72 mmol, 0.1 eq.) were solubilized in toluene (200 mL) in a 500 mL round bottom flask equipped with a Dean-Stark apparatus under constant stirring. The temperature of the mixture was increased until reflux occurred, and the reaction is left going overnight (*ca.* 16 h). The reaction mixture was concentrated under reduced pressure and the product was purified *via* column chromatography (Hex:EtOAc 3:1) and obtained as a white solid (0.411 g, 23%) with spectroscopic data in agreement with previously published results.[3]

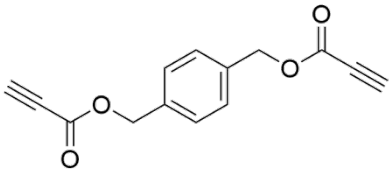

^{1}H NMR (400 MHz, Chloroform-*d*) *δ* 7.40 (s, 4H), 5.22 (s, 4H), 2.90 (s, 2H). ^{13}C NMR (101 MHz, Chloroform-*d*) *δ* 152.6, 135.3, 128.9, 75.4, 74.6, 67.5. HRMS (TOF-MS-EI^{+}) (m/z): $[M+Na]^+$ calculated for $C_{14}H_{10}O_4Na$, 265.0447; found, 265.0482.

General procedure for polymer synthesis of high *trans* polymer

1,6-hexanedithiol (1 eq.) was weighed in a 20 mL scintillation vial. Monomer M1 (x eq.) and the desired non-disulfide containing monomer (M2 or M3 or M4) (1-x equivalent) were weighed in different vials according to the desired degree of monomer M1 in the final polymer. Monomers were then transferred to the vial containing the dithiol. The final concentration of the thiol was kept at 0.5 M. Reaction mixture was cooled down in an ice bath for 10 minutes before the addition of 1% mol of catalyst. The reaction was allowed to warm up to room temperature and left stirring for 14−16 h. Polymers were isolated *via* precipitation in diethyl ether followed by filtration or decantation, then dried in a vacuum oven held at room-temperature.

PolyM1$_{100}$: Recovered as a transparent soft solid (0.75 g, 76%). GPC analysis ($CHCl_3$, 0.5% Et_3N) M_W = 64.1 kg × mol^{-1}, M_n = 22.9 kg × mol^{-1}, $Đ_M$ = 2.79.

^{1}H NMR (400 MHz, Chloroform-*d*) %*trans* = 82; *δ* 7.71 (d, *J* = 15.2 Hz, *trans* 2H), 7.12 (d, *J* = 10.2 Hz, *cis*), 5.86 (d, *J* = 10.1 Hz, *cis*), 5.74 (d, *J* = 15.1 Hz, *trans* 2H), 4.38 (t, *J* = 6.4 Hz, 4H), 2.95 (t, *J* = 6.6 Hz, 4H), 2.80 (t, *J* = 7.3 Hz, 4H), 1.84 – 1.63 (m, 4H), 1.45 (dt, *J* = 7.6, 3.7 Hz, 4H). ^{13}C NMR (101 MHz, Chloroform-*d*) δ 166.43, 165.10, 151.40, 147.92, 113.22, 112.63, 62.23, 62.17, 37.56, 36.11, 32.01, 30.22, 28.49, 28.38, 28.04

PolyM1$_{90}$M2$_{10}$: Recovered as a transparent soft solid (0.65 g, 73%). GPC analysis ($CHCl_3$, 0.5% Et_3N) M_W = 56.9 kg × mol^{-1}, M_n = 21.1 kg × mol^{-1}, $Đ_M$ = 2.69.

^{1}H NMR (400 MHz, Chloroform-*d*) %*trans* = 83; δ 7.80 – 7.53 (m, overlap M1 & M2 *trans* 2H), 7.12 (d, *J* = 10.2 Hz, overlap M1 & M2 *cis*), 5.86 (d, *J* = 10.2 Hz, overlap M1 & M2 *cis*), 5.74 (d, *J* = 15.1 Hz, overlap M1 & M2 *trans* 2H), 4.38 (t, *J* = 6.5 Hz, M1 4H), 4.11 (t, *J* = 6.7 Hz, M2), 2.95 (t, *J* = 6.6 Hz, 4H), 2.80 (t, *J* = 7.3 Hz, 4H), 1.87 – 1.65 (m, 4H), 1.45 (td, *J* = 5.7, 4.1, 2.1 Hz, 4H). ^{13}C NMR (101 MHz, Chloroform-*d*) δ 166.42, 165.54, 165.09, 151.39, 147.91, 146.86, 113.86, 113.21, 112.61, 64.34, 62.22, 62.16, 37.55, 36.10, 32.00, 30.21, 28.78, 28.48, 28.37, 28.03, 25.78.

PolyM1$_{75}$M2$_{25}$: Recovered as a transparent soft solid (0.58 g, 67%). GPC analysis ($CHCl_3$, 0.5% Et_3N) M_W = 60.2 kg × mol^{-1}, M_n = 37.6 kg × mol^{-1}, $Đ_M$ = 1.60.

^{1}H NMR (400 MHz, Chloroform-*d*) %*trans* = 84; *δ* 7.92 – 7.46 (m, overlap M1 & M2 *trans* 2H), 7.12 (d, *J* = 10.2 Hz, *cis* M2), 7.06 (d, *J* = 10.2 Hz, *cis* M1), 5.85 (dd, *J* = 10.1, 7.4 Hz, overlap M1 & M2 *cis*), 5.74 (dd, *J* = 15.1, 2.6 Hz, overlap M1 & M2 *trans* 2H), 4.39 (t, *J* = 6.5 Hz, M1 4H), 4.12 (t, *J* = 6.7 Hz, M2 1 H), 2.96 (td, *J* = 6.6, 4.4 Hz, M1 4H), 2.80 (td, *J* = 7.3, 2.2 Hz, 4H), 1.69 (q, *J* = 7.0 Hz, 8H), 1.50 – 1.33 (m, 8H). ^{13}C NMR (101 MHz, Chloroform-*d*) *δ* 165.55, 165.11, 147.92, 146.88, 113.88, 113.22, 64.35, 62.24, 37.56, 32.01, 31.96, 28.79, 28.50, 28.38, 28.05, 25.79.

PolyM1$_{50}$M2$_{50}$: Recovered as a white fibrous solid (0.83 g, 84%). GPC analysis ($CHCl_3$, 0.5% Et_3N) M_W = 76.0 kg × mol^{-1}, M_n = 30.4 kg × mol^{-1}, $Đ_M$ = 2.50.

^{1}H NMR (400 MHz, Chloroform-*d*) %trans = 84; *δ* 7.71 (d, *J* = 15.2 Hz, *trans* M1 2H), 7.67 (d, *J* = 15.2 Hz, *trans* M2 2H), 7.12 (d, *J* = 10.2 Hz, *cis* M1), 7.06 (d, *J* = 10.2 Hz, *cis* M2), 5.86 (d, *J* = 10.1 Hz, *cis* M1), 5.84 (d, *J* = 10.3 Hz, *cis* M2), 5.74 (d, *J* = 15.1 Hz, *trans* M1 2H), 5.73 (d, *J* = 15.1 Hz, *trans* M2 2H), 4.38 (t, *J* = 6.4 Hz, 4H), 4.12 (t, *J* = 6.7 Hz, 4H), 2.95 (t, *J* = 6.6 Hz, 4H), 2.80 (td, *J* = 7.3, 2.2 Hz, 8H), 1.68 (dt, *J* = 10.8, 7.2 Hz, 12H), 1.45 (p, *J* = 3.6 Hz, 8H), 1.40 (q, *J* = 3.6 Hz, 4H). ^{13}C NMR

(101 MHz, Chloroform-*d*) δ 165.55, 165.11, 147.92, 146.87, 113.87, 113.22, 64.35, 62.23, 37.56, 31.98, 30.22, 28.79, 28.52, 28.39, 28.05, 25.79.

PolyM1$_{25}$M2$_{75}$: Recovered as a white fibrous solid (0.87 g, 82%). GPC analysis ($CHCl_3$, 0.5% Et_3N) M_W = 43.8 kg × mol^{-1}, M_n = 15.7 kg × mol^{-1}, $Đ_M$ = 2.79.

^{1}H NMR (400 MHz, Chloroform-*d*) %trans = 84; *δ* 7.71 (d, *J* = 15.2 Hz, *trans* M1), 7.67 (d, *J* = 15.1 Hz, *trans* M2 2H), 7.12 (d, *J* = 10.2 Hz, *cis* M1), 7.06 (d, *J* = 10.2 Hz, *cis* M2), 5.86 (d, *J* = 10.1 *cis* M1), 5.84 (d, *J* = 10.1 Hz, *cis* M2), 5.74 (d, *J* = 15.0 Hz, *trans* M1), 5.73 (d, *J* = 15. *trans* M2, 2H), 4.39 (t, *J* = 6.5 Hz, M1), 4.12 (t, *J* = 6.7 Hz, M2 4H), 2.95 (t, *J* = 6.6 Hz, M1), 2.80 (dd, *J* = 8.4, 6.3 Hz, 4H), 1.76 – 1.62 (m, 8H), 1.45 (dq, *J* = 6.6, 3.2 Hz, 8H), 1.42 – 1.34 (m, 4H). ^{13}C NMR (101 MHz, Chloroform-*d*) *δ* 165.55, 165.11, 147.92, 146.87, 113.87, 113.22, 64.35, 62.23, 37.56, 31.96, 30.23, 28.79, 28.52, 28.39, 25.79

PolyM1$_{10}$M2$_{90}$: Recovered as a white fibrous solid (0.65 g, 84%). GPC analysis ($CHCl_3$, 0.5% Et_3N) M_W = 95.9 kg × mol^{-1}, M_n = 33.1 kg × mol^{-1}, $Đ_M$ = 2.89.

^{1}H NMR (400 MHz, Chloroform-*d*) %trans = 85; *δ* 7.71 (d, *J* = 15.2 Hz, *trans* M1), 7.67 (d, *J* = 15.2 Hz, *trans* M2 2H), 7.12 (d, *J* = 10.2 Hz, *cis* M1), 7.06 (d, *J* = 10.2 Hz, *cis* M2), 5.86 (d, *J* = 10.1 Hz, *cis* M1), 5.84 (d, *J* = 10.2 Hz, *cis* M2), 5.74 (d, *J* = 15.1 Hz, *trans* M1), 5.73 (d, *J* = 15.2 Hz, *trans* M2, 2H), 4.38 (t, *J* = 6.5 Hz, M1), 4.12 (t, *J* = 6.7 Hz, M2 4H), 2.95 (t, *J* = 6.6 Hz, M1), 2.77 (dt, *J* = 16.4, 7.3 Hz, 4H), 1.76 – 1.62 (m, 8H), 1.45 (p, *J* = 3.7 Hz, 4H), 1.40 (h, *J* = 3.6 Hz, 4H). ^{13}C NMR (101 MHz, Chloroform-*d*) *δ* 166.90, 165.55, 150.30, 146.87, 113.87, 113.27, 64.34, 36.05, 31.95, 30.23, 28.79, 28.51, 28.39, 28.06, 25.79.

PolyM2$_{100}$: Recovered as a white fibrous solid (0.88 g, 85%). GPC analysis ($CHCl_3$, 0.5% Et_3N) M_W = 102.9 kg × mol^{-1}, M_n = 28.6 kg × mol^{-1}, $Đ_M$ = 3.59.

^{1}H NMR (400 MHz, Chloroform-*d*) %*trans* = 85; *δ* 7.67 (d, *J* = 15.2 Hz, *trans* 2H), 7.06 (d, *J* = 10.2 Hz, *cis*), 5.84 (d, *J* = 10.1 Hz, *cis*), 5.73 (d, *J* = 15.1 Hz, *trans* 2H), 4.12 (t, *J* = 6.6 Hz, 4H), 2.80 (t, *J* = 7.3 Hz, 4H), 1.97 – 1.59 (m, 8H), 1.45 (ddd, *J* = 7.3, 4.7, 2.1 Hz, 4H), 1.43 – 1.33 (m, 4H). ^{13}C NMR (101 MHz, Chloroform-*d*) δ 165.55, 146.87, 113.87, 64.35, 31.95, 28.79, 28.52, 28.39, 25.79

PolyM1$_{10}$M3$_{90}$: Recovered as a transparent, tacky, soft solid (0.96 g, 84%). SEC analysis ($CHCl_3$, 0.5% Et_3N) M_W = 136.0 kg × mol^{-1}, M_n = 73.9 kg × mol^{-1}, $Đ_M$ = 1.84.

^{1}H NMR (400 MHz, Chloroform-*d*) %*trans* = 87; δ 7.70 (d, *J* = 15.1 Hz, *trans* 2H M1+M3), 7.10 (d, *J* = 10.2 Hz, *cis* M1+M3), 5.89 (d, *J* = 10.1 Hz, *cis* M1+M3), 5.77 (d, *J* = 15.2 Hz, *trans* 2H, M1+M3), 4.38 (t, *J* = 6.5 Hz, M1), 4.47 – 4.11 (m, 4H, M3), 3.88 – 3.45 (m, 4H, M3), 2.95 (t, *J* = 6.6 Hz, M1), 2.80 (t, *J* = 7.3 Hz, 4H), 2.00 – 1.63 (m, 4H), 1.45 (s, 4H). ^{13}C NMR (101 MHz, Chloroform-*d*) δ 165.22, 147.46, 113.29, 112.71, 69.24, 63.28, 31.84, 28.36, 28.27.

PolyM1$_{10}$M4$_{90}$: Recovered as a white solid (0.55 g, 84%). SEC analysis ($CHCl_3$, 0.5% Et_3N) M_W = 42.0 kg × mol^{-1}, M_n = 18.0 kg × mol^{-1}, $Đ_M$ = 2.33.

^{1}H NMR (400 MHz, Chloroform-*d*) %*trans* = 83; δ 7.72 (d, *J* = 15.1 Hz, *trans* 2H, M1+m4), 7.36 (s, 4H), 7.10 (d, *J* = 10.3 Hz, *cis*, M1+M4), 5.88 (d, *J* = 10.2 Hz, *cis*, M1+M4), 5.81 – 5.72 (m, *trans* 2H, M1+M4), 5.17 (d, *J* = 6.7 Hz, 4H), 4.38 (t, *J* = 6.5 Hz, M1), 2.95 (t, *J* = 6.5 Hz, M1), 2.77 (q, *J* = 8.3, 7.8 Hz, 4H), 1.68 (dq, *J* = 11.0, 6.8 Hz, 4H), 1.43 (p, *J* = 3.5 Hz, 4H). ^{13}C NMR (101 MHz, Chloroform-*d*) δ 165.19, 151.18, 147.72, 136.35, 128.57, 113.39, 65.85, 31.97, 28.48, 28.36.

Procedure for the synthesis of *cis*-polyM1$_{10}$M2$_{90}$

1,6-Hexanedithiol (0.25 g, 1.7 mmol, 1 eq.) was weighed in a 20 mL scintillation vial. Monomer M1 (0.04 g, 0.2 mmol, 0.1 eq.) and monomer M2 (0.33 g, 1.5 mmol, 0.9

eq.) were weighed in different vial. Monomers were then transferred to the vial containing the dithiol using DMSO. The final concentration of the thiol was kept at 0.5 M. 1% mol of Et_3N was added to the reaction mixture that was left stirring for 14−16 h. The polymer was isolated *via* precipitation in diethyl ether followed by decantation and filtration, then dried in a vacuum oven held at room temperature.

***Cis*-PolyM1$_{10}$M2$_{90}$:** Recovered as a colorless, transparent, soft, tacky solid (0.55 g, 89%). SEC analysis ($CHCl_3$, 0.5% Et_3N) M_W = 56.0 kg × mol^{-1}, M_n = 11.5 kg × mol^{-1}, $Đ_M$ = 4.87.

^{1}H NMR (400 MHz, Chloroform-*d*) %*cis* = 64; δ 7.71 – 7.60 (m, *trans*, M1+M2), 7.06 (ddd, *J* = 10.3, 3.0, 1.6 Hz, *cis*, M1+M2), 5.84 (dd, *J* = 10.1, 1.0 Hz, M1+M2), 5.73 (dd, *J* = 15.1, 1.8 Hz, *trans*, M1+M2), 4.34 (td, *J* = 7.0, 6.2, 3.2 Hz, M1), 4.17 – 4.07 (m, 4H, M2), 3.01 (t, *J* = 7.0 Hz, M1), 2.78 (dtd, *J* = 16.4, 7.4, 2.2 Hz, 4H M1), 1.70 – 1.64 (m, 4H), 1.50 – 1.36 (m, 4H).

Small molecule study to assess the impact of disulfide bond exchange

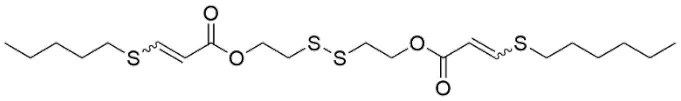

1-Hexanethiol (0.11 g, 0.97 mmol) was weighed into a 20 mL scintillation vial. Monomer M1 was added (0.12 g, 0.47 mmol)) and diluted with chloroform to reach a final concentration of thiol of 0.5 M. After cooling for ten minutes in an ice bath, 1% mol of catalyst (Et_3N) was added to the reaction mixture. The reaction was left warm up to room temperature and stirred overnight. Starting material consumption was evaluated by ^{1}H NMR spectroscopy. The disappearance of the alkyne proton signal (2.9 ppm) was used to determine the end of the reaction. The solution was concentrated under reduced pressure and the crude product obtained as a colorless fluid (0.23 g, >99% yield) was analysed *via* ^{1}H NMR, ^{13}C NMR, FTIR, and MS.

^1H NMR (400 MHz, Chloroform-*d*) %*trans* = 85 δ 7.73 (d, J = 15.1 Hz, 2H), 7.14 (d, J = 10.2 Hz, 2H), 5.85 (d, J = 10.2 Hz, 2H), 5.74 (d, J = 15.1 Hz, 2H), 4.44 – 4.29 (m, 4H), 2.95 (t, J = 6.6 Hz, 4H), 2.84 – 2.72 (m, 4H), 1.68 (tt, J = 7.5, 6.2 Hz, 4H), 1.47 – 1.33 (m, 8H), 1.38 – 1.10 (m, 6H), 1.00 – 0.74 (m, 1H). ^{13}C NMR (101 MHz, Chloroform-*d*) δ 165.0, 148.1, 112.9, 62.1, 37.5, 32.0, 31.3, 28.5, 22.5, 13.9.

HRMS (TOF-MS-EI$^+$) (m/z): $[M+H]^+$ calculated for $C_{22}H_{38}O_4S_4$, 495.1731; found, 495.1736.

Polymer cytocompatibility

Samples for cell culture studies (n = 3) were prepared by spin coating 1 mL of a solution of 0.4 wt% of polymer in $CHCl_3$ on a round glass coverslip (1 min at 1000 rpm). Polymer-coated coverslips were then sterilized by quick immersion in a 70% ethanol solution, fully dried before use, and placed into 12-well plates.

Human Epidermal Keratinocytes (HEKa) cells (C0055C, Thermofisher) were purchased from ATCC UK and cultured in T75 flasks following the supplier instructions in EpiLife cell culture media supplemented with 60 μM of calcium, human keratinocyte supplement (Thermofisher) and pen/strep (1%) at 37 °C and 5% CO_2. Cells were passaged every 3 days or until reaching 80% confluency. HEKa cells were seeded on polymer-coated glass coverslips (n = 3, 7,600 cells per well) and cultured at 37 °C, 5% CO_2. At each time point (24 h, 72 h, and 7 days), the solution was removed and cells were washed with PBS (1 mL × 3) before incubation with 10% PrestoBlue metabolic assay (Invitrogen) following the supplier instructions. Bare glass coverslips were used as control. Fluorescence was measured using a Fluostar Omega plate reader (BMG Labtech) (Ex. 590 nm, Em. 610 nm).

Cell spreading

HEKa cells were seeded on top of polymer-coated coverslips (n = 3, 22,800 cells per well) were seeded into 12-well plates and cultured at 37 °C and 5% CO_2. Media was replaced with fresh media every 2 days. After 5 days incubation the media was removed, and coverslips were rinsed with PBS and fixed with 4% paraformaldehyde for 15 minutes at room temperature. After rinsing with PBS (3 × 1 mL), cells were permeabilized through incubation with 0.5% Triton X-100 (1 mL per well) for 10 minutes at room temperature. After further PBS washing (3 × 1 mL), ActinRed 555 Ready Probes Reagent (Rhodamine Phalloidin - Cat. No. R415) was added to each well (2 drops mL^{-1} per well, following supplier instructions) and incubated in the dark for 30 minutes. After PBS washing (3 × 1 mL), 1 mL of DAPI solution (300 nM - D9542, Sigma-Aldrich) was added to each coverslip and incubated for 30 minutes, followed by PBS washing (3 × 1 mL). Samples were imaged through confocal fluorescence microscopy (Olympus FV3000RS laser confocal microscope), using the blue (405 nm) and red (454 nm) lasers. Pictures were taken at 20x magnification and processed using Olympus CellSens software and ImageJ.

NMR spectroscopy of synthesized monomers

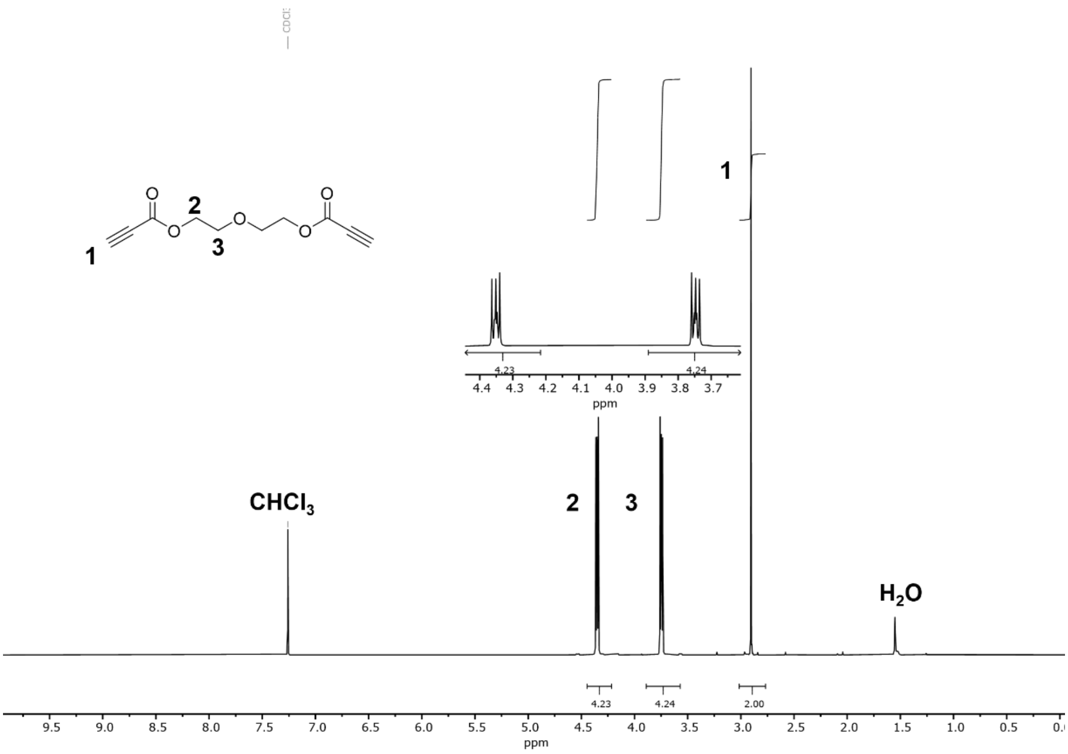

Figure S1. Monomer M3 – ^{1}H NMR spectrum (400 MHz, 298 K, $CDCl_3$).

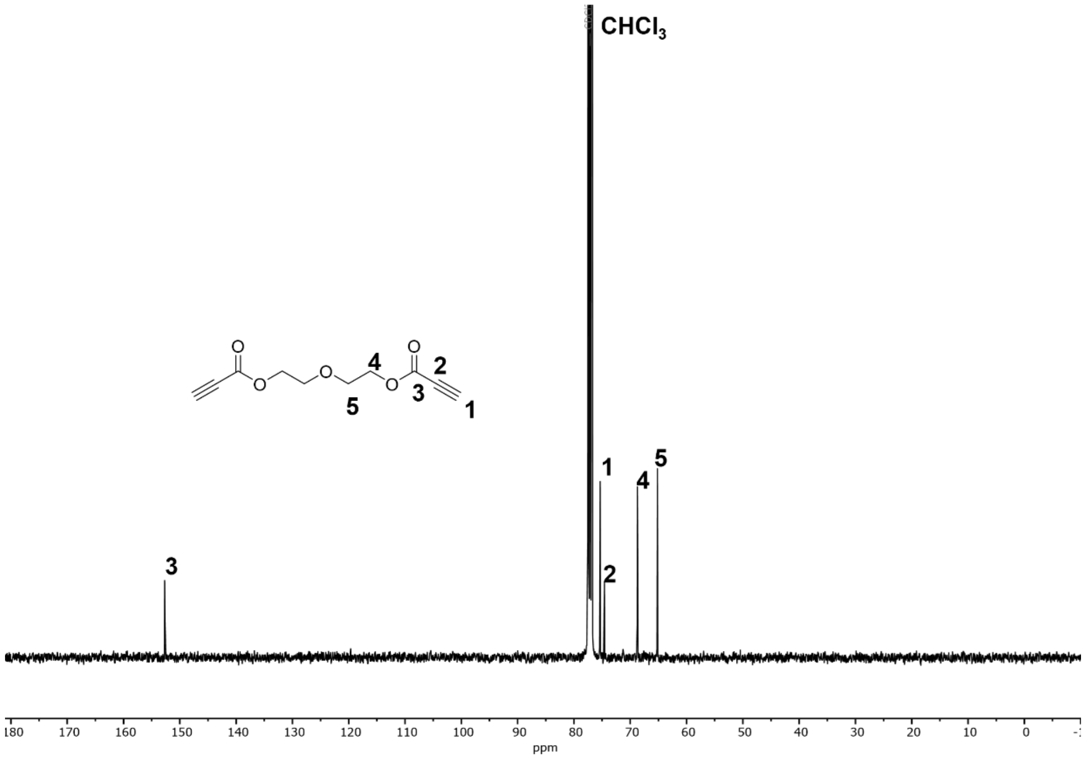

Figure S2. Monomer M3 – ^{13}C NMR spectrum (100.57 MHz, 298 K, $CDCl_3$).

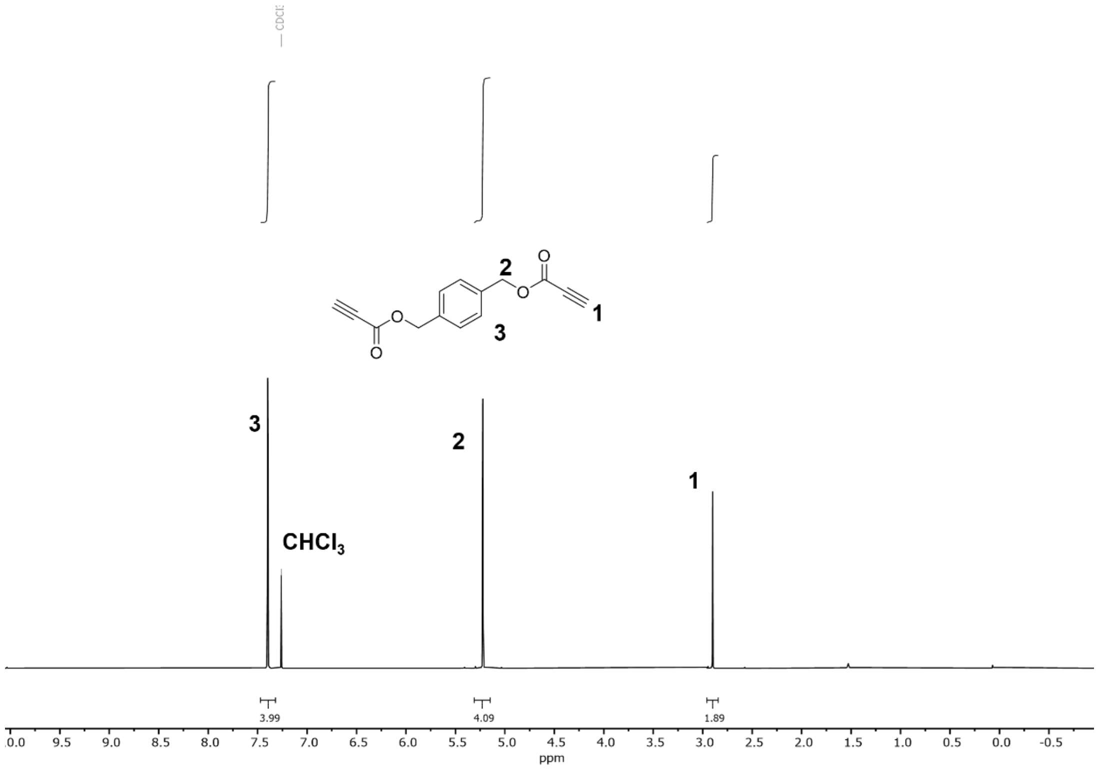

Figure S3. Monomer M4 – ^{1}H NMR spectrum (400 MHz, 298 K, $CDCl_3$).

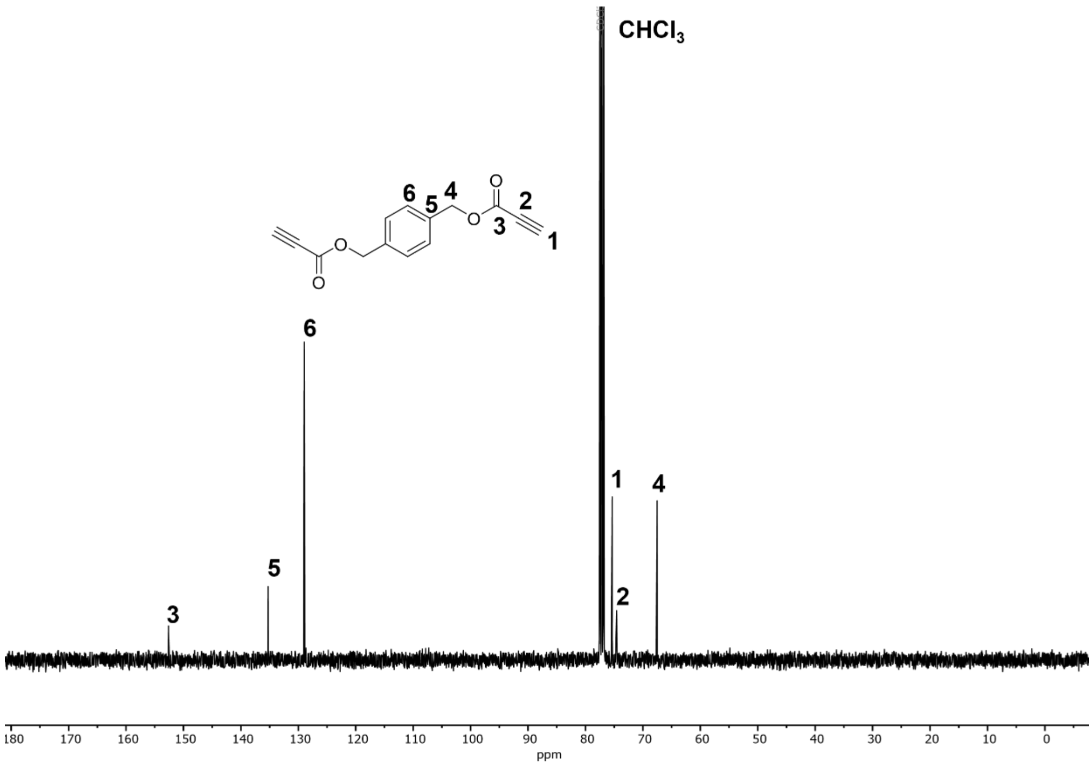

Figure S4. Monomer M4 – ^{13}C NMR spectrum (100.57 MHz, 298 K, $CDCl_3$).

Characterization data of small molecules study

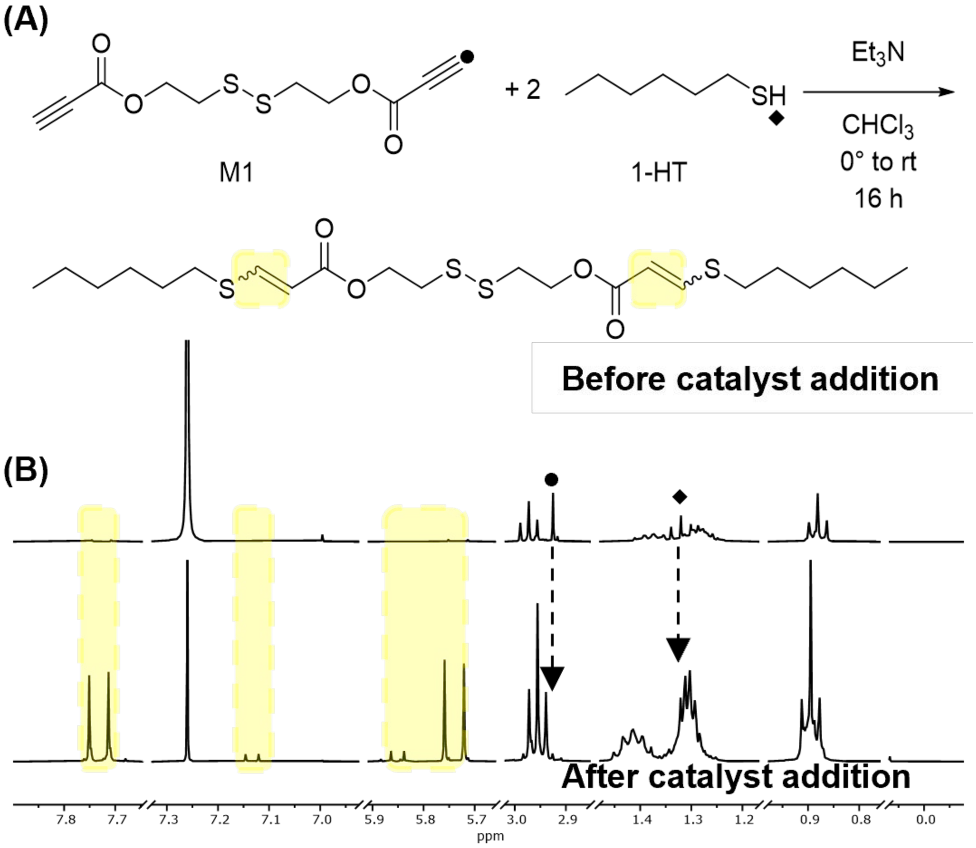

Figure S5. (A) Thiol-yne addition reaction between 2 equivalents of 1-HT and 1 equivalent of M1 in the presence of 1 mol% of Et_3N. (B) 1H NMR spectra (400 MHz, 298 K, $CDCl_3$) of reactants (1-HT and M1) and reaction mixture before and after catalyst addition. Yellow boxes and light blue dashed lines added to help visualize the change.

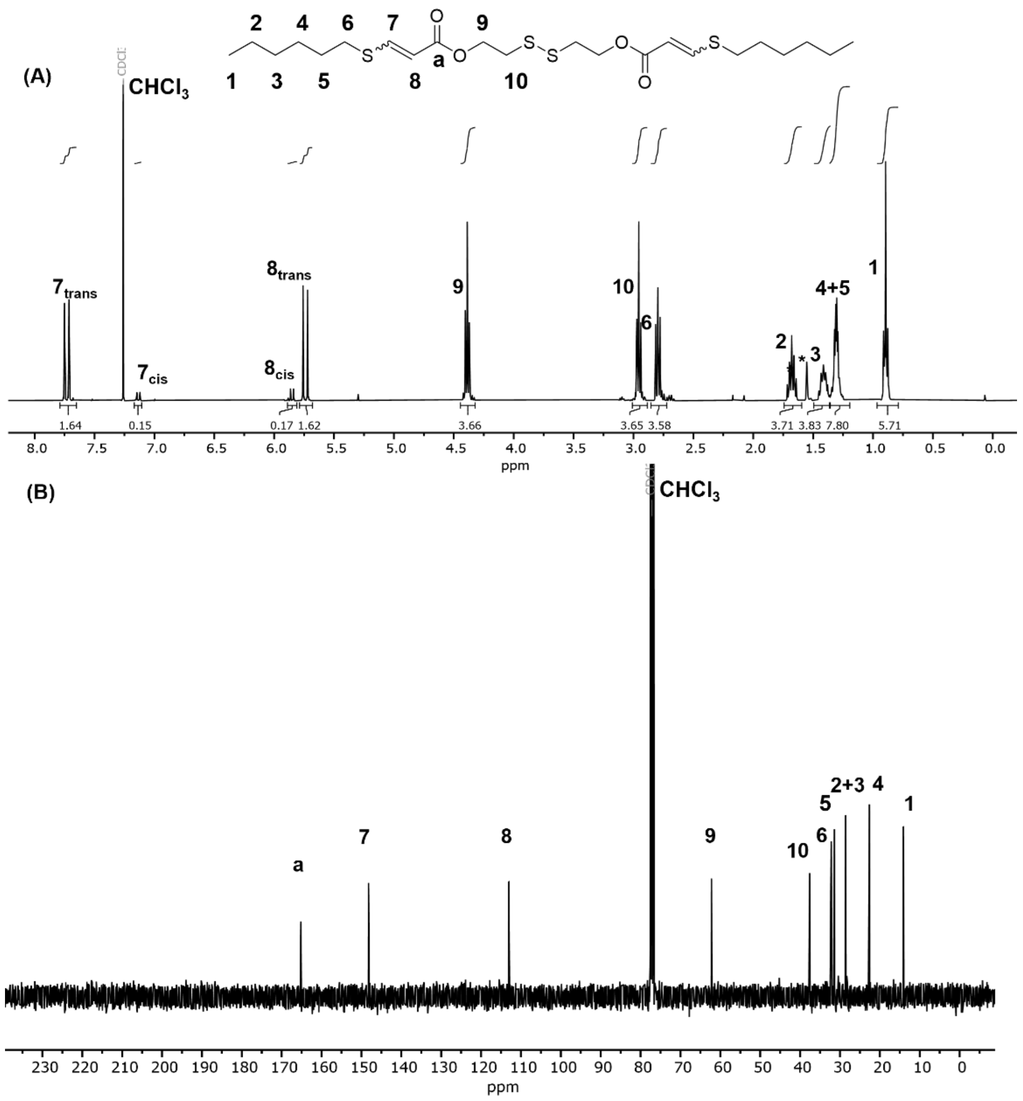

Figure S6. (A) structure of addition product and assigned ^{1}H NMR spectrum (400 MHz, 298 K, $CDCl_3$), (B) ^{13}C NMR spectrum (100.57 MHz, 298 K, $CDCl_3$) of the crude reaction mixture after drying of small molecule study. Solvent and residual water peaks are marked with an asterisk.

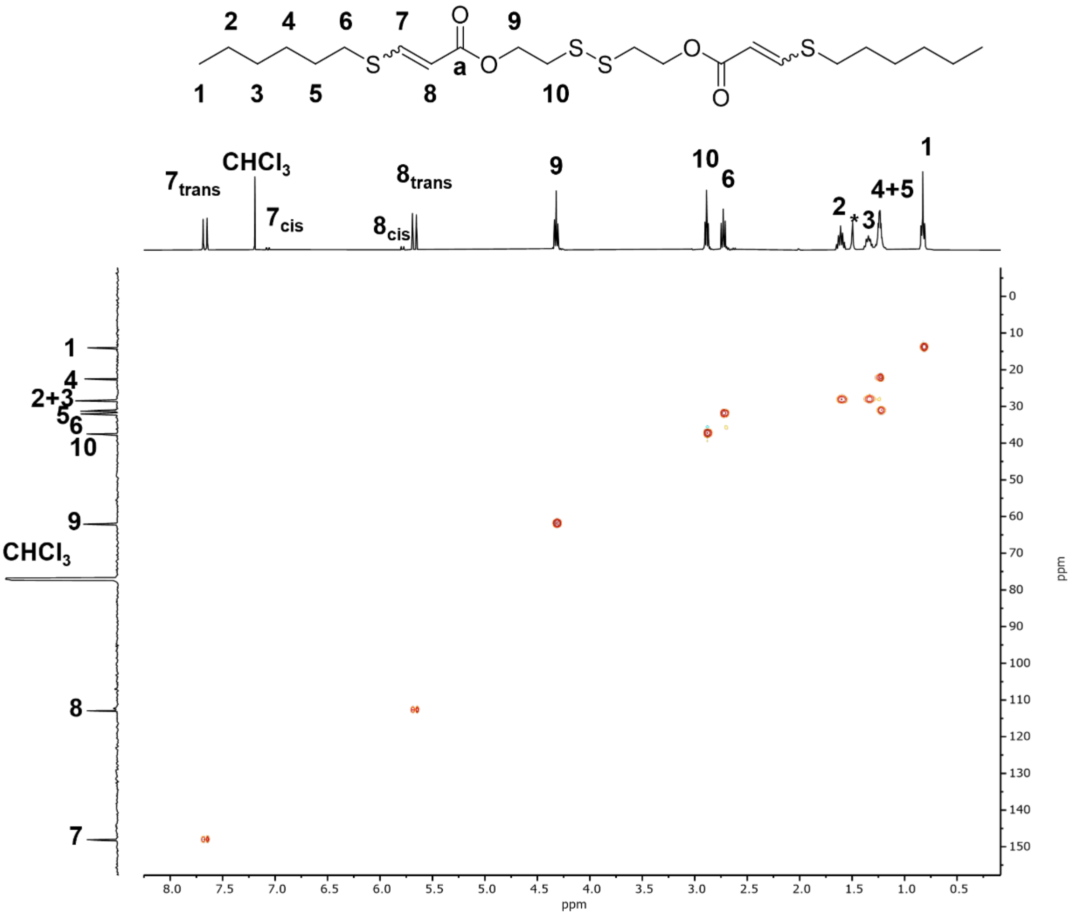

Figure S7. HSQC NMR spectrum (298 K, $CDCl_3$) of the crude reaction mixture after removal of the solvent of small molecule study. Solvent and residual water peaks are marked with an asterisk.

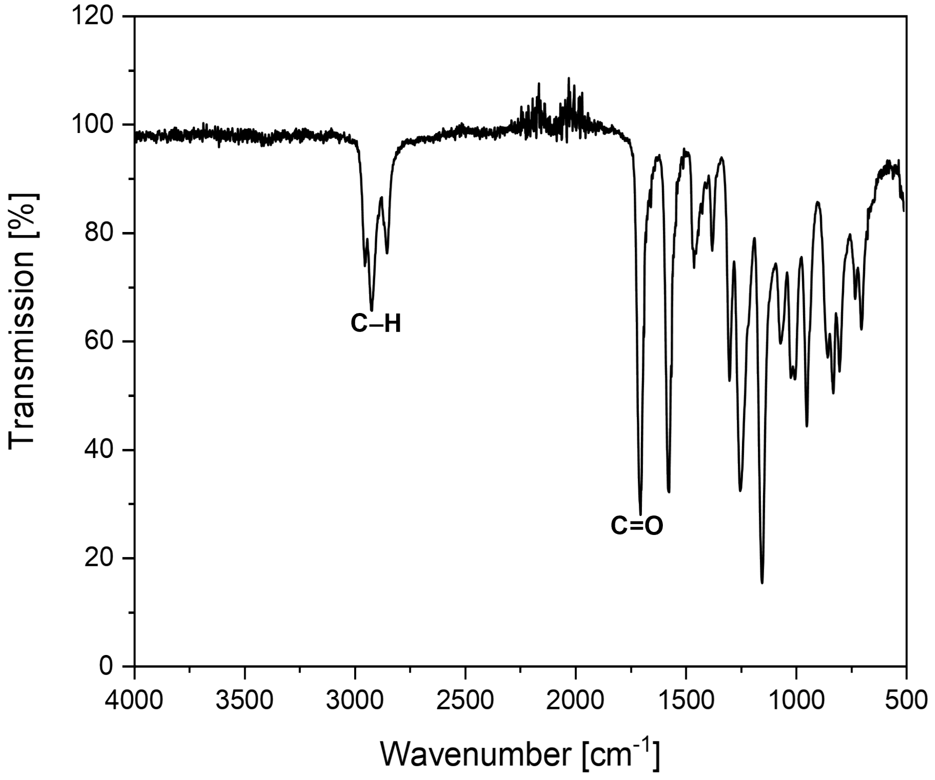

Figure S8. FTIR spectra of crude reaction mixture after solvent removal of small molecule study.

^{1}H NMR spectra of solvent/catalyst screening

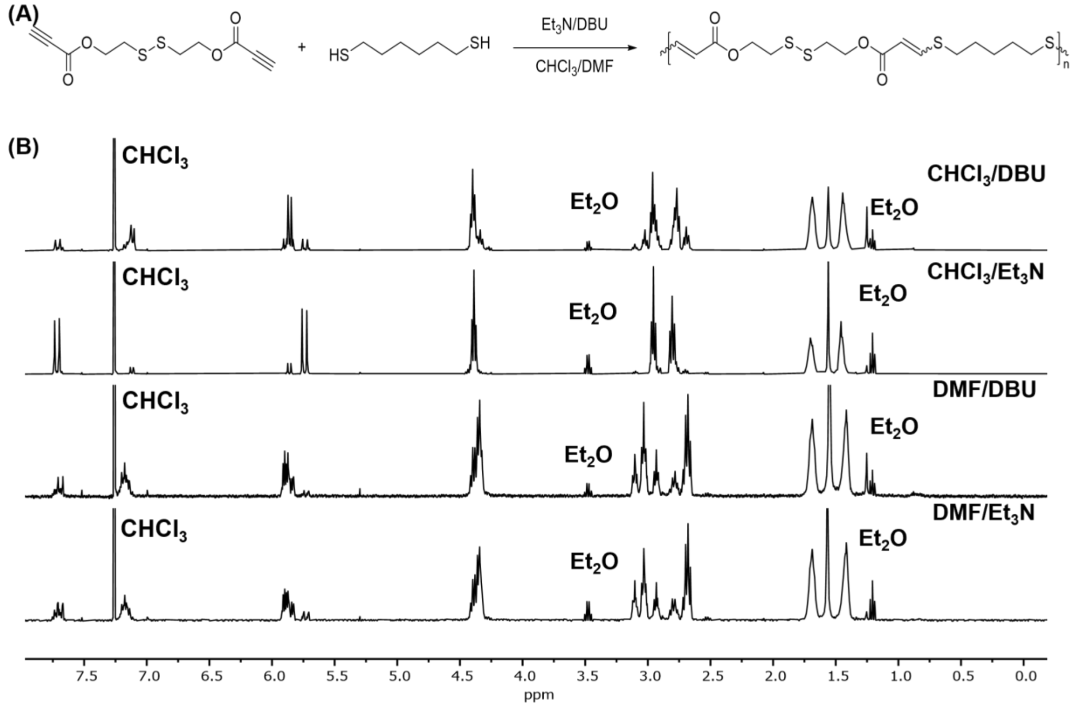

Figure S9. (A) Thiol-yne polymerization between monomer M1 and 1,6-hexanedithiol under different combinations of solvent/catalyst, with a catalyst loading of 1 mol%. (B) Stacked ^{1}H NMR spectra (400 MHz, 298 K, $CDCl_3$) of copolymers. Residual diethyl ether (Et_2O) peaks from the precipitation solvent assigned. The $CHCl_3$/Et_3N combination was selected as it gave origin to fewer signals suggesting a more controlled reaction.

SEC traces of solvent/catalyst screening

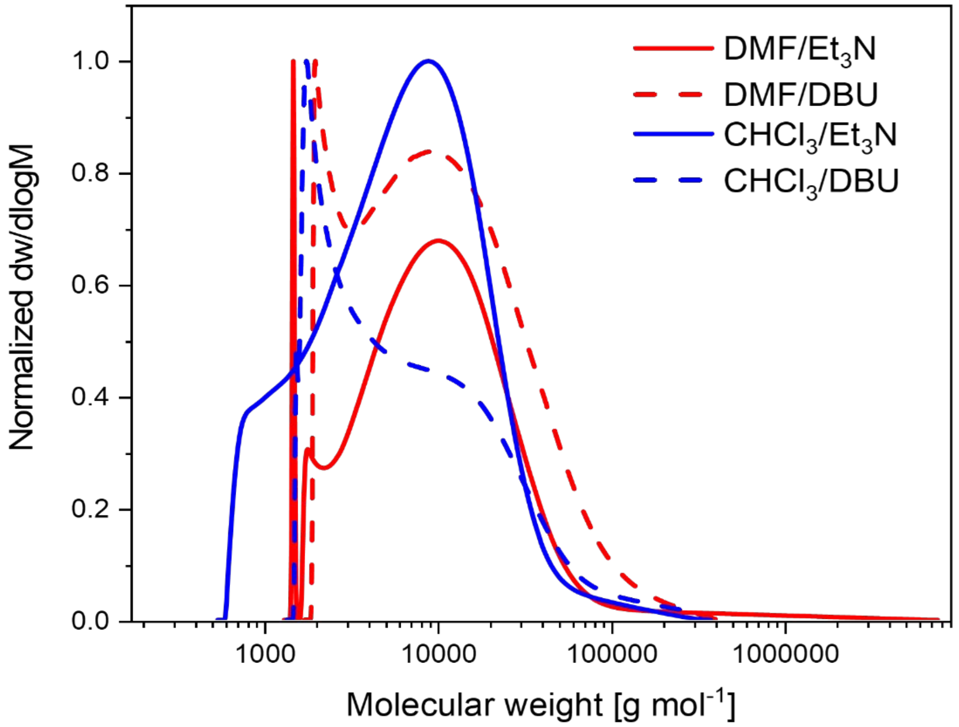

Figure S10. SEC ($CHCl_3$, 0.5 % Et_3N) traces of copolymers from different solvent/catalyst combinations.

^{1}H NMR & ^{13}C NMR spectra and SEC traces of *trans* polymers with varying amount of monomer M1

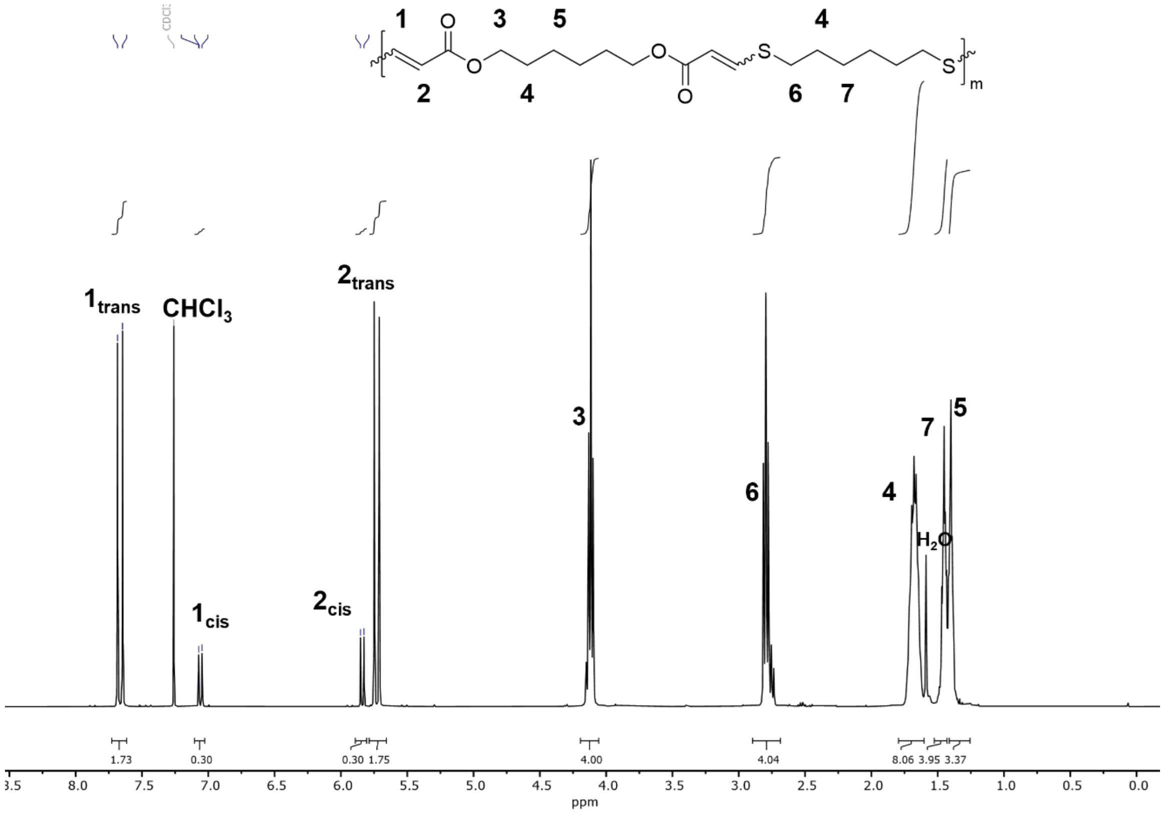

Figure S11. PolyM2$_{100}$ – ^{1}H NMR spectrum (400 MHz, 298 K, $CDCl_3$).

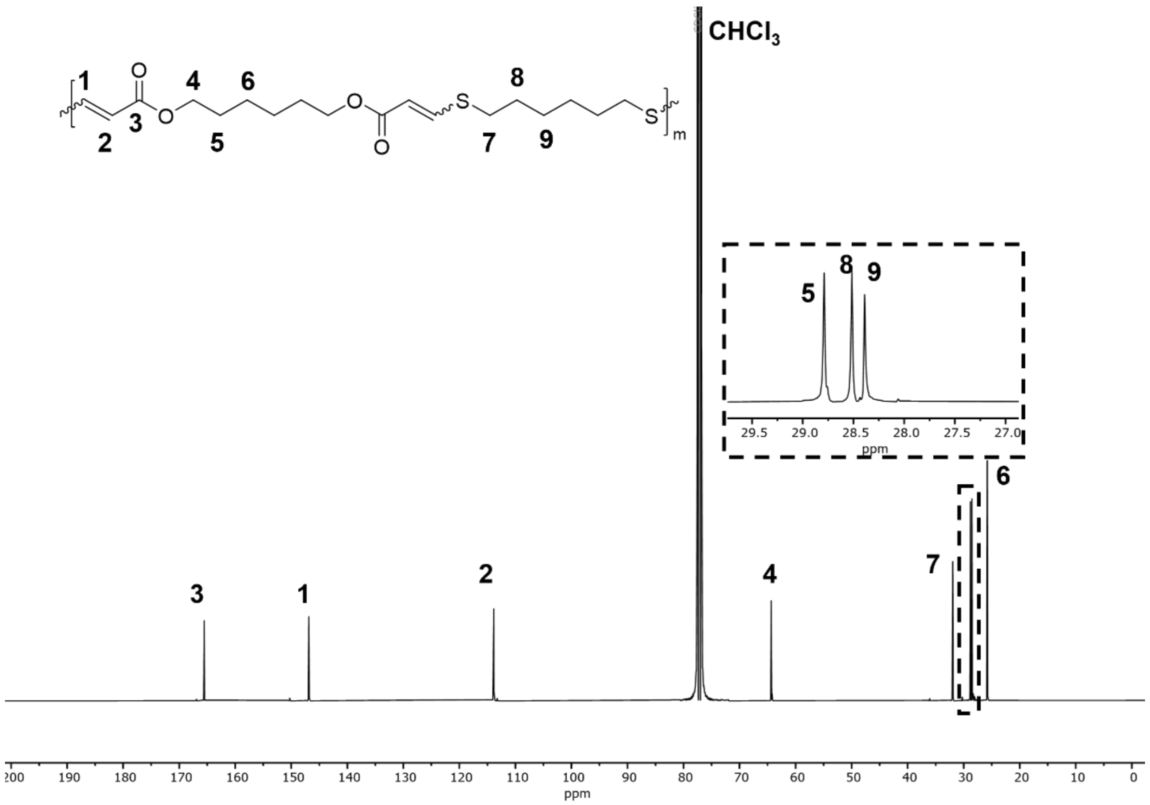

Figure S12. PolyM2$_{100}$ – ^{13}C NMR spectrum (100.57 MHz, 298 K, $CDCl_3$). Only major resonances (*trans* isomer) assigned.

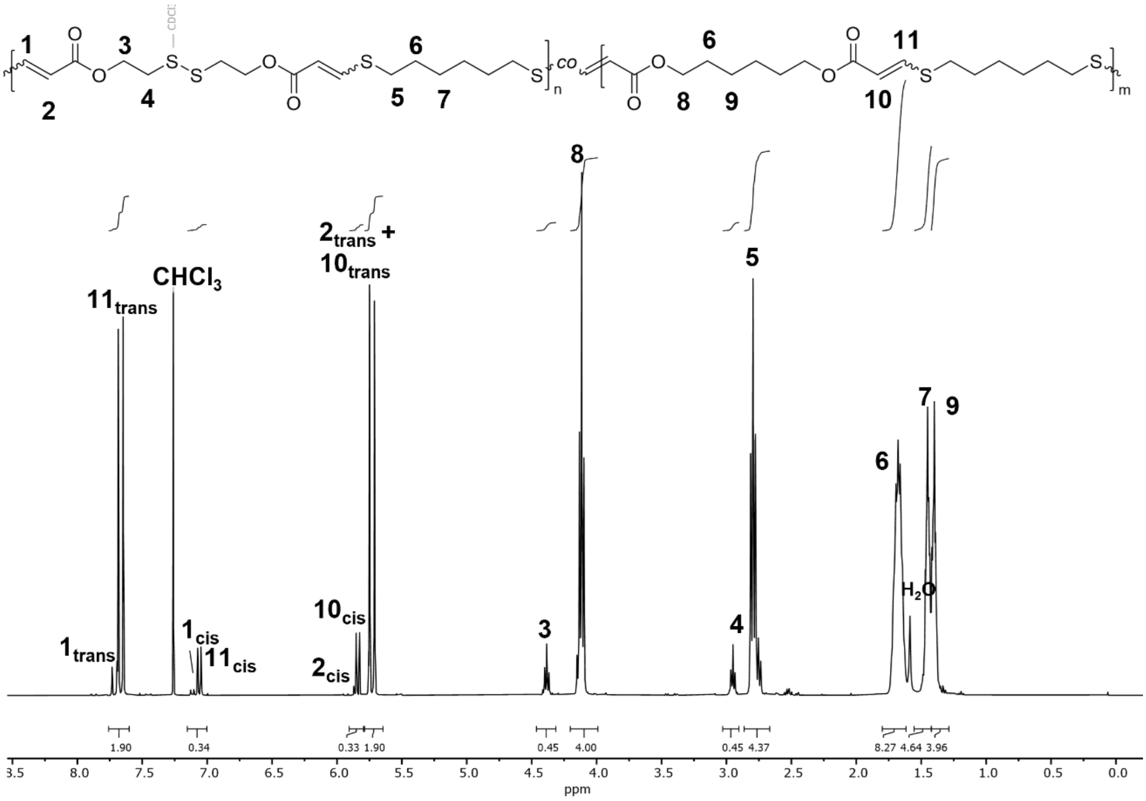

Figure S13. PolyM1$_{10}$M2$_{90}$ – ^{1}H NMR spectrum (400 MHz, 298 K, $CDCl_3$).

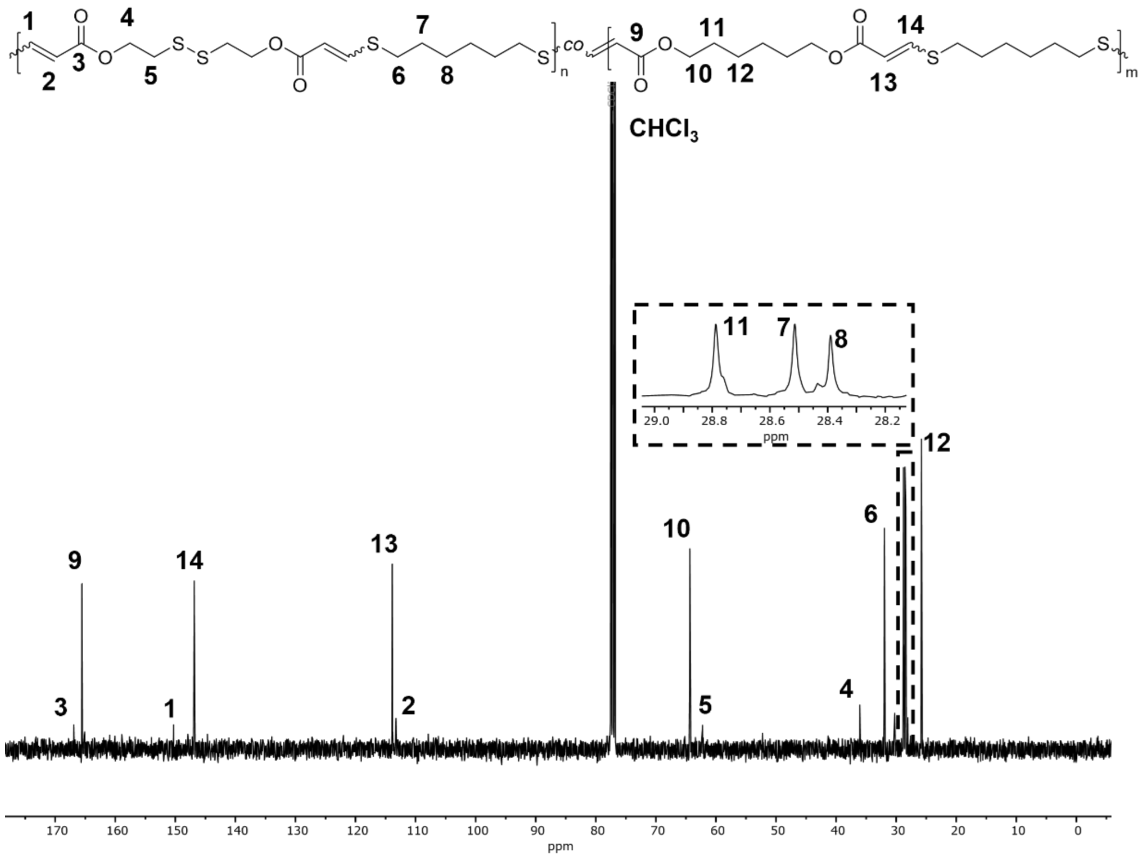

Figure S14. PolyM1$_{10}$M2$_{90}$ – ^{13}C NMR spectrum (100.57 MHz, 298 K, $CDCl_3$). Only major resonances (*trans* isomer) assigned.

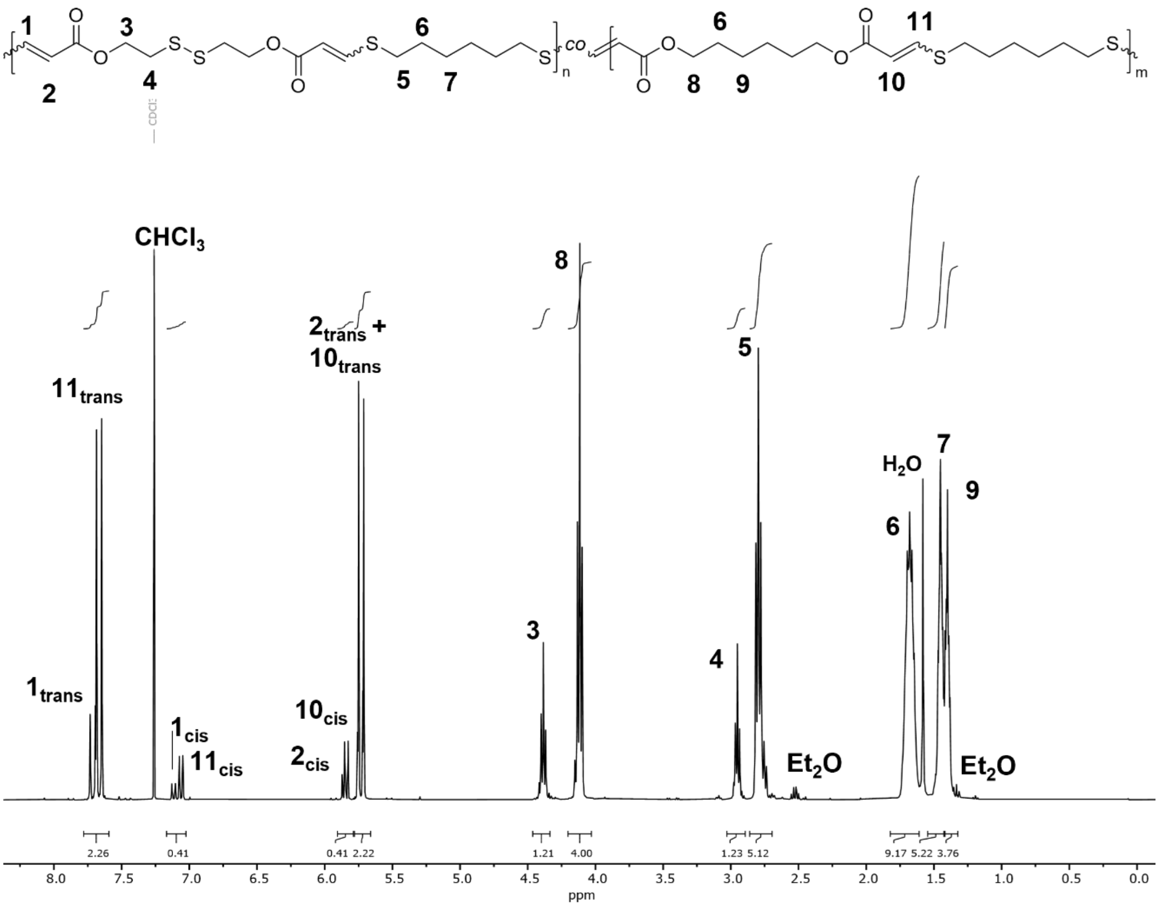

Figure S15. PolyM1$_{25}$M2$_{75}$ – ^{1}H NMR spectrum (400 MHz, 298 K, $CDCl_3$).

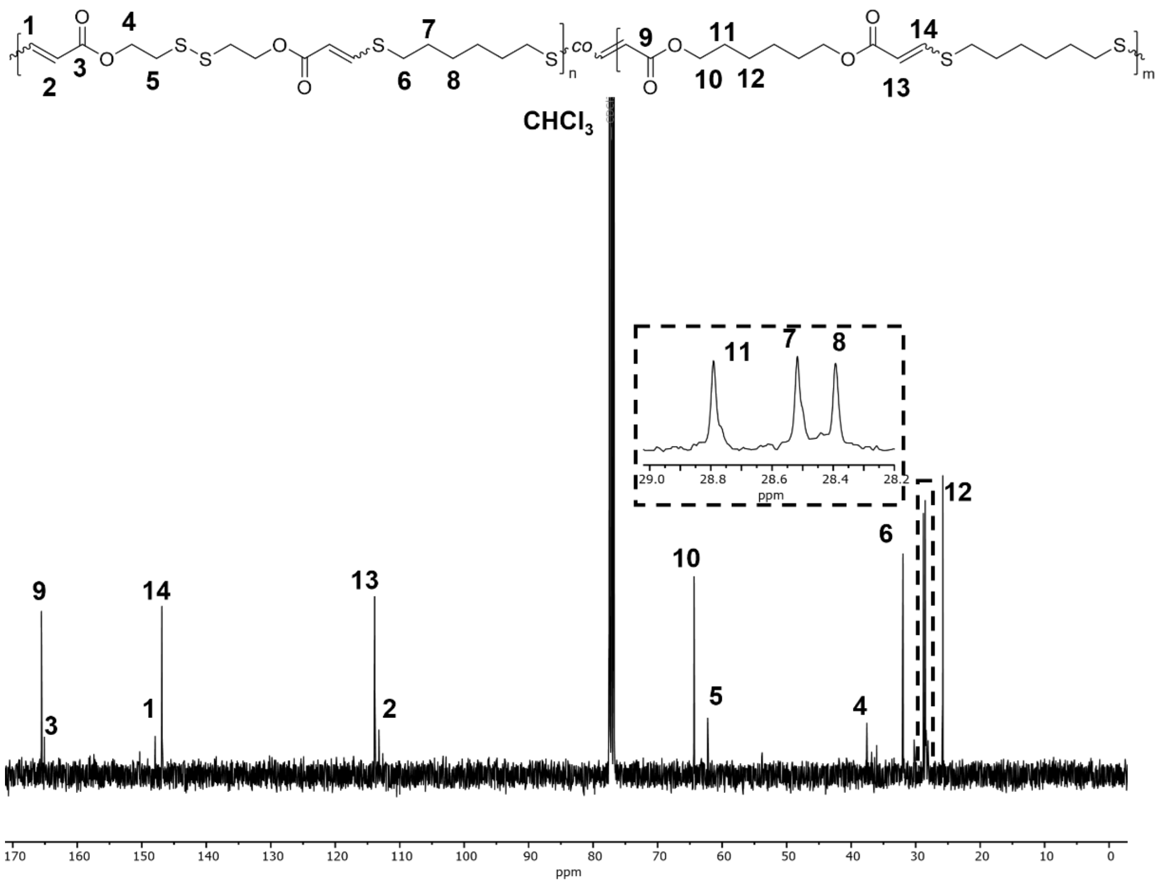

Figure S16. PolyM1$_{25}$M2$_{75}$ – ^{13}C NMR spectrum (100.57 MHz, 298 K, $CDCl_3$). Only major resonances (*trans* isomer) assigned.

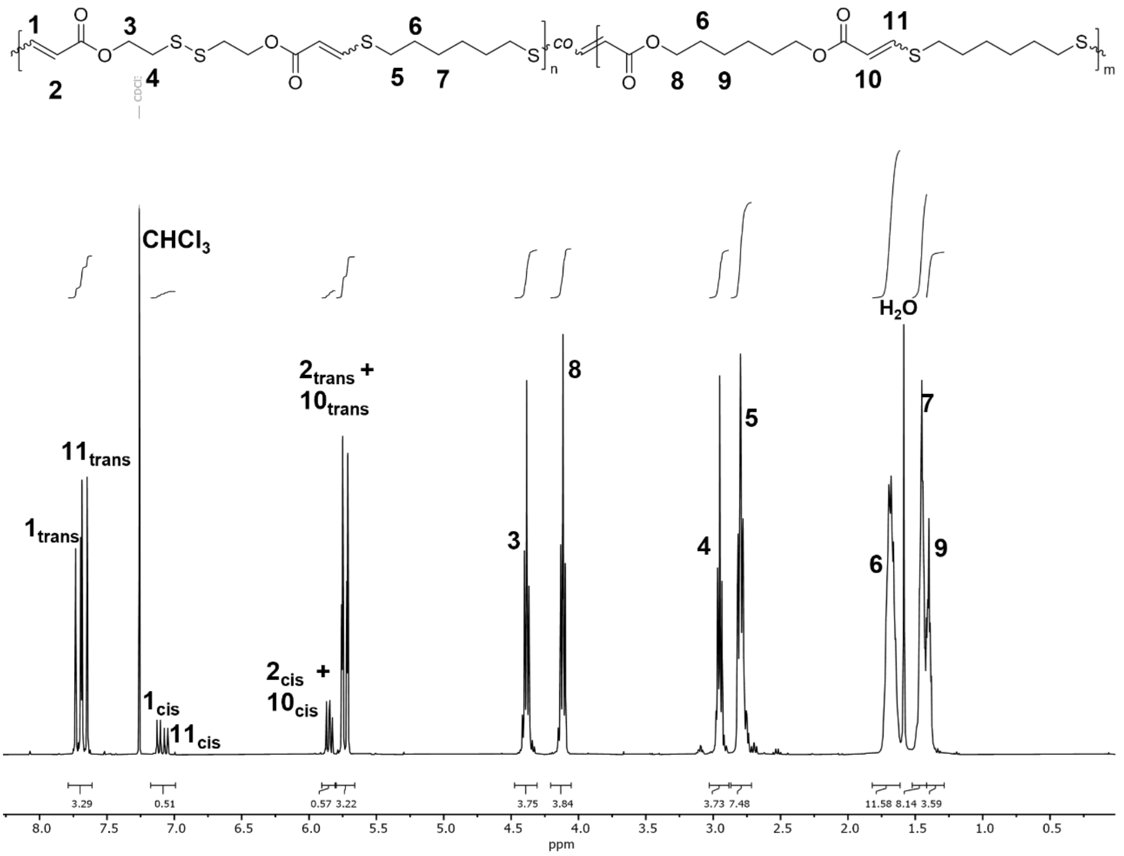

Figure S17. PolyM1$_{50}$M2$_{50}$ – ^{1}H NMR spectrum (400 MHz, 298 K, CDCl$_3$).

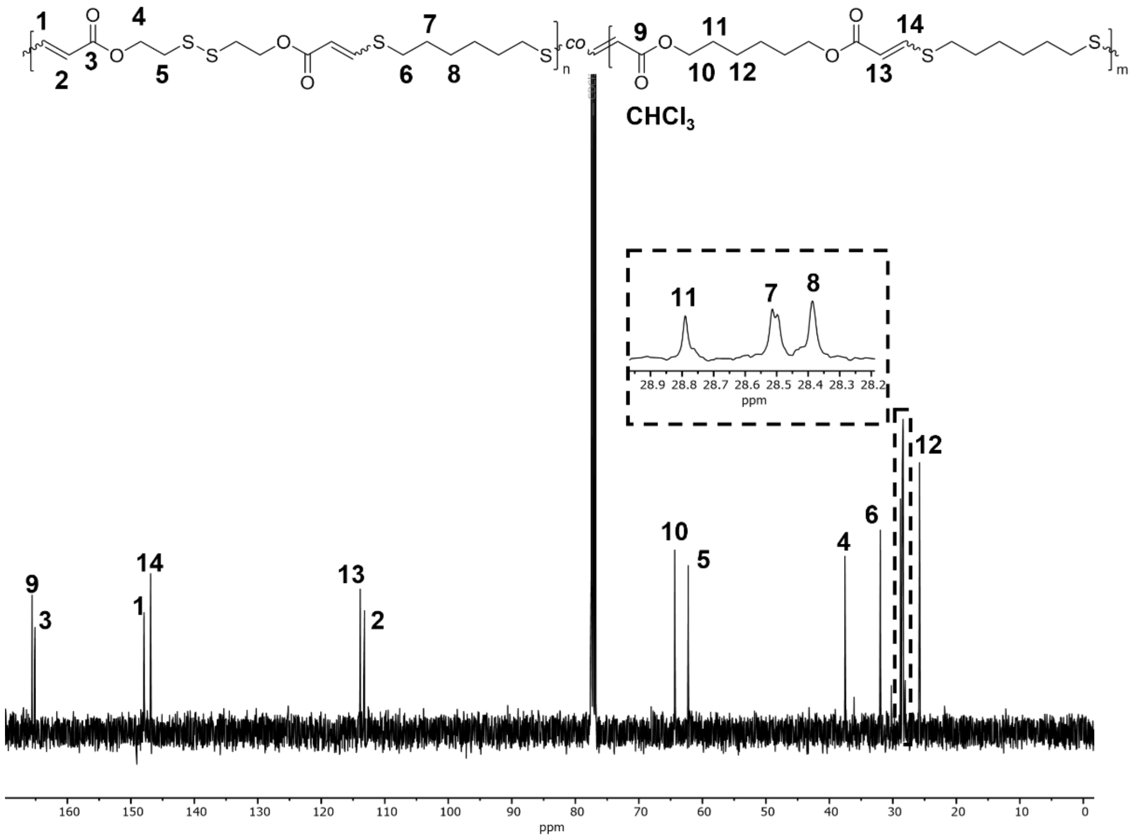

Figure S18. PolyM1$_{50}$M2$_{50}$ – ^{13}C NMR spectrum (100.57 MHz, 298 K, $CDCl_3$). Only major resonances (*trans* isomer) assigned.

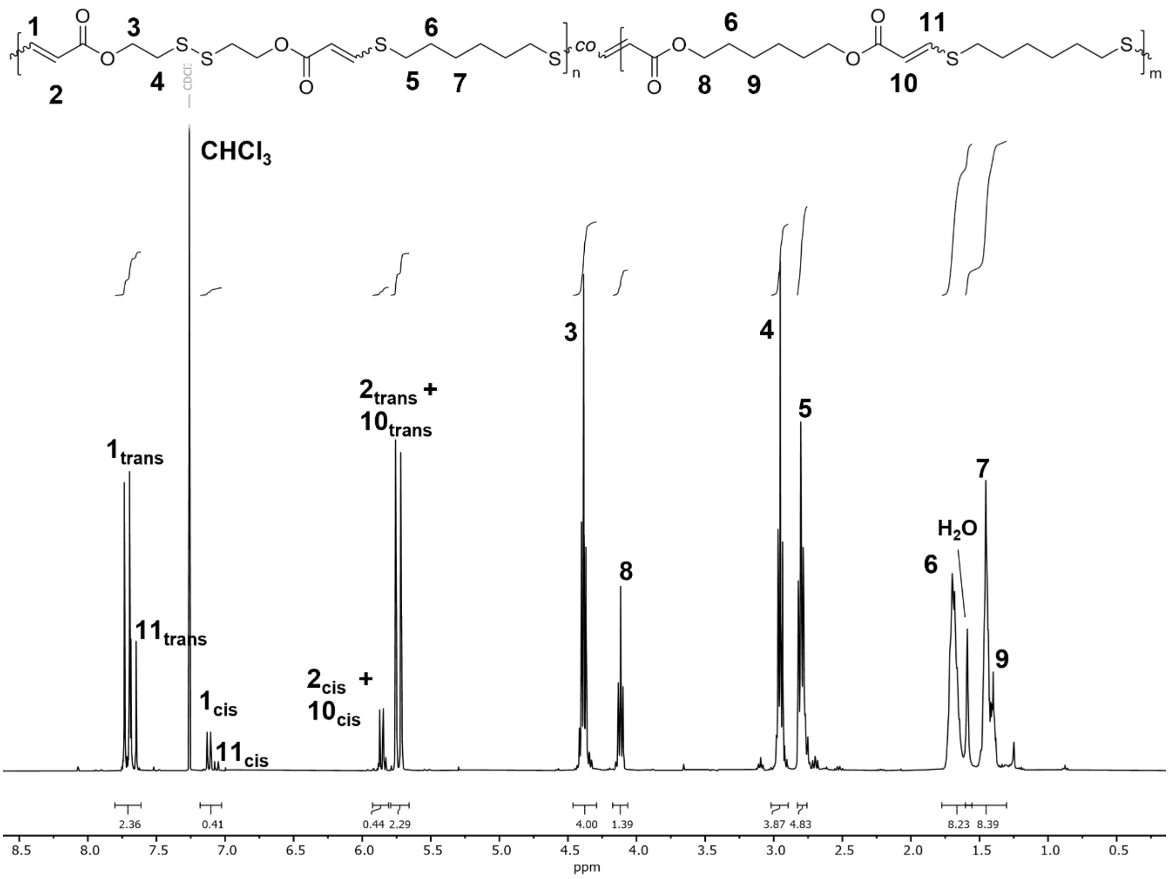

Figure S19. PolyM1$_{75}$M2$_{25}$ – ^{1}H NMR spectrum (400 MHz, 298 K, CDCl$_3$).

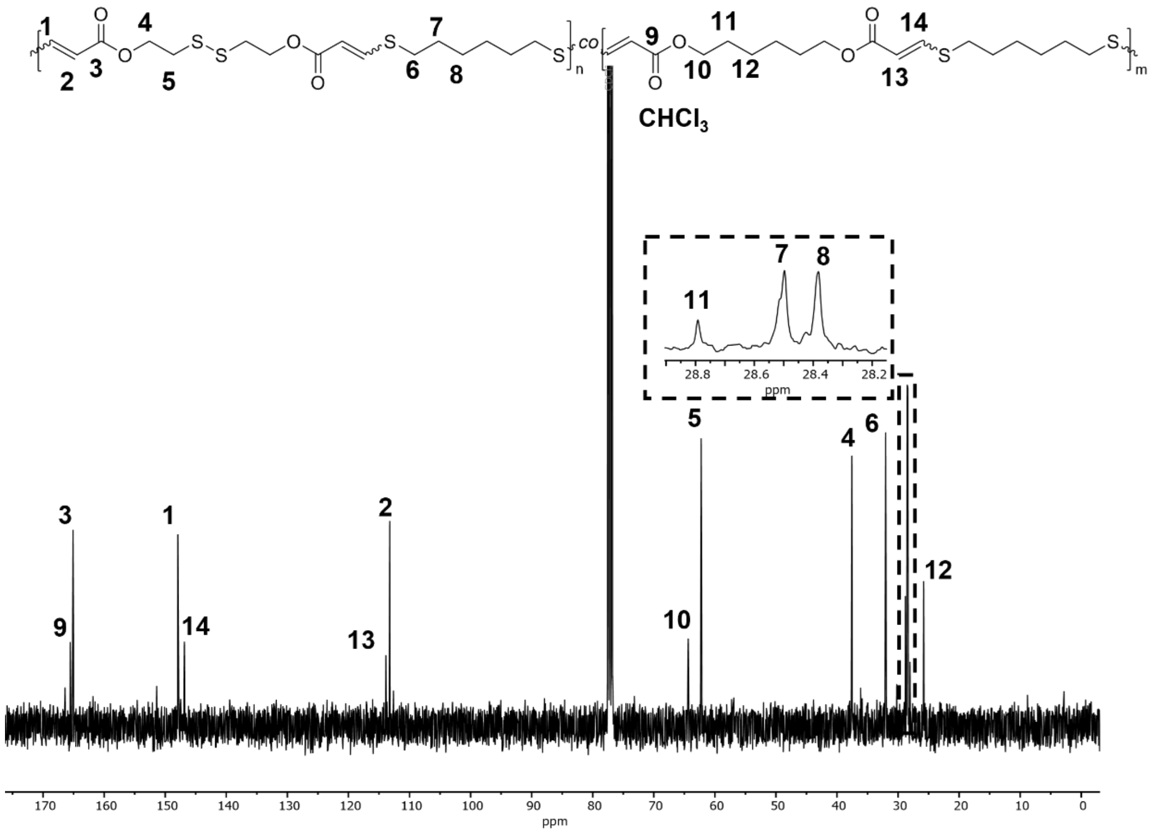

Figure S20. PolyM1$_{75}$M2$_{25}$ – ^{13}C NMR spectrum (100.57 MHz, 298 K, $CDCl_3$). Only major resonances (*trans* isomer) assigned.

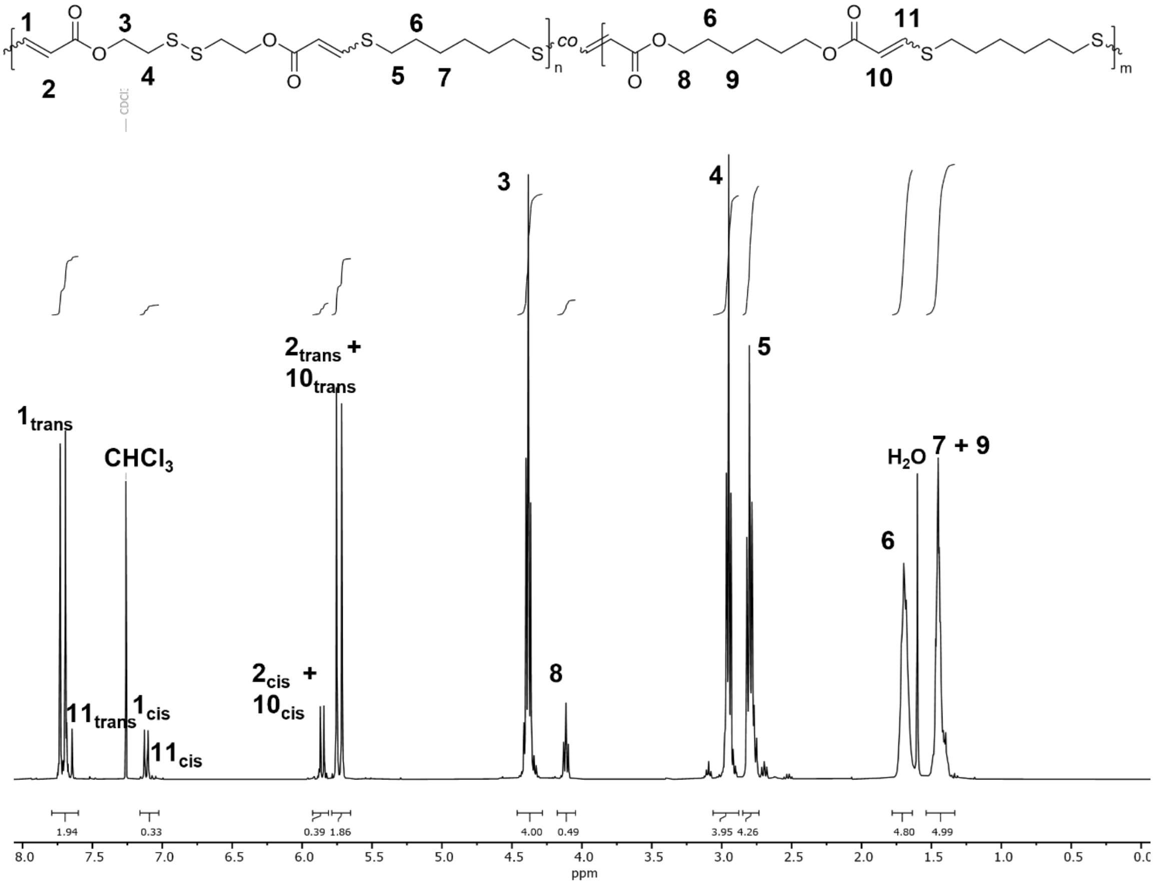

Figure S21. PolyM1$_{90}$M2$_{10}$ – ^{1}H NMR spectrum (400 MHz, 298 K, $CDCl_3$).

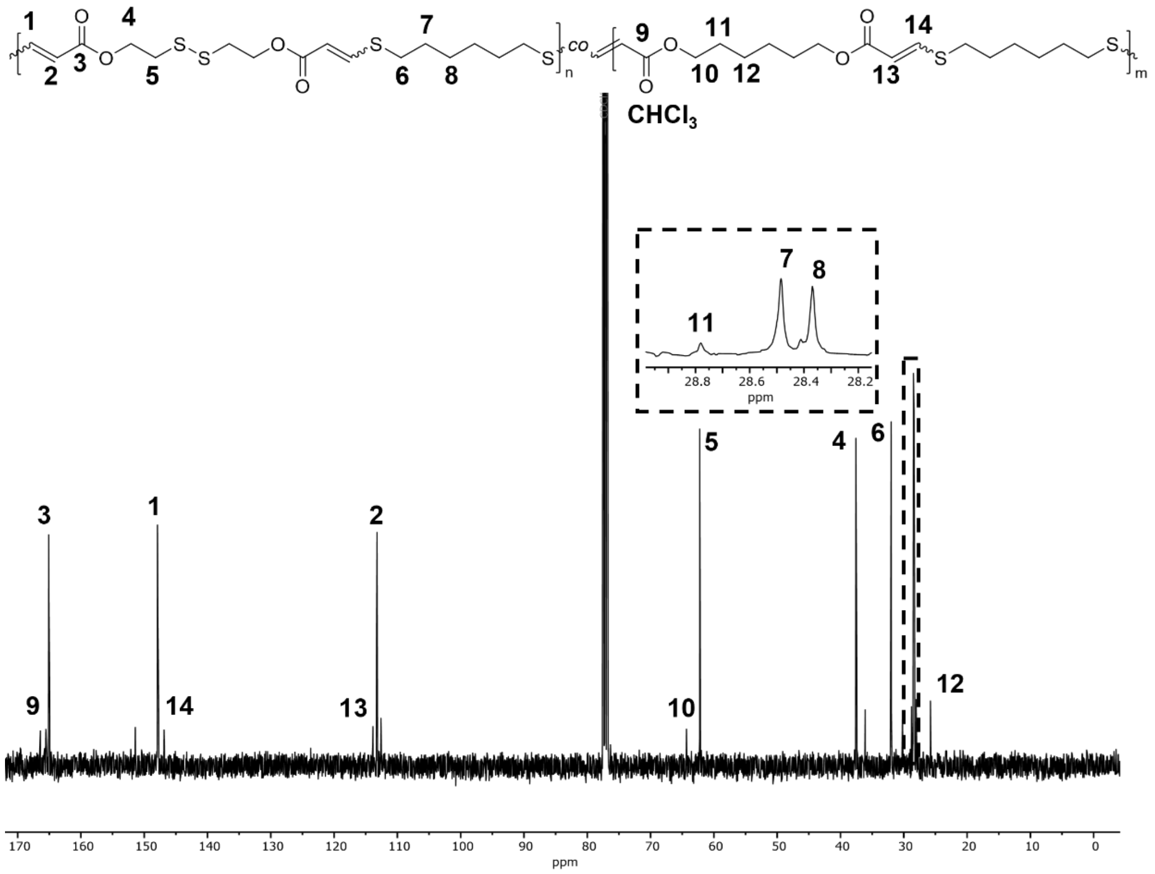

Figure S22. PolyM1$_{90}$M2$_{10}$ – ^{13}C NMR spectrum (100.57 MHz, 298 K, $CDCl_3$). Only major resonances (*trans* isomer) assigned.

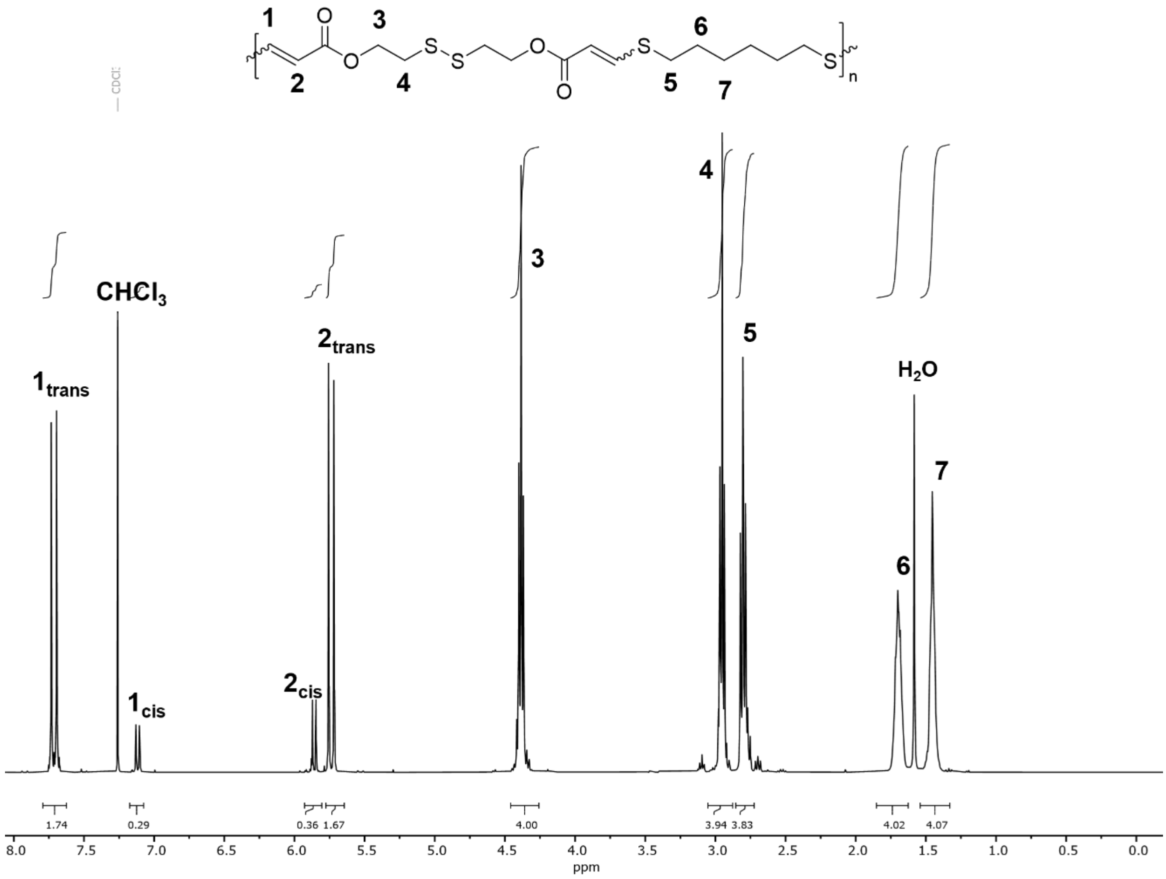

Figure S23. PolyM1$_{100}$ – ^{1}H NMR spectrum (400 MHz, 298 K, $CDCl_3$).

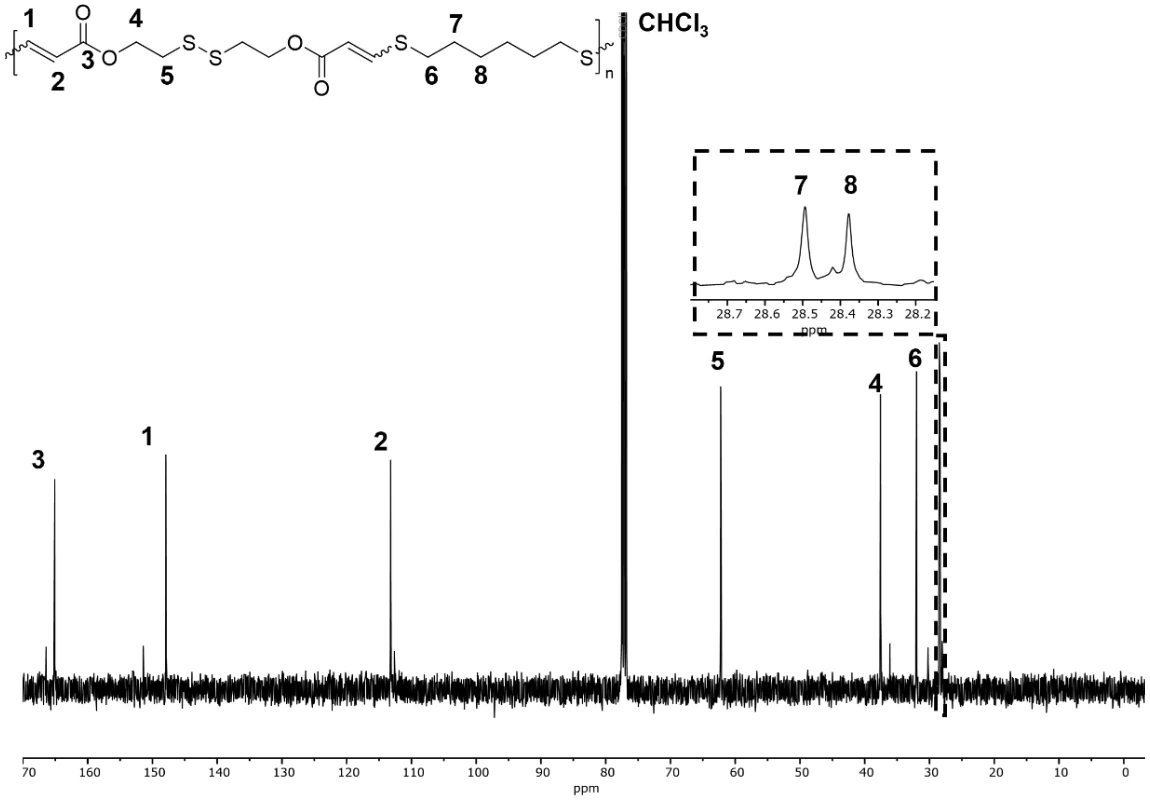

Figure S24. PolyM1$_{100}$ – ^{13}C NMR spectrum (100.57 MHz, 298 K, CDCl$_3$). Only major resonances (*trans* isomer) assigned.

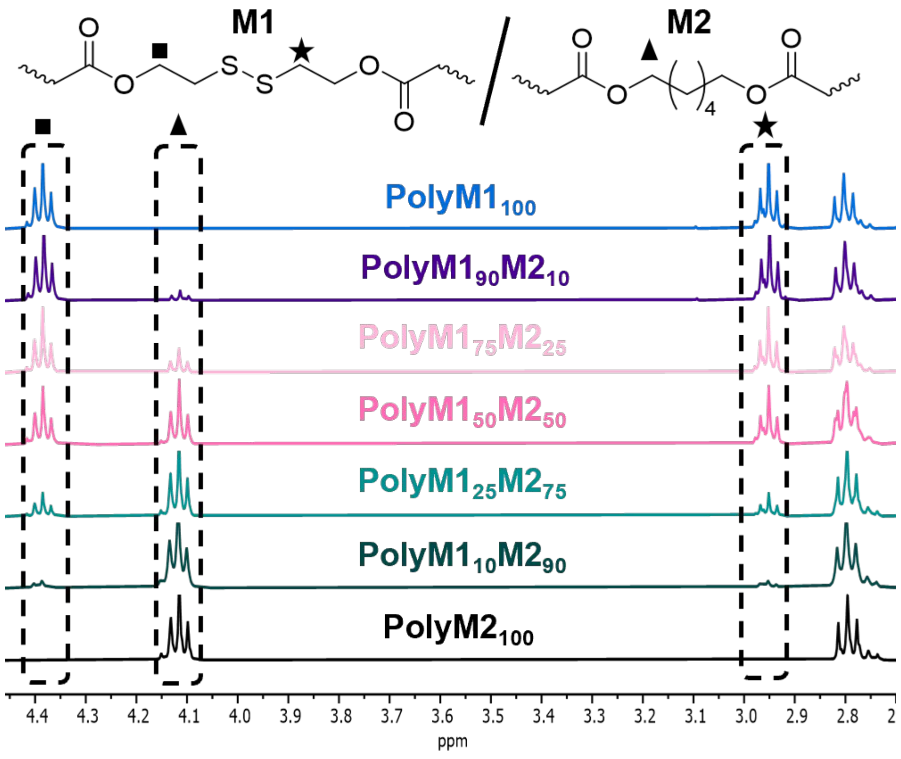

Figure S25. Zoomed-in ^{1}H NMR spectra (400 MHz, 298 K, $CDCl_3$) of polymers synthesized varying the M1/M2 ratio going from 0 equivalents (bottom) of M1 to 1 equivalent (top) expressed to the equivalent of thiol. The resonances at 4.4 ppm, 4.1 ppm and 2.95 ppm follow the monomers feeding ratio.

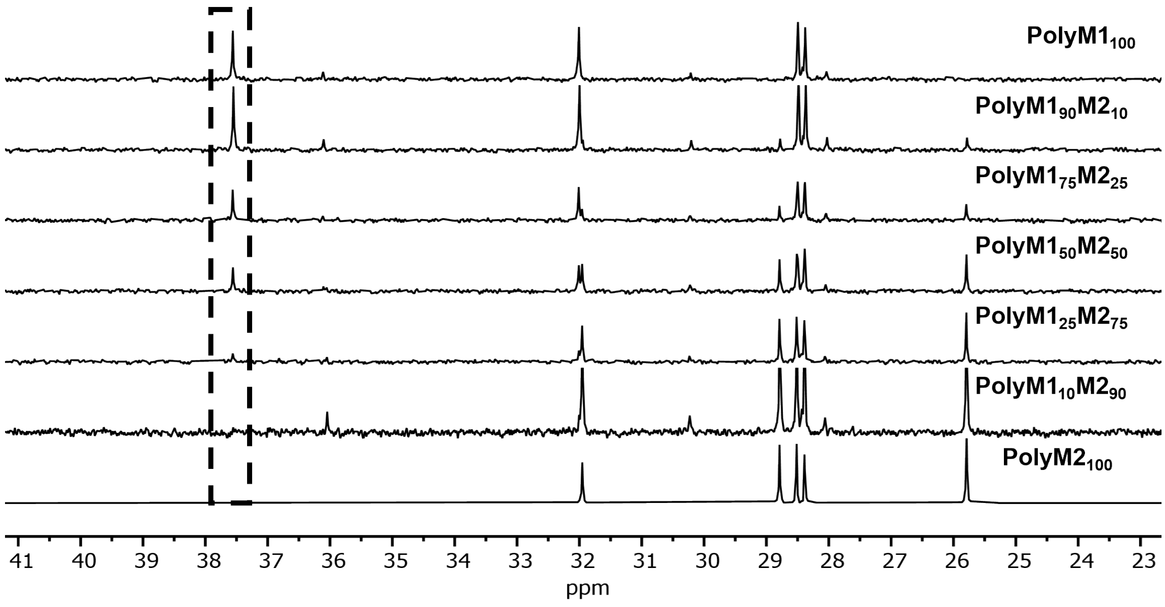

Figure S26. stacked ^{13}C NMR spectra (400 MHz, 298 K, $CDCl_3$) of copolymer obtained reacting 1,6-hexanedithiol and different ratios of monomer 1 and 2 respectively. Characteristic S-S $^{\beta}$C signal (ppm>35) intensity increases with increasing monomer M1 content.

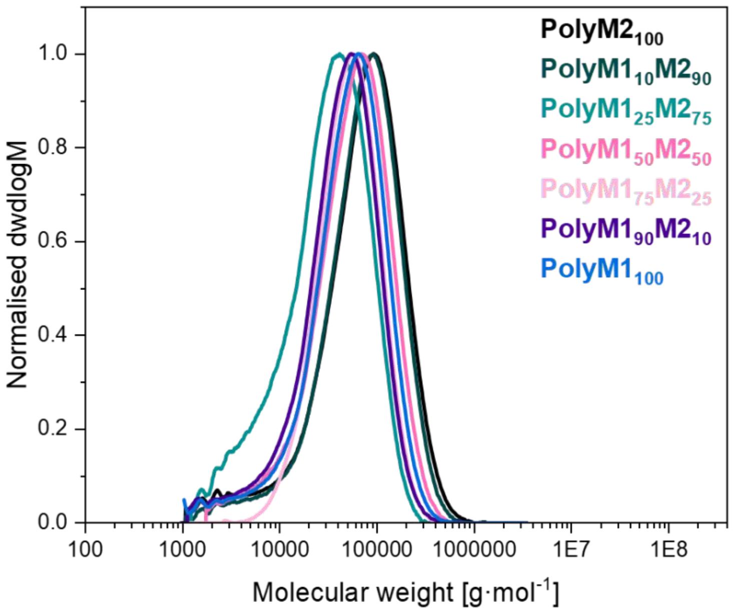

Figure S27. SEC ($CHCl_3$, Et_3N 0.5 % against polystyrene standards) traces of polymers having different degrees of M1 in the backbone.

Thermal characterization of *trans* polymer of different monomer M1 content

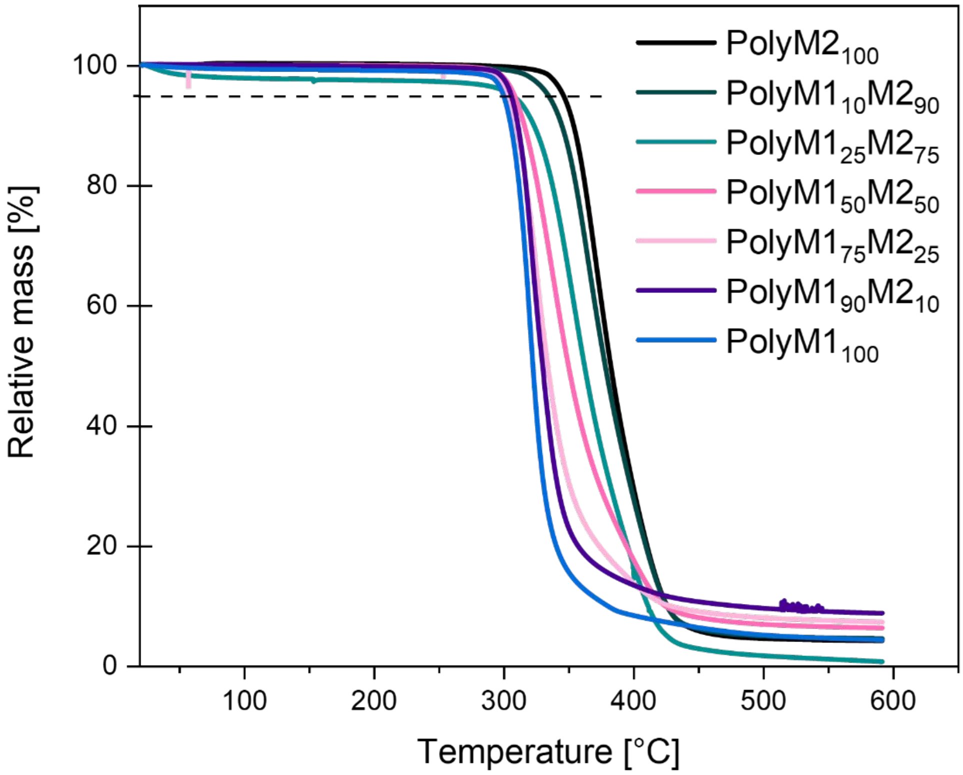

Figure S28. TGA thermograms resulted from heating at 10 °C min^{-1}up to 600 °C under a N_2 atmosphere. 5% relative mass loss is highlighted with a dashed line.

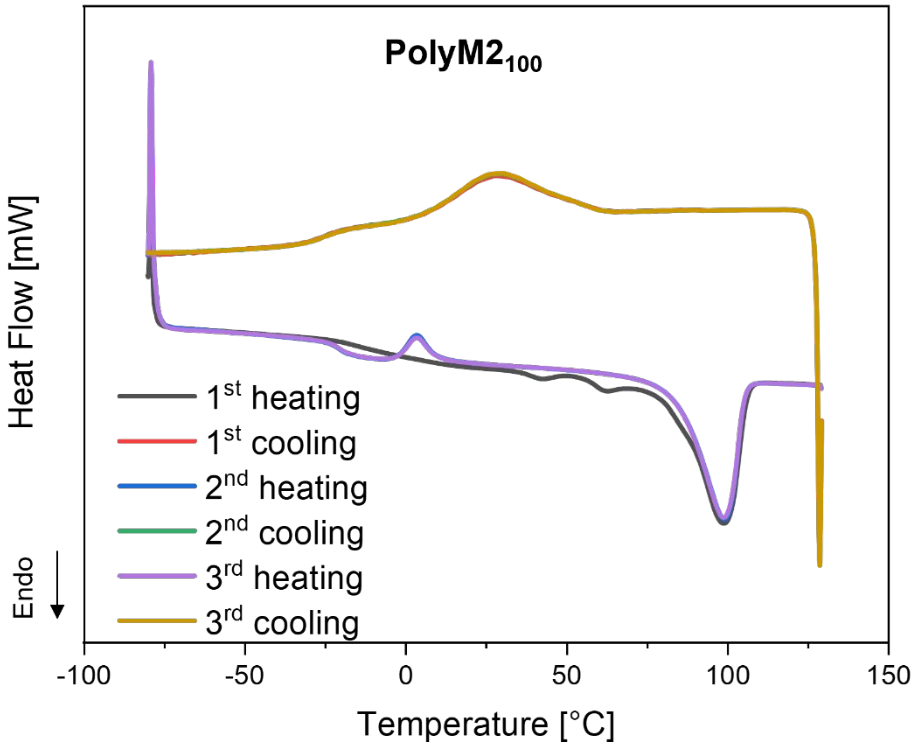

Figure S29. DSC thermograms of polyM2$_{100}$ tested between −80 °C and 130 °C at 10 °C min^{-1}.

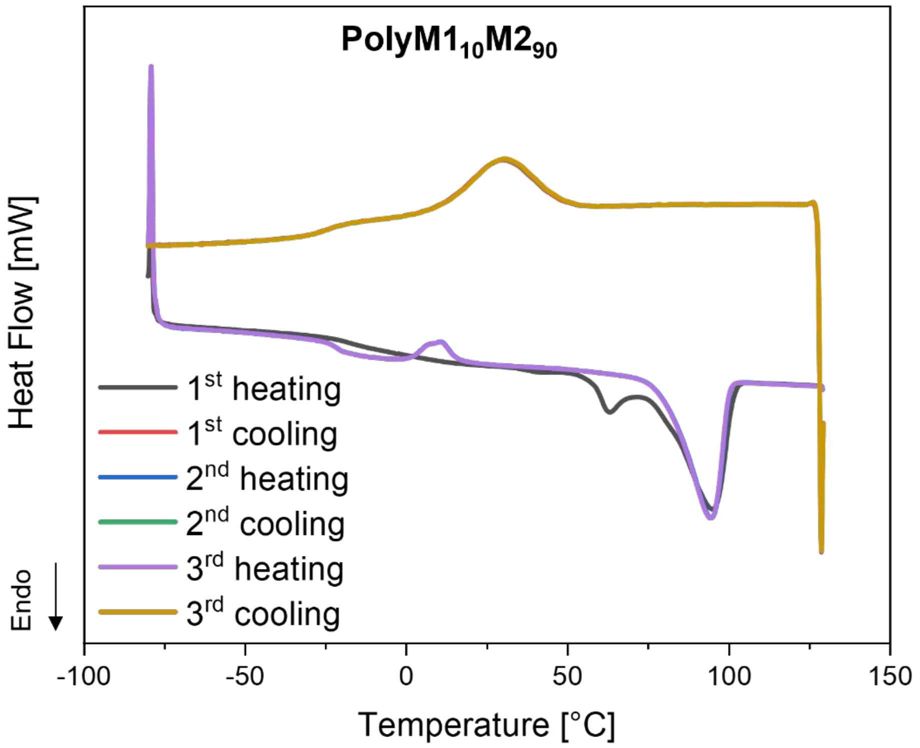

Figure S30. DSC thermograms of polyM1$_{10}$M2$_{90}$ tested between −80 °C and 130 °C at 10 °C min^{-1}.

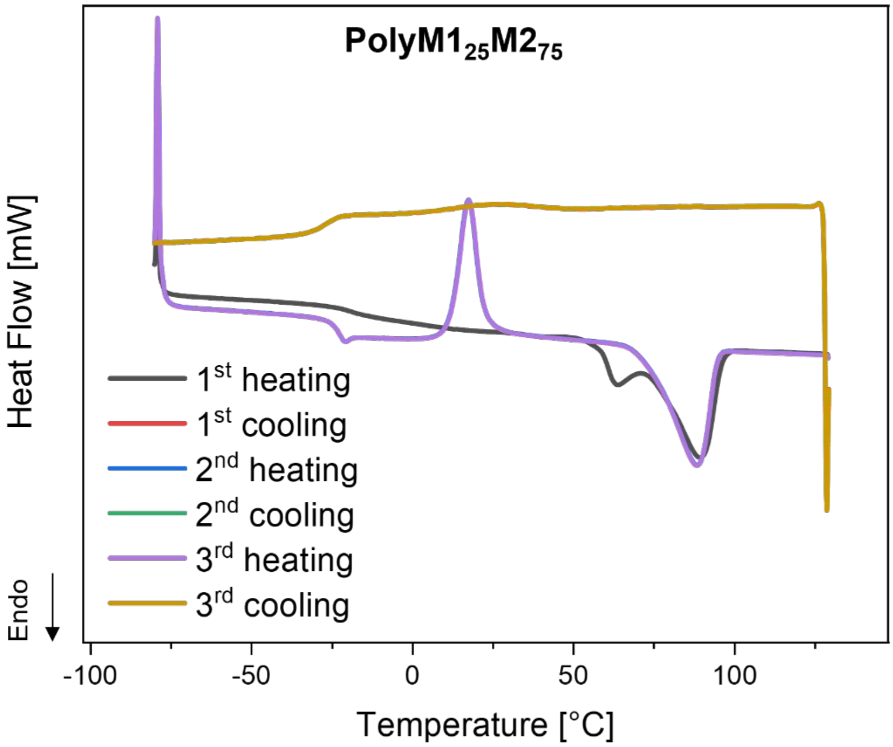

Figure S31. DSC thermograms of polyM1$_{25}$M2$_{75}$ tested between −80 °C and 130 °C at 10 °C min^{-1}.

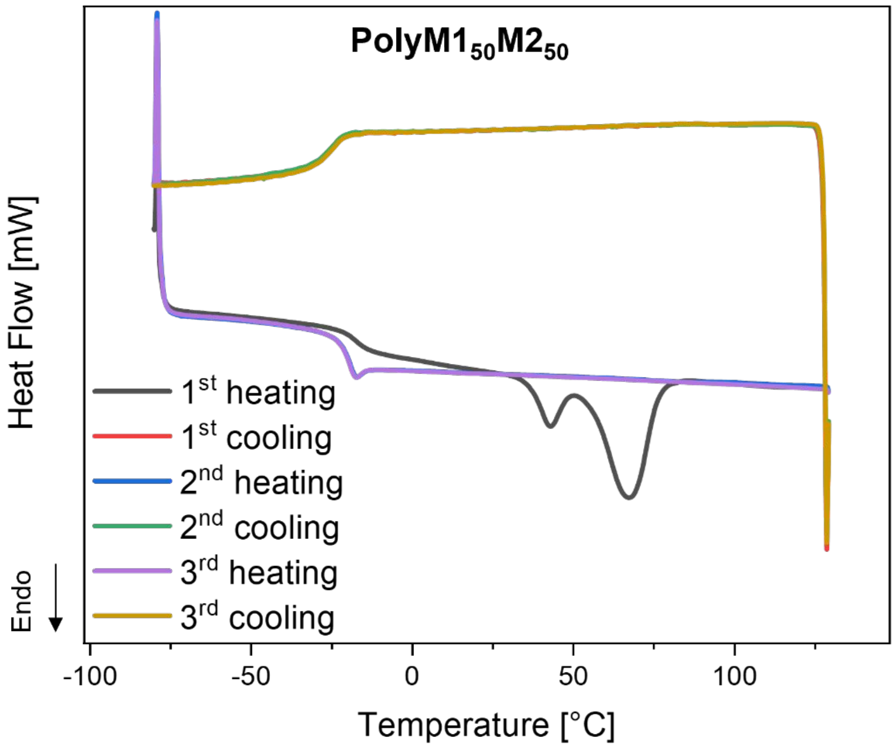

Figure S32. DSC thermograms of polyM1$_{50}$M2$_{50}$ tested between −80 °C and 130 °C at 10 °C min^{-1}.

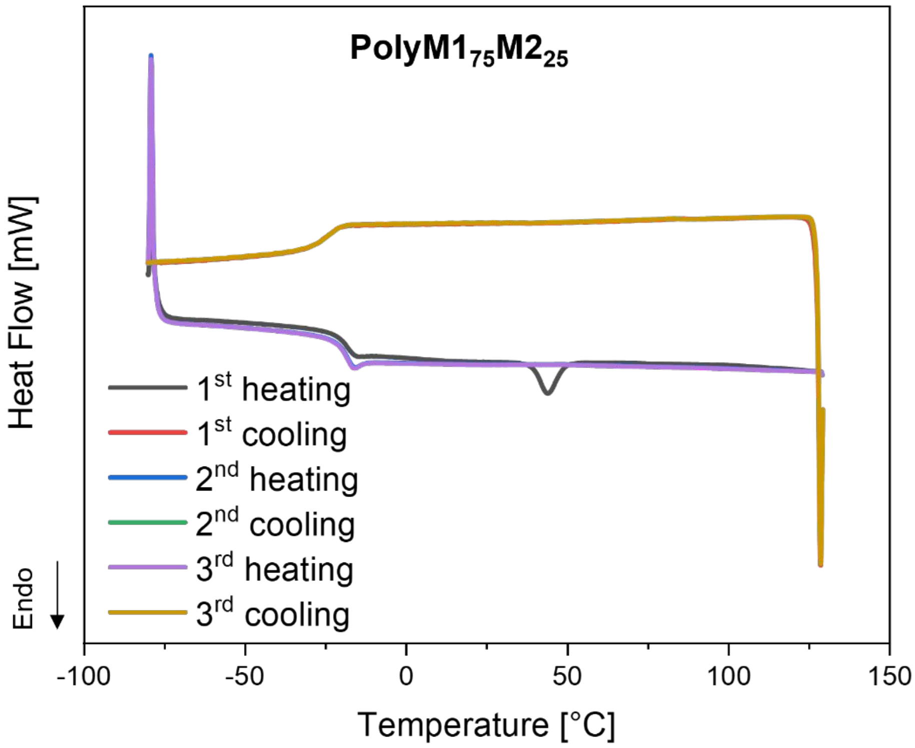

Figure S33. DSC thermograms of polyM1$_{75}$M2$_{25}$ tested between −80 °C and 130 °C at 10 °C min^{-1}.

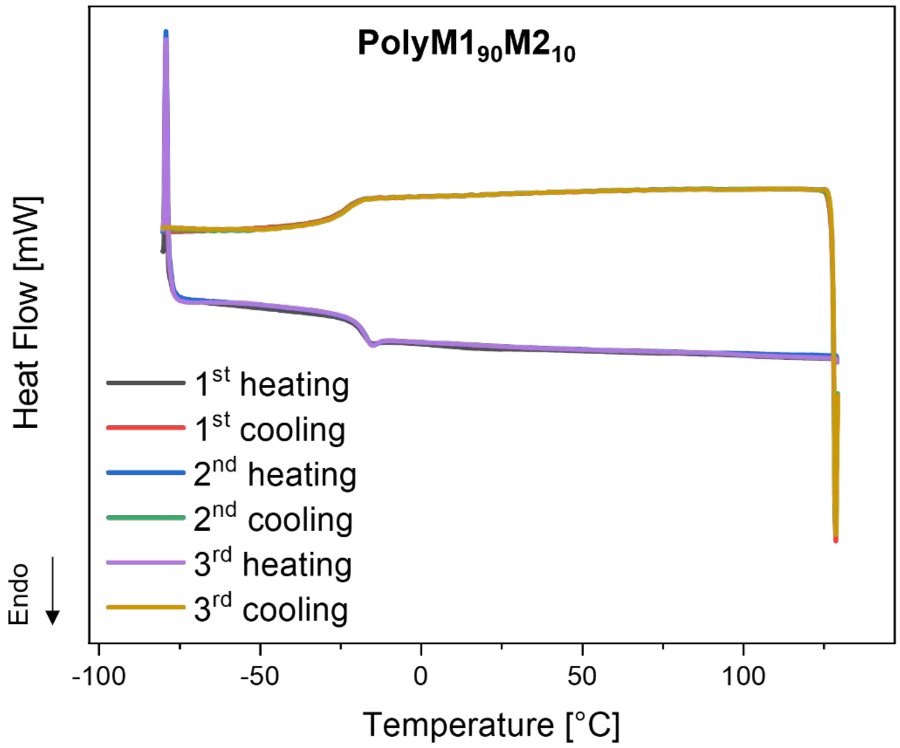

Figure S34. DSC thermograms of polyM1$_{90}$M2$_{10}$ tested between −80 °C and 130 °C at 10 °C min^{-1}.

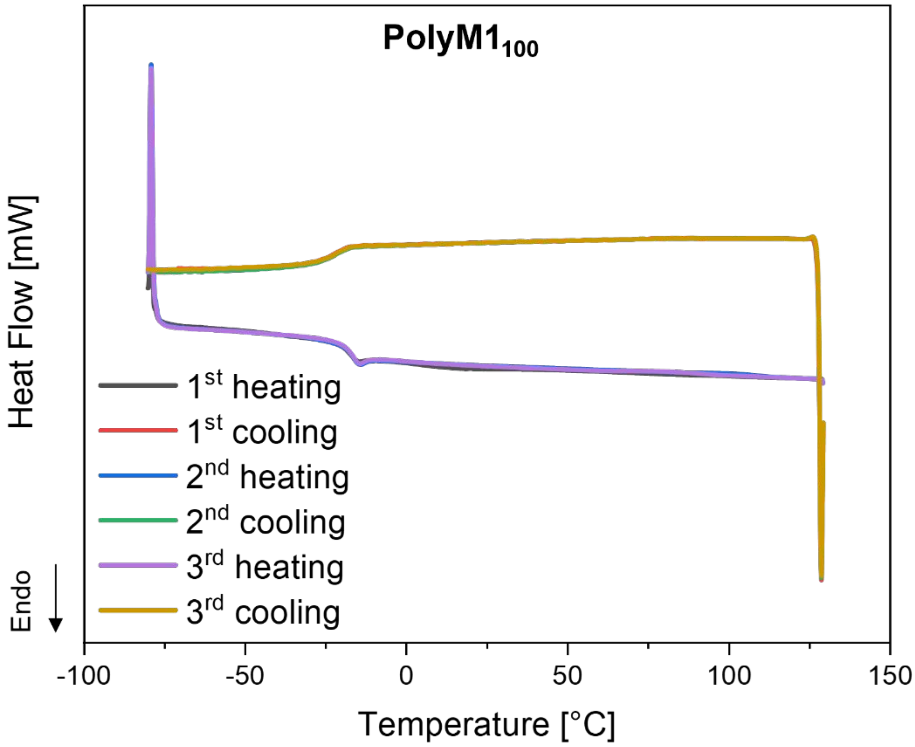

Figure S35. DSC thermograms of polyM1$_{100}$ tested between −80 °C and 130 °C at 10 °C min^{-1}.

Influence of compression molding on polymer structure and M_W

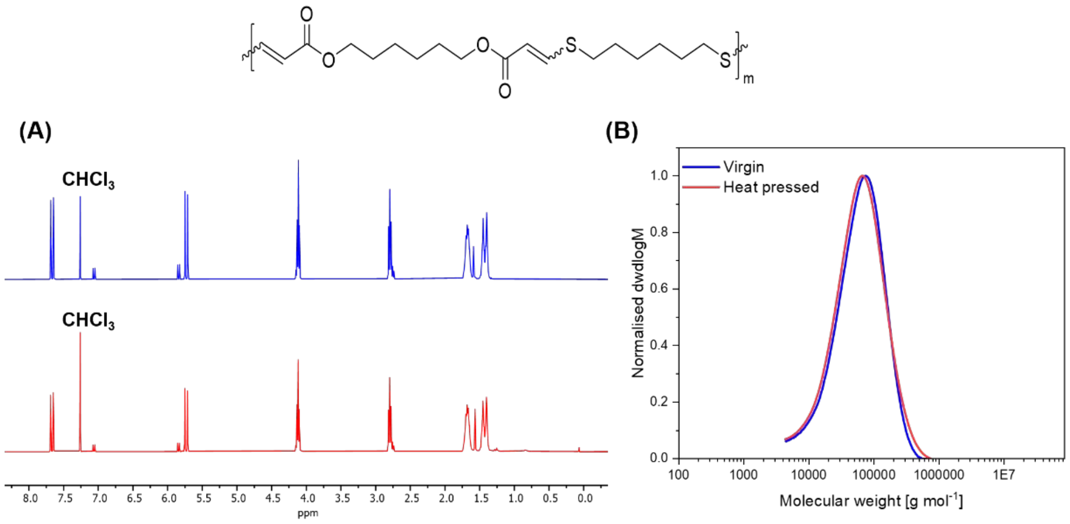

Figure S36. (A) ^{1}H NMR spectra (400 MHz, 298 K, $CDCl_3$) and (B) SEC ($CHCl_3$, 0.5% Et_3N) of polyM2$_{100}$ before (top) and after (bottom) the molding process.

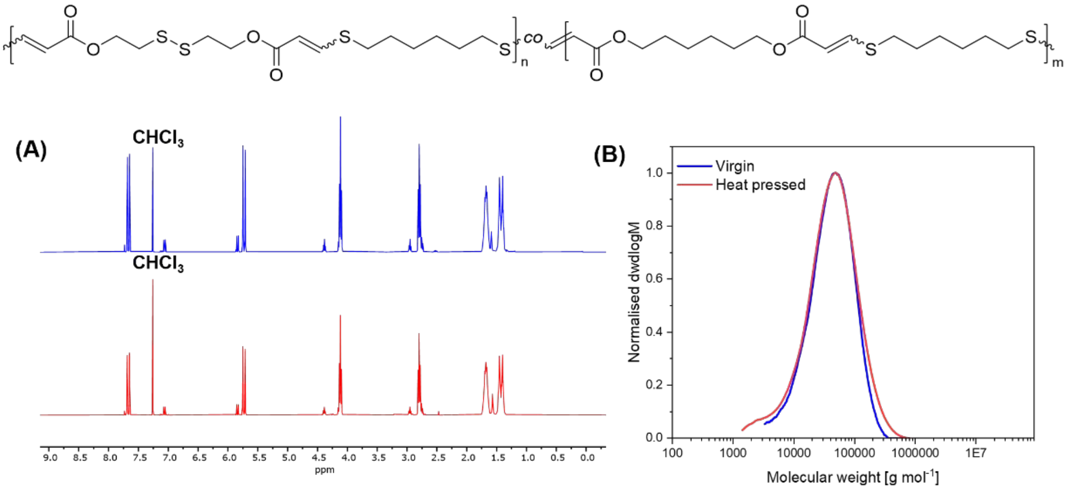

Figure S37. (A) ^{1}H NMR spectra (400 MHz, 298 K, $CDCl_3$) and (B) SEC ($CHCl_3$, 0.5% Et_3N) of polyM1$_{10}$M2$_{90}$ before (top) and after (bottom) the molding process.

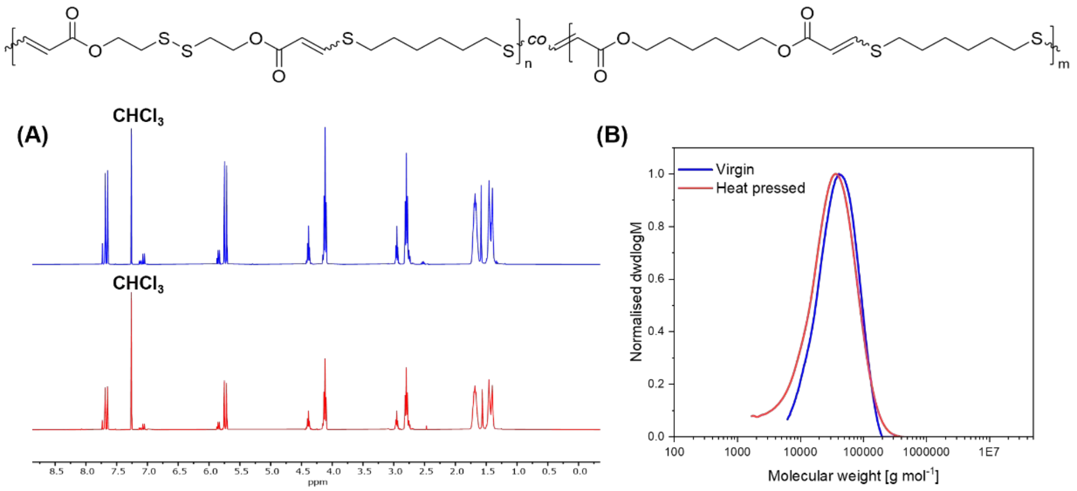

Figure S38. (A) ^{1}H NMR spectra (400 MHz, 298 K, $CDCl_3$) and (B) SEC ($CHCl_3$, 0.5% Et_3N) of polyM1$_{25}$M2$_{75}$ before (top) and after (bottom) the molding process.

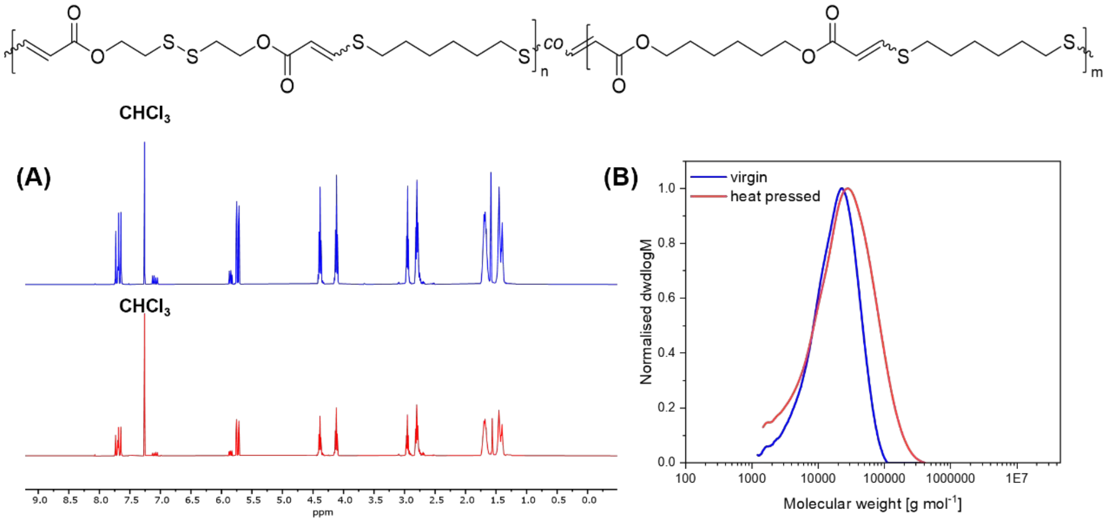

Figure S39. (A) ^{1}H NMR spectra (400 MHz, 298 K, $CDCl_3$) and (B) SEC ($CHCl_3$, 0.5% Et_3N) of polyM1$_{50}$M2$_{50}$ before (top) and after (bottom) the molding process.

Mechanical characterization of *trans* polymers of different monomer M1 content

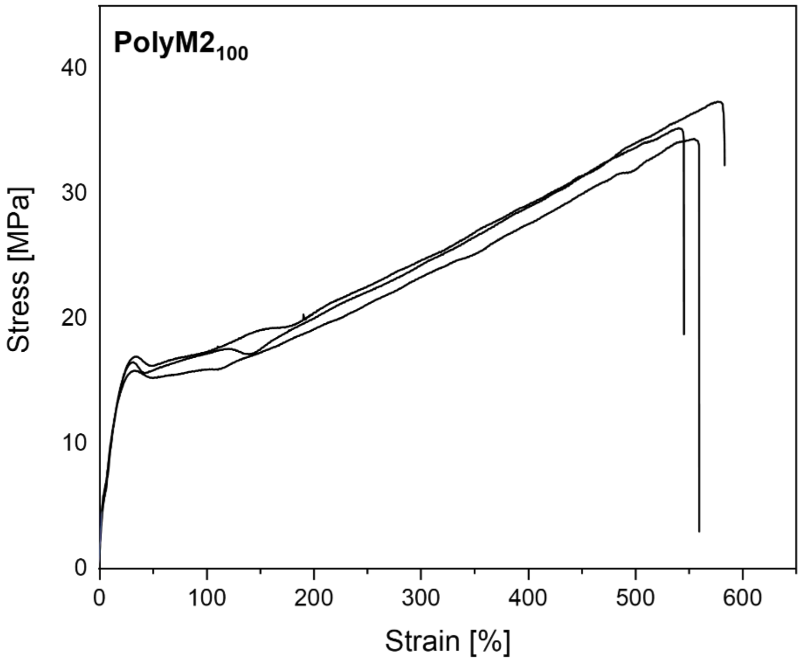

Figure S40. Stress-strain curves (10 mm·min^{-1}, 22 °C) of polyM2$_{100}$.

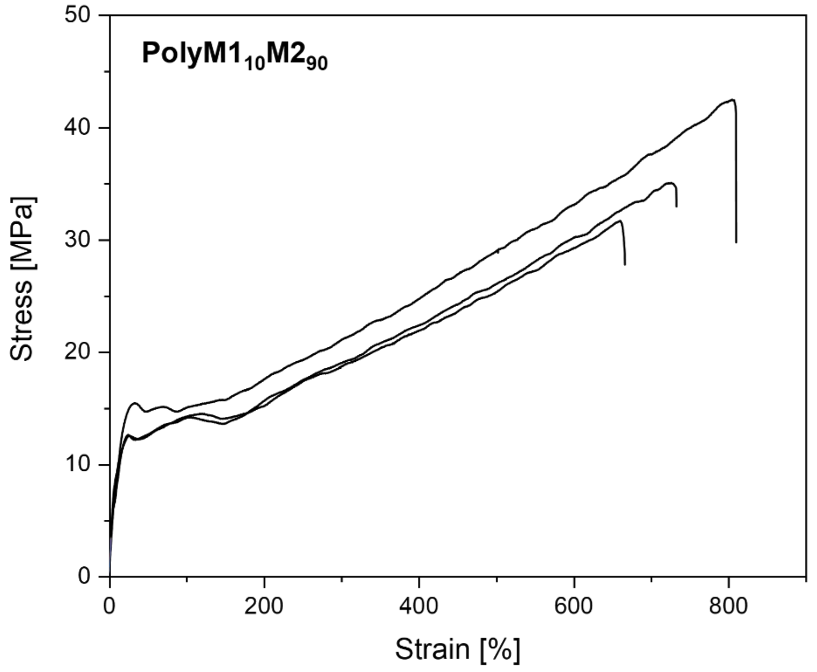

Figure S41. Stress-strain curves (10 mm·min^{-1}, 22 °C) of polyM1$_{10}$M2$_{90}$.

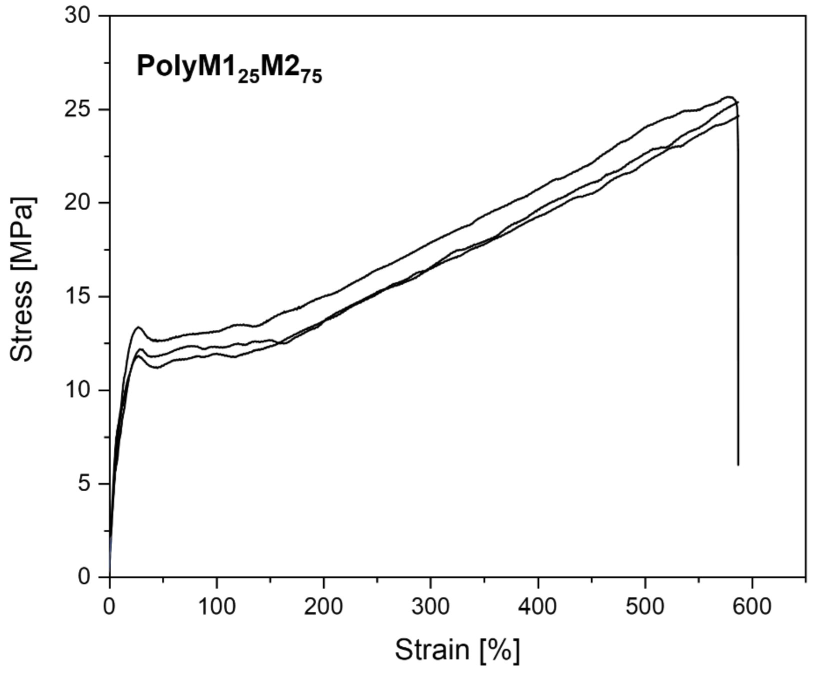

Figure S42. Stress-strain curves (10 mm·min^{-1}, 22 °C) of polyM1$_{25}$M2$_{75}$.

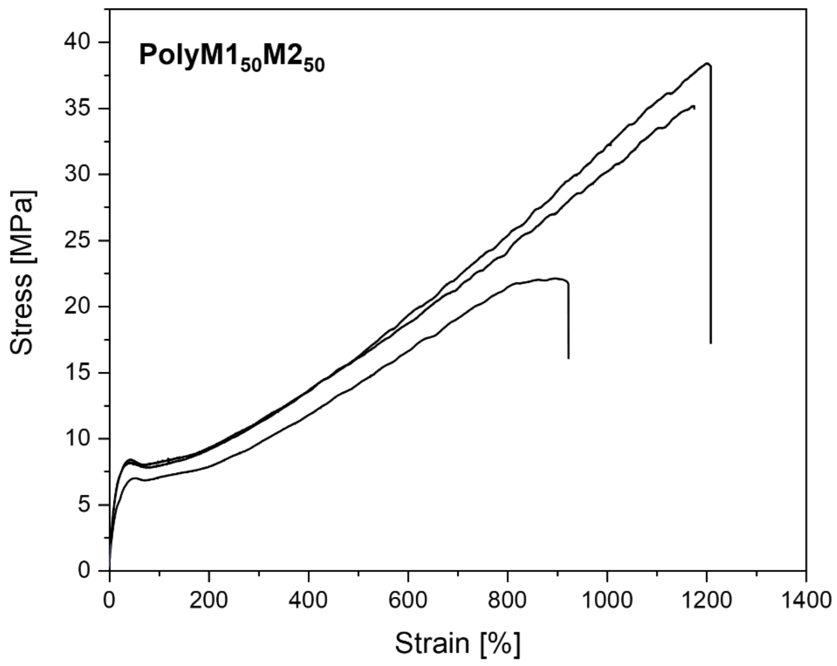

Figure S43. Stress-strain curves (10 mm·min^{-1}, 22 °C) of polyM1$_{50}$M2$_{50}$.

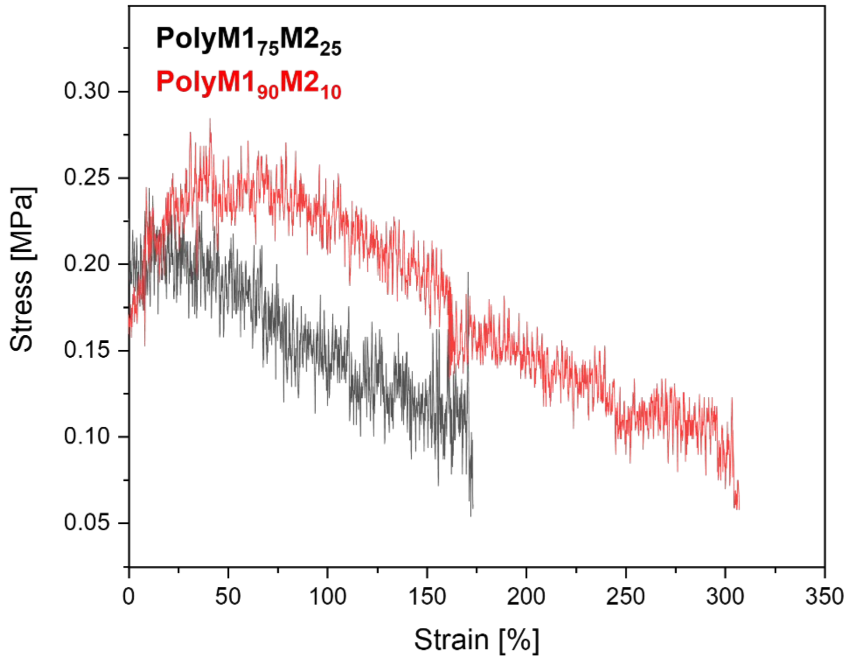

Figure S44. Stress-strain curves (10 mm·min^{-1}, 22 °C) of polyM1$_{75}$M2$_{25}$ and polyM1$_{90}$M2$_{10}$. PolyM1$_{100}$ broke upon handling.

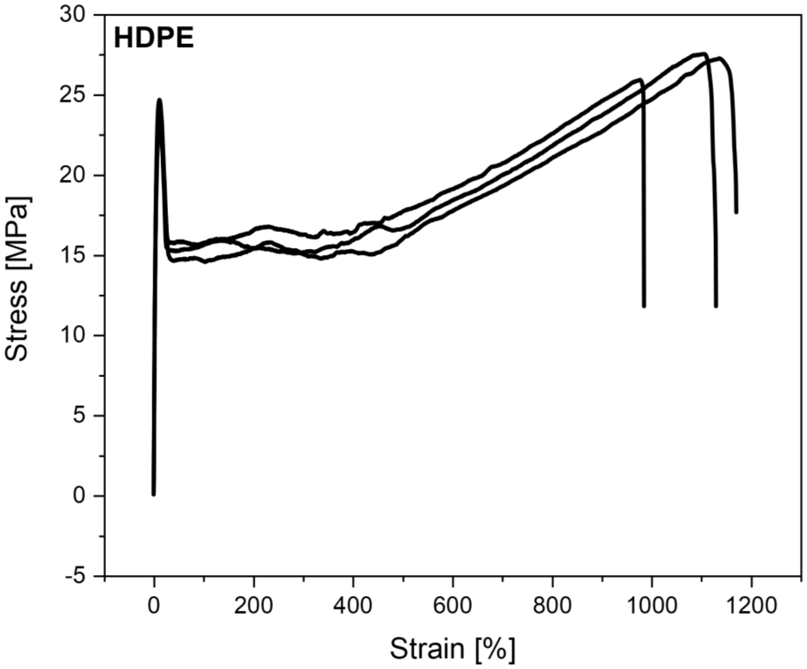

Figure S45. Stress-strain curves (10 mm·min^{-1}, 22 °C) of HDPE.

Evaluation of the influence of M_W on thermomechanical properties

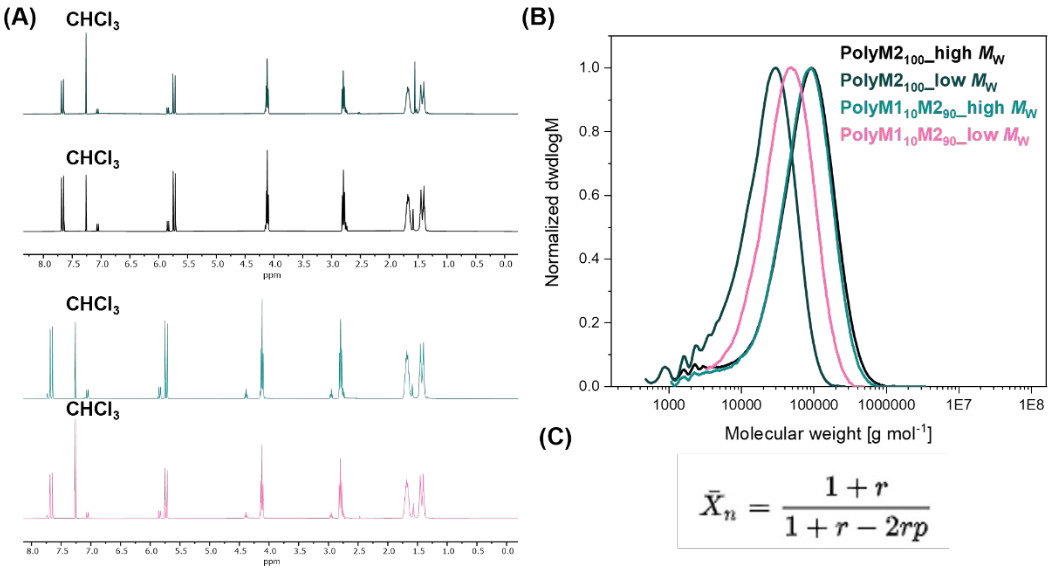

$$\bar{X}_n = \frac{1+r}{1+r-2rp}$$

Figure S46. (A) ^{1}H NMR spectra (400 MHz, 298 K, $CDCl_3$) of polyM2$_{100}$ (top) and polyM1$_{10}$M2$_{90}$ (bottom) of different molecular weights. (B) SEC ($CHCl_3$, 0.5% Et_3N) traces of polymers. Numerical values reported. (C) Extended Carother's equation: *r* is the stoichiometric ratio of reagents; *p* is the conversion which is assumed to be 100%.

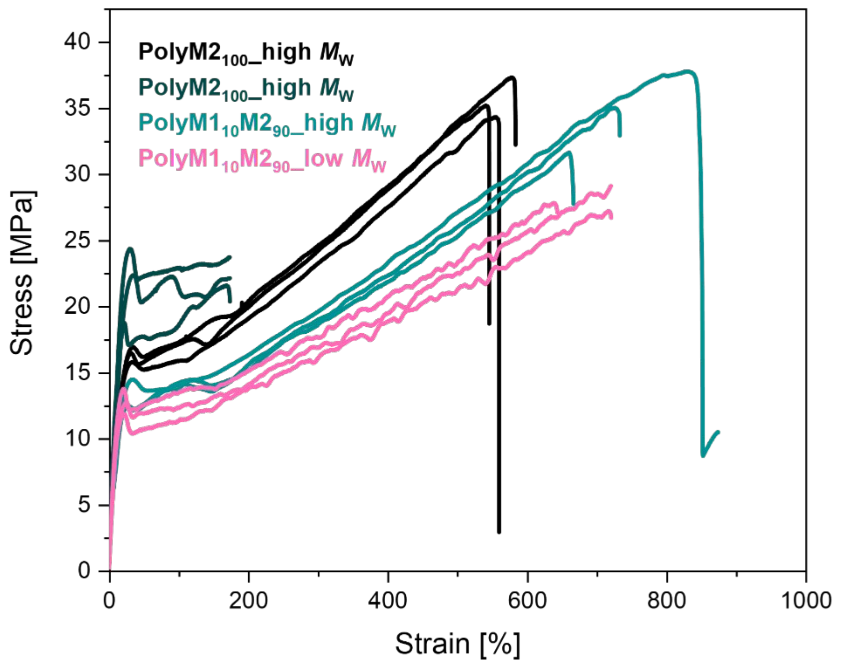

Figure S47. Stress *vs* strain curves tested at 10 mm min^{-1}, 22 °C of polyM2_{100} and polyM1_{10}M2_{90} of different M_W.

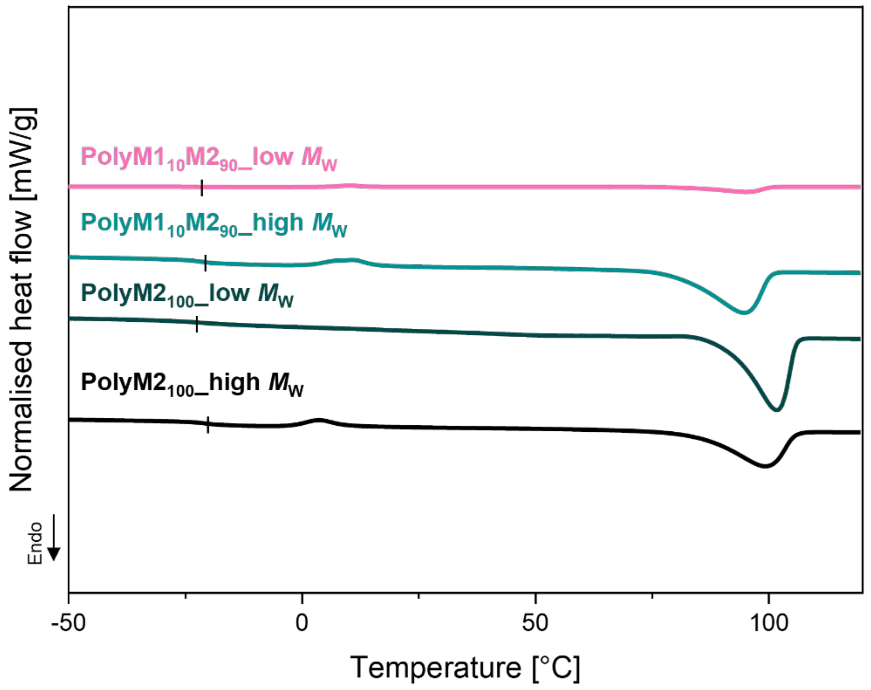

Figure S48. DSC thermograms of 2nd heating cycle tested at 10 °C min^{-1} of polyM2_{100} and polyM1_{10}M2_{90} of different M_W. Numerical values reported in table S1.

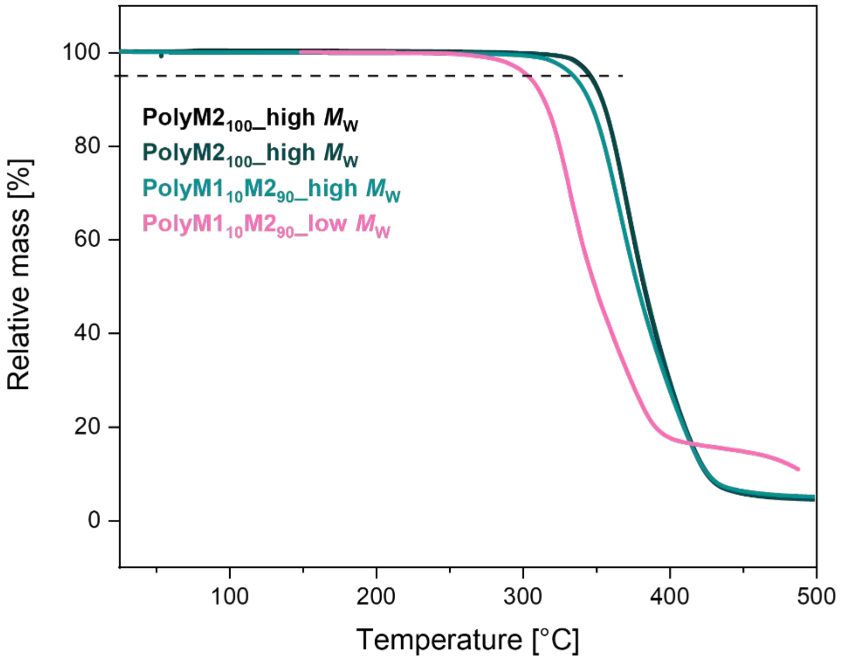

Figure S49. TGA thermograms resulted from heating at 10 °C min^{-1}up to 600 °C under a N_2 atmosphere of polyM2_{100} and polyM1_{10}M2_{90} of different M_W.

Synthesis and characterization of *cis*-polyM1$_{10}$M2$_{90}$

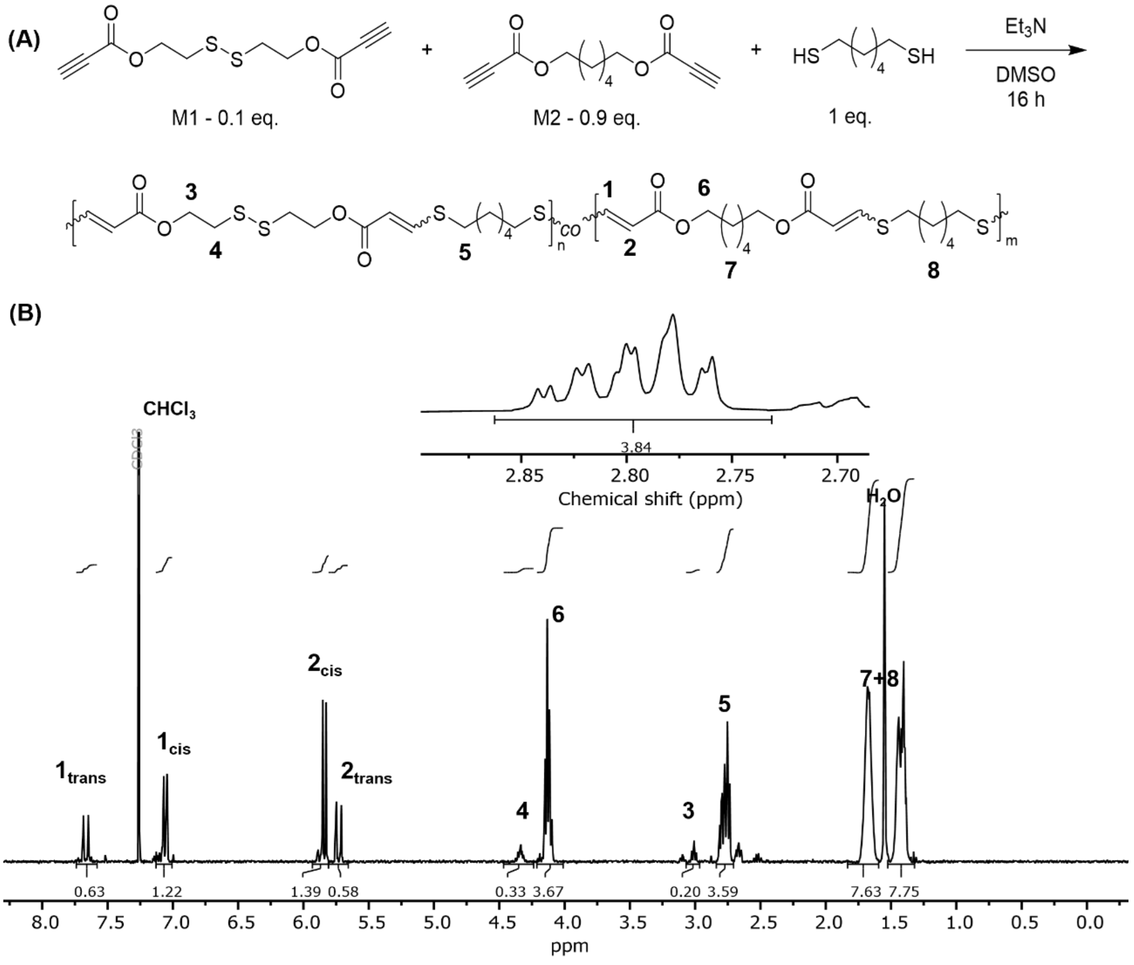

Figure S50. (A) Et$_3$N-catalyzed thiol-yne polyaddition between M1, M2 and 1,6-hexanedithiol in DMSO. (B) *Cis*-polyM1$_{10}$M2$_{90}$ – ^{1}H NMR spectrum (400 MHz, 298 K, CDCl$_3$) of high-*cis* content polymer.

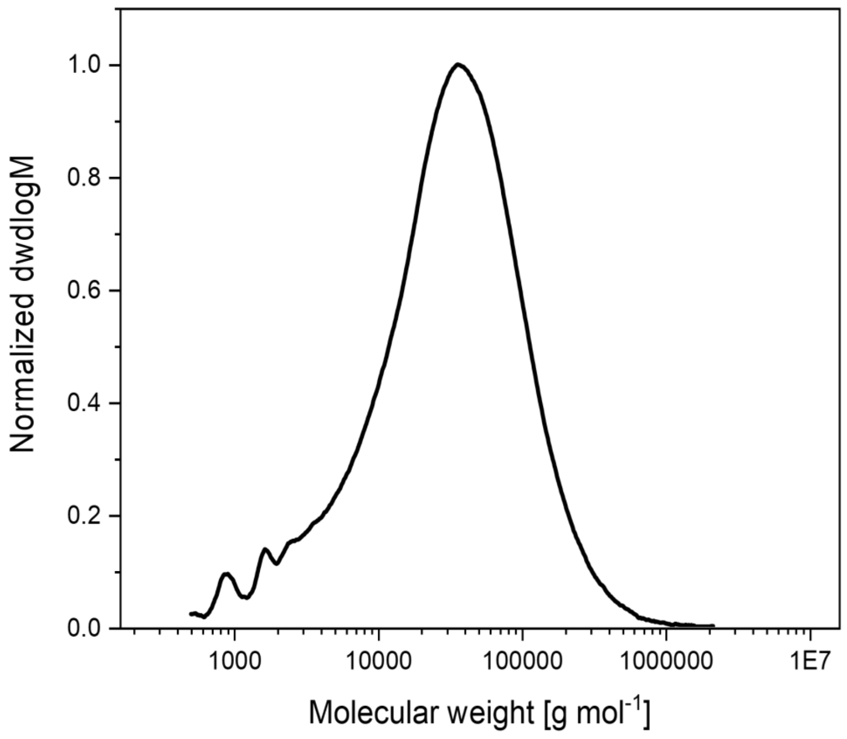

Figure S51. SEC ($CHCl_3$, 0.5% Et_3N) trace of *cis*-polyM1$_{10}$M2$_{90}$. Numerical value is reported in Table S1.

Thermomechanical characterization of *cis*-polyM1$_{10}$M2$_{90}$

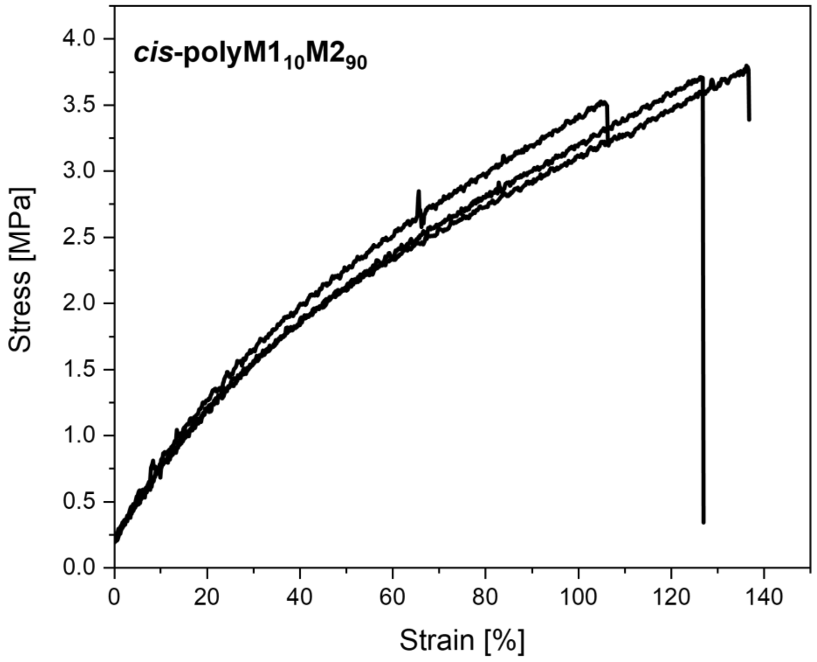

Figure S52. Stress-strain curves tested at 10 mm min^{-1}, 22 °C of *cis*-polyM1$_{10}$M2$_{90}$.

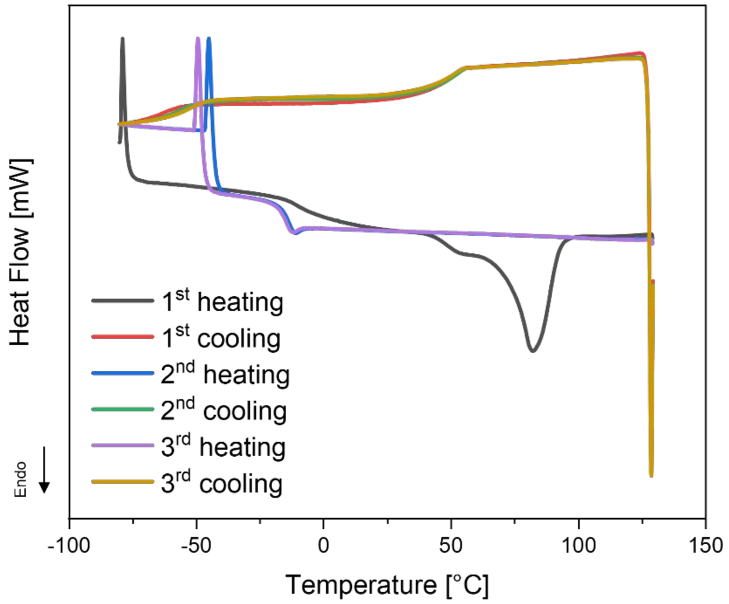

Figure S53. DSC thermograms between −80 and 130 °C at 10 °C min^{-1} of *cis*-polyM1$_{10}$M2$_{90}$.

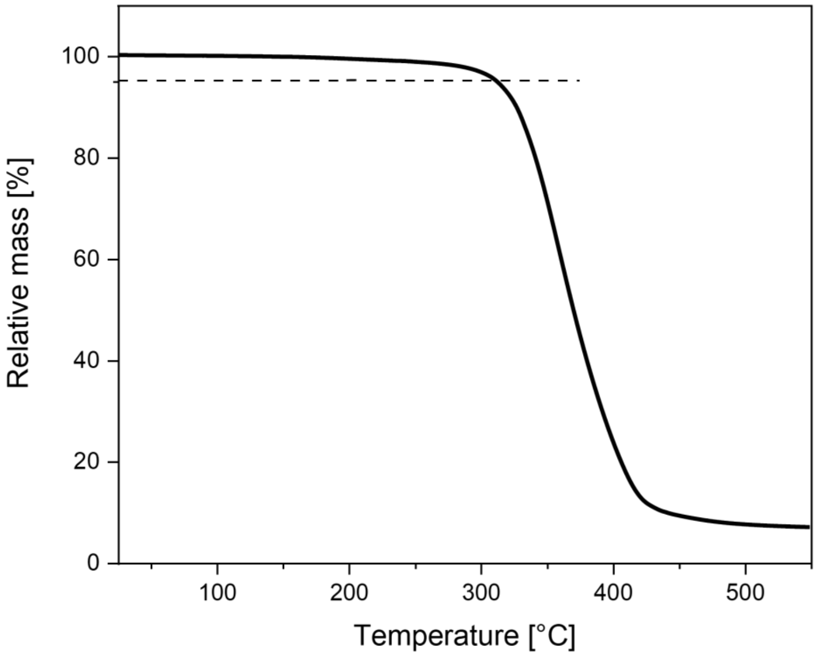

Figure S54. TGA thermograms resulted from heating at 10 °C min^{-1}up to 600 °C under a N_2 atmosphere of *cis*-poly$M1_{10}M2_{90}$. Dashed line at 95% relative mass.

Synthesis and characterization of polymers of different architecture with a fixed content of monomer M1

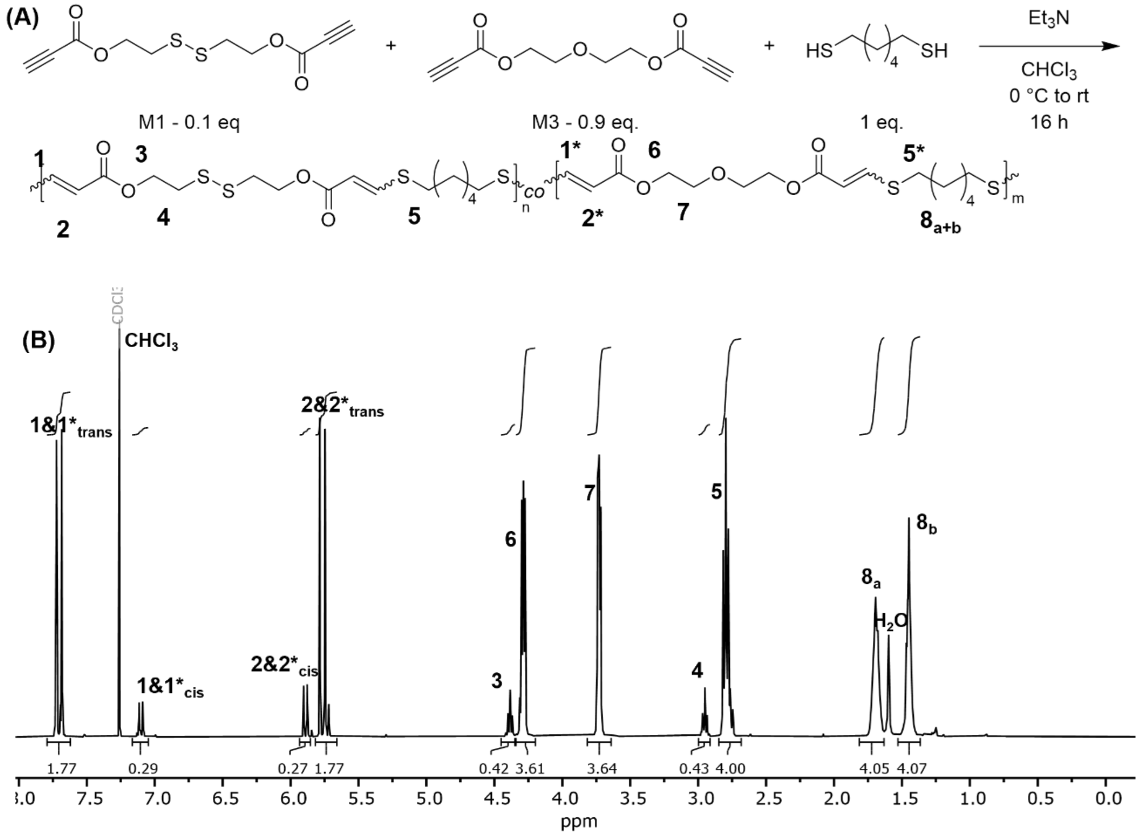

Figure S55. (A) Et_3N-catalyzed thiol-yne polyaddition between M1, M3 and 1,6-hexanedithiol. (B) PolyM1$_{10}$M3$_{90}$ – ^{1}H NMR spectrum (400 MHz, 298 K, $CDCl_3$).

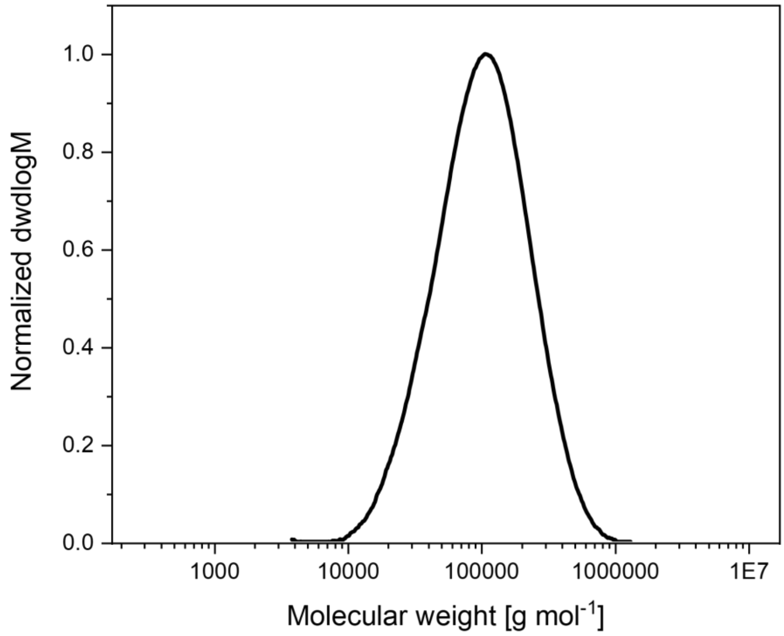

Figure S56. SEC ($CHCl_3$, 0.5% Et_3N) trace of polyM1$_{10}$M3$_{90}$. Numerical values reported in table S1.

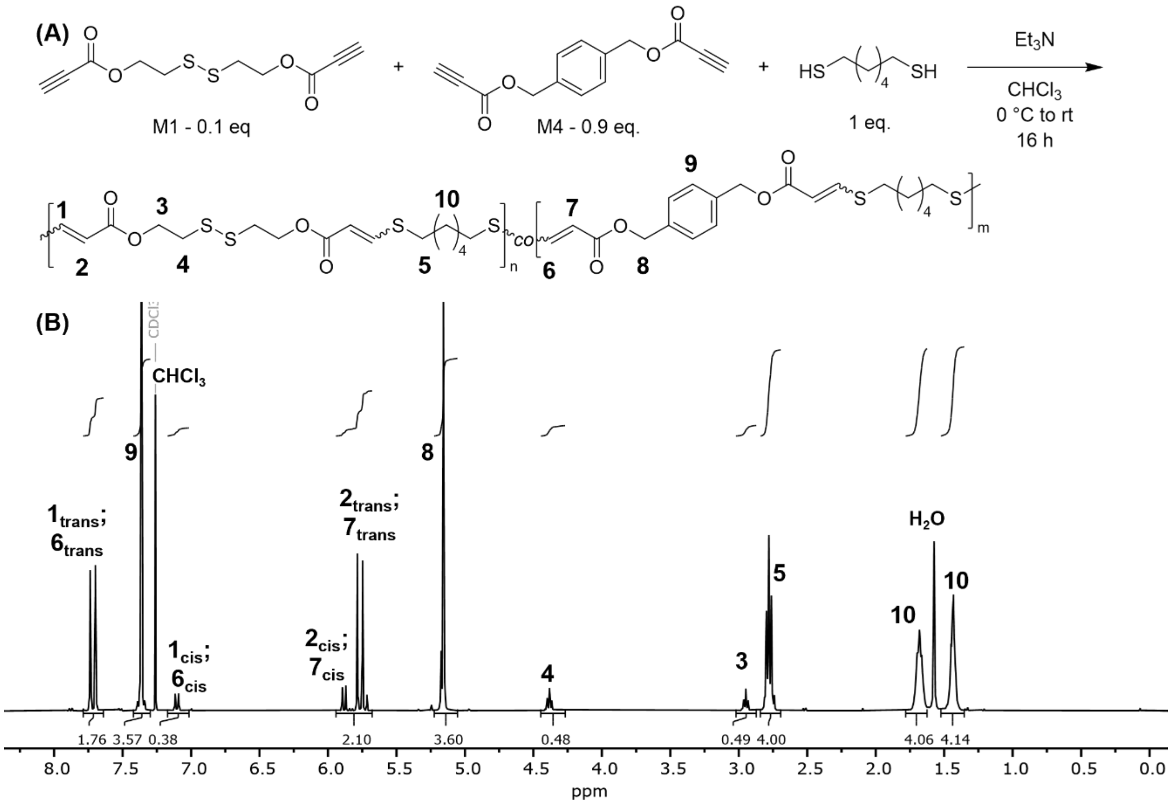

Figure S57. (A) Et_3N-catalyzed thiol-yne polyaddition between M1, M4 and 1,6-hexanedithiol. (B) PolyM1$_{10}$M4$_{90}$ – ^{1}H NMR spectrum (400 MHz, 298 K, $CDCl_3$).

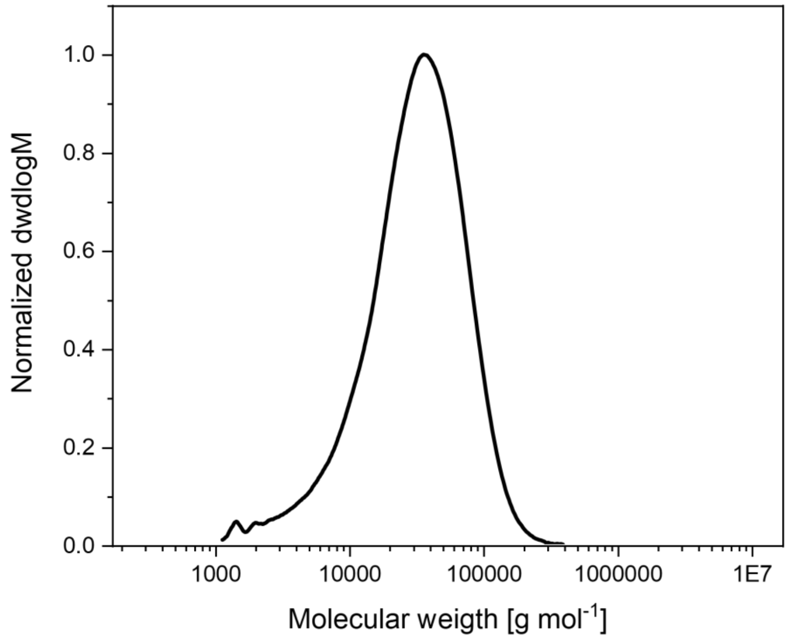

Figure S58. SEC ($CHCl_3$, 0.5% Et_3N) trace of polyM1_{10}M4_{90}. Numerical values reported in table S1.

Thermomechanical properties of polymers of different architectures with fixed content of monomer M1

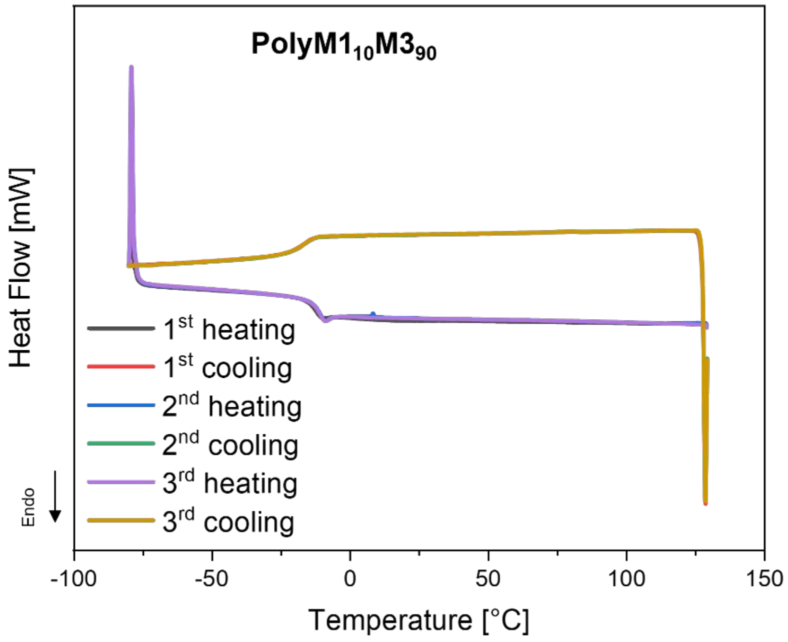

Figure S59. DSC thermograms of polyM1_{10}M3_{90} tested between −80 °C and 130 °C at 10 °C min^{-1}.

.

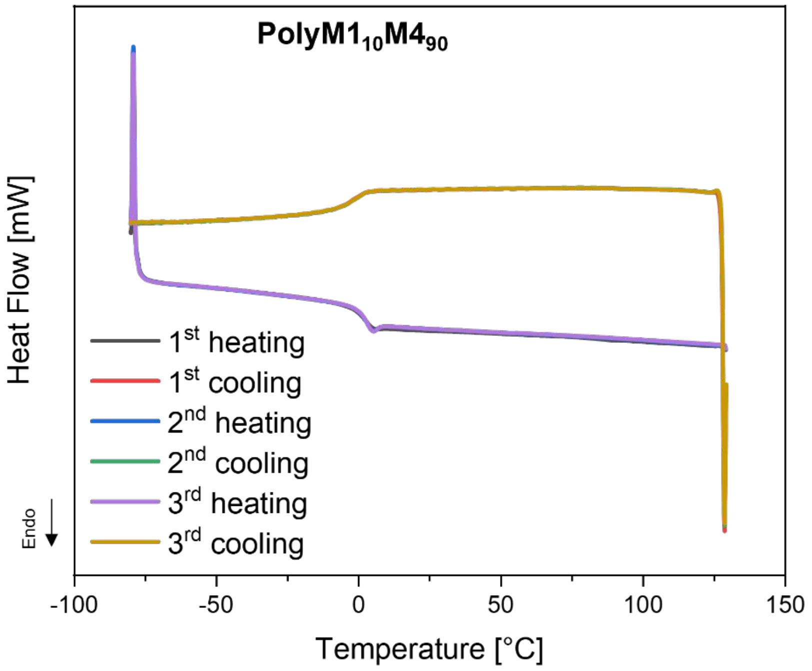

Figure S60. DSC thermograms of polyM1$_{10}$M4$_{90}$ tested between −80 °C and 130 °C at 10 °C min^{-1}.

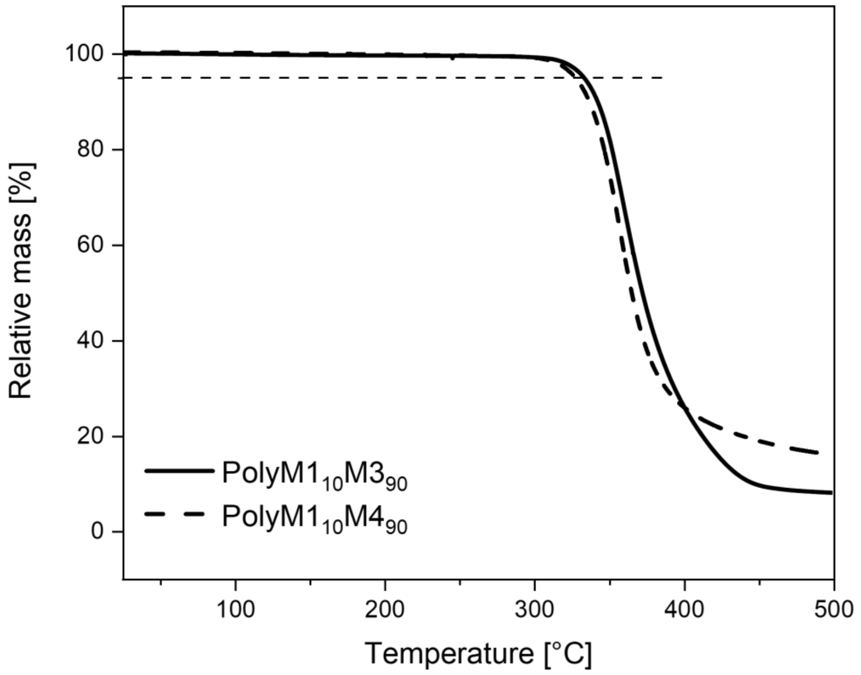

Figure S61. PolyM1$_{10}$M3$_{90}$ and PolyM1$_{0}$M4$_{90}$ TGA thermograms resulted from heating at 10 °C min^{-1} up to 600 °C under a N_2 atmosphere. Dashed line at 95% relative mass.

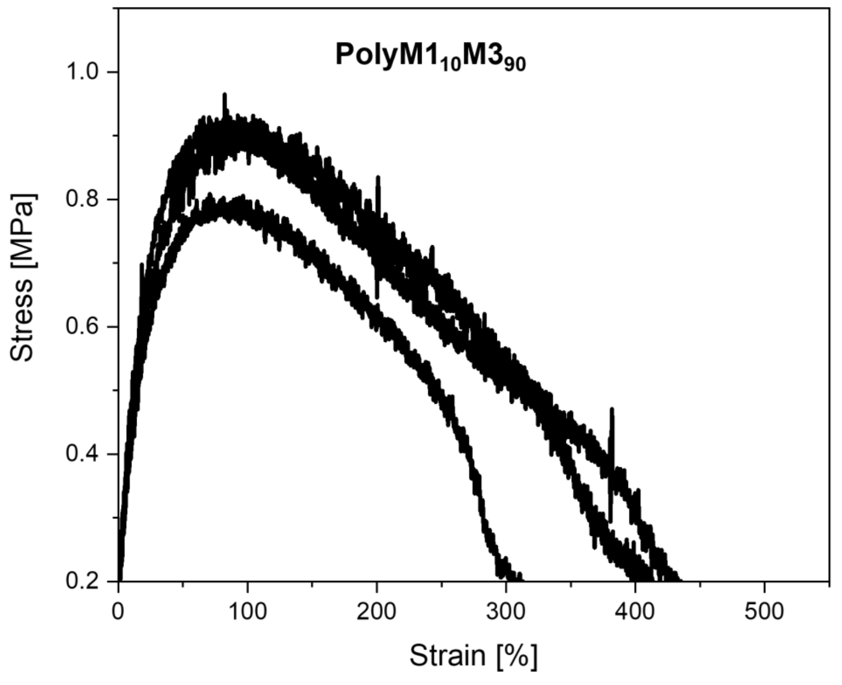

Figure S62. Stress-strain curves tested at 10 mm min^{-1}, 22 °C of polyM1$_{10}$M3$_{90}$.

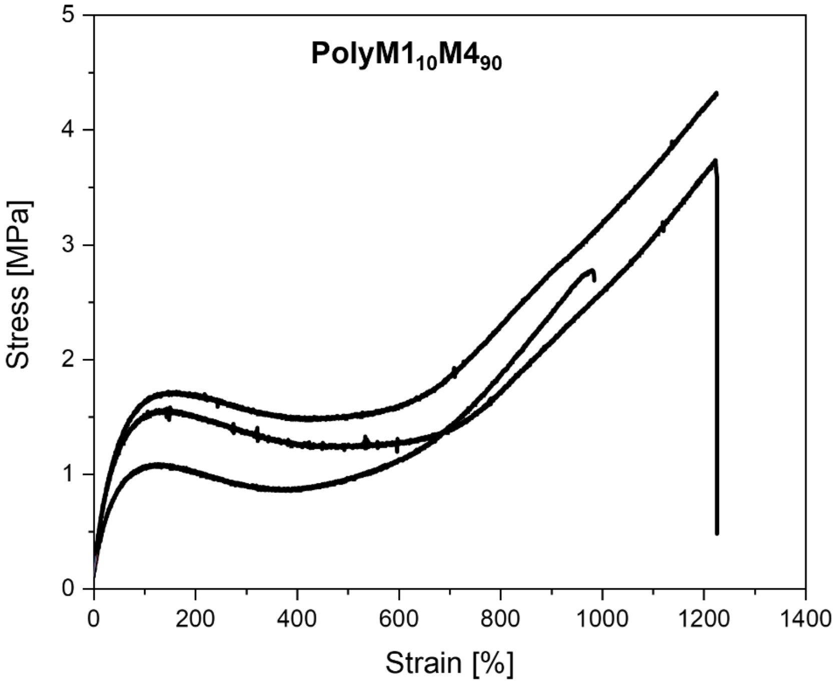

Figure S63. Stress-strain curves tested at 10 mm min^{-1}, 22 °C of polyM1_{10}M4_{90}.

Table S1. Table summarizing M_W and thermomechanical properties of the synthesized polymers.

Entry	Name	M_W [kg/mol]	$Đ_M$	T_g [°C]	T_c [°C]	T_m [°C]	$T_{d,5\%}$ [°C]	Young's Modulus (*E*) [MPa]	Stress at break [MPa]	Strain at break [%]	Strain energy density [J·m^{-3}]
1	*PolyM2$_{100}$*	102.9	3.59	−20.35	3.82	99.71	346.8	146.3 ± 15.9	35.6 ± 1.6	560.7 ± 19.1	133.5 ± 9.5
2	*PolyM2$_{100}$_low M_W*	28.9	2.72	−22.67	n.a.	102.08	346.32	231.5 ± 29.1	22.4 ± 1.1	172.5 ± 1	34.6 ± 32
3	*PolyM1$_{10}$M2$_{90}$*	95.9	2.89	−21.01	10.65	95.05	335.08	124.6 ± 16.5	36.3 ± 5.5	733.1 ± 72.8	167.9 ± 37.8
4	*PolyM1$_{10}$M2$_{90}$ _low M_W*	55.5	1.97	−21.66	10.34	95.48	303.84	116.3 ± 8.7	27.9 ± 0.9	692.9 ± 44.5	128.6 ± 7.6
5	*Cis_PolyM1$_{10}$M2$_{90}$*	56.0	4.87	−13.18	n.a.	n.a.	312.01	n.a.	3.7 ± 0.2	123.1 ± 16.1	2.8 ± 0.5
6	*PolyM1$_{10}$M3$_{90}$*	136.0	1.84	−10.35	n.a.	n.a.	333.69	n.a.	n.a.	n.a.	n.a.
7	*PolyM1$_{10}$M4$_{90}$*	42.0	2.33	3.84	n.a.	n.a.	327.87	3.6 ± 0.8	3.6 ± 0.8	1145.4 ± 139.7	20.4 ± 7.1

References

(1) Giannantonio, D.; Brandolese, A.; Dove, A. P. Tuneable and degradable thermosets possessing dynamic aliphatic disulfide bonds via stereoselective thiol–yne polymerisation. *Polym. Chem.* **2025**, 10.1039/D4PY01195C. DOI: 10.1039/D4PY01195C.

(2) June, S. M.; Bissel, P.; Long, T. E. Segmented block copolyesters using click chemistry. *J. Polym. Sci., Part A: Polym. Chem.* **2012**, *50* (18), 3797-3805. DOI: https://doi.org/10.1002/pola.26168.

(3) Ren, Y.; Dai, W.; Guo, S.; Dong, L.; Huang, S.; Shi, J.; Tong, B.; Hao, N.; Li, L.; Cai, Z.; Dong, Y. Clusterization-Triggered Color-Tunable Room-Temperature Phosphorescence from 1,4-Dihydropyridine-Based Polymers. *J. Am. Chem. Soc.* **2022**, *144* (3), 1361-1369. DOI: 10.1021/jacs.1c11607.
